# Supplementary material for: Rapid and sensitive acute leukemia classification and diagnosis platform using deep learning-assisted SERS detection
Source: Cell Rep Med. 2025 Sep 8;6(9):102320. doi: 10.1016/j.xcrm.2025.102320 (PMC12490261; doi:10.1016/j.xcrm.2025.102320)
Supplement: Document S1. Figures S1–S25 and Tables S1–S6 [file mmc1.pdf]

Cell Reports Medicine, Volume *6*

Supplemental information

Rapid and sensitive acute leukemia classification and diagnosis platform using deep learning-assisted SERS detection

Dongjie Zhang, Zhaoyang Cheng, Yali Song, Huandi Li, Lin Shi, Nan Wang, Yingwen Peng, Renan Chen, Nianzheng Sun, Min Han, Fengjiao Hu, Chuntao Zong, Rui Zhang, Si Chen, Conghui Zhu, Xiaoli Zhang, Xiaobo Li, Xiaopeng Ma, Changbei Shi, Xiaofei Zhang, Rui Liu, Ziqi Ren, Lin Wang, Qi Zeng, Tingting Zeng, and Xueli Chen

Supplemental Information

Rapid and sensitive acute leukemia classification and diagnosis platform using deep learning-assisted SERS detection

Dongjie Zhang, Zhaoyang Cheng, Yali Song, Huandi Li, Lin Shi, Nan Wang, Yingwen Peng, Renan Chen, Nianzheng Sun, Min Han, Fengjiao Hu, Chuntao Zong, Rui Zhang, Si Chen, Conghui Zhu, Xiaoli Zhang, Xiaobo Li, Xiaopeng Ma, Changbei Shi, Xiaofei Zhang, Rui Liu, Ziqi Ren, Lin Wang, Qi Zeng, Tingting Zeng, and Xueli Chen

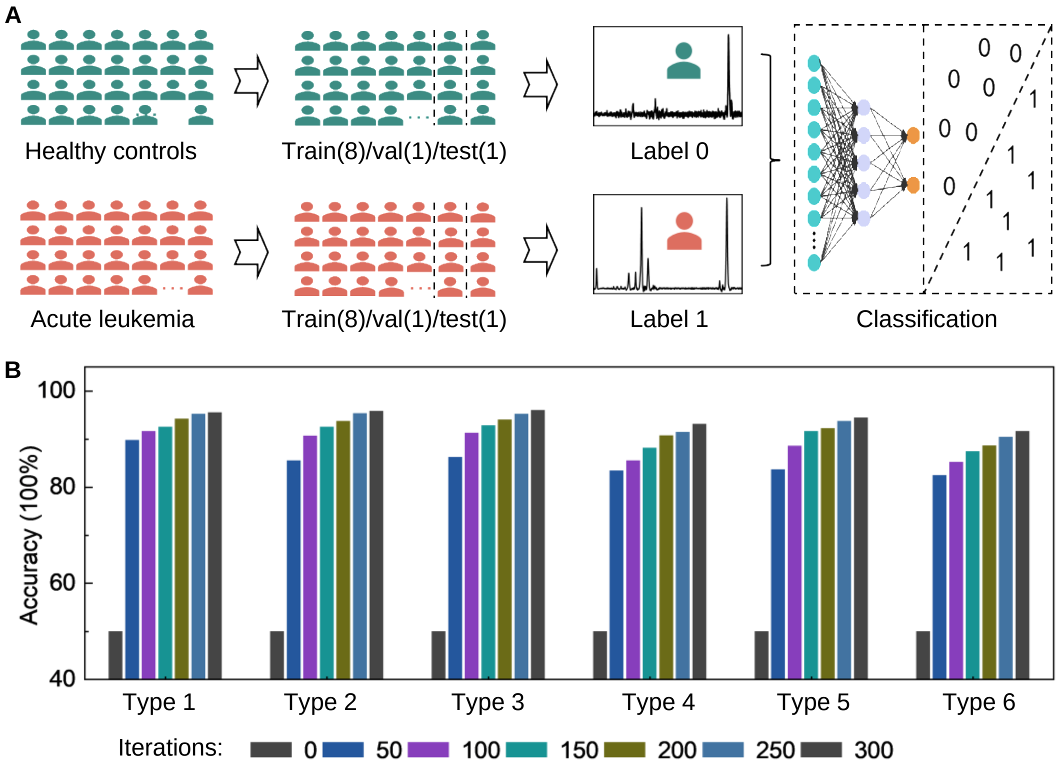

Figure S1. The strategy of dataset partitioning and classification model optimization, related to Figure 1. **(A)** The partitioning of training set and test set for binary classification. **(B)** Validation accuracy based on different iterative training of transformer algorithm with various classifications: healthy control or AL (type1), AML or ALL (type2), AML subtypes (type3), B-ALL or T-ALL (type4), B-ALL chromosome normal or abnormal (type5), and BCR/ABL1 fusion gene positive or negative (type6).

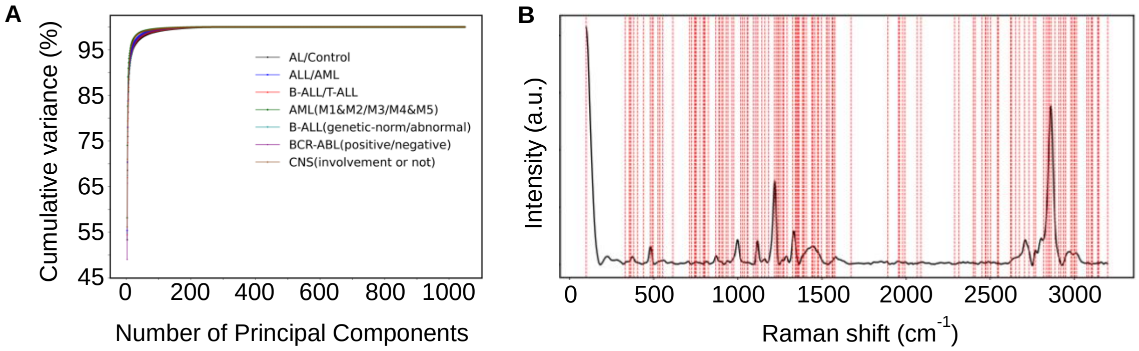

Figure S2. The extraction of spectral features used for classification, related to Figure 1. (A) the relationship between cumulative variance and the number of principal components. **(B)** visualization of the extracted key features on the original spectrum.

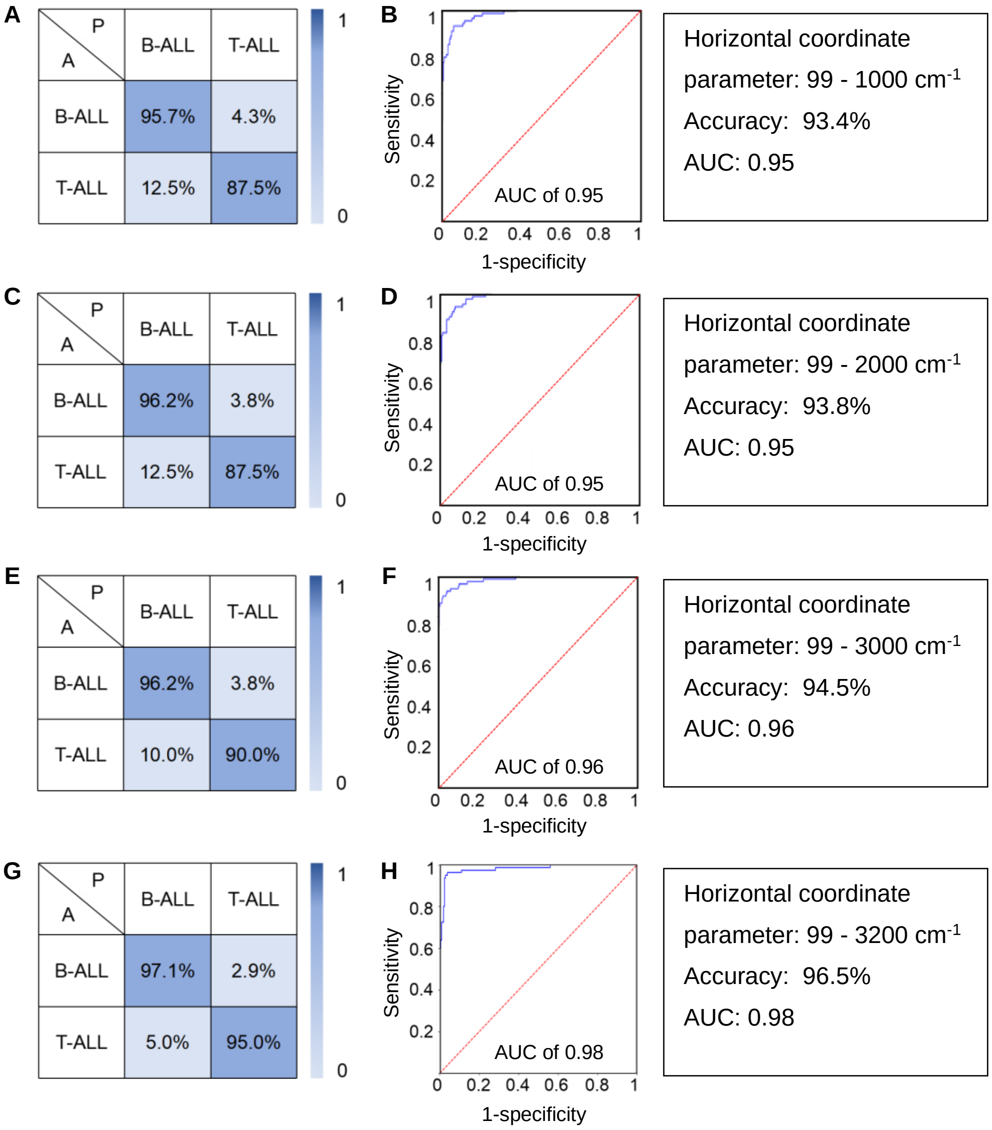

Figure S3. The effects of the selected horizontal coordinate parameters on classification results, related to Figure 1. For the binary classification of B-ALL and T-ALL, the range of the horizontal coordinate for 2-D images were: **(A-B)** 99-1000, **(C-D)** 99-2000, **(E-F)** 99-3000, and **(G-H)** 99-3200 cm^{-1}, respectively. The confusion matrix and ROCs were used to evaluate the classification performance.

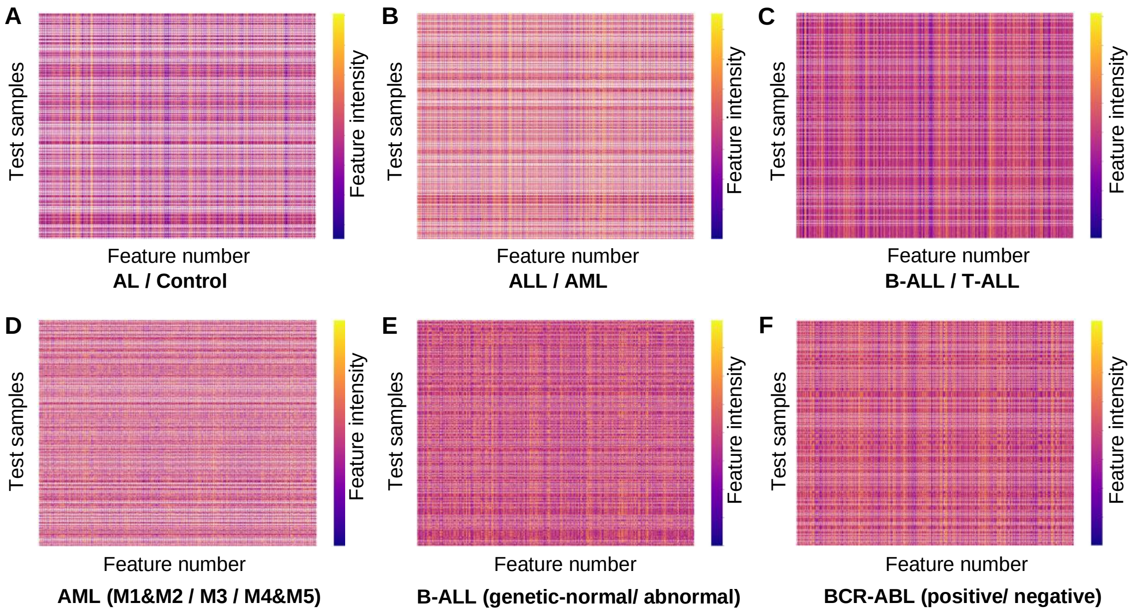

Figure S4. The visualization attention maps for various classifications, related to Figure 1. (A) AL / Control. **(B)** ALL / AML. **(C)** B-ALL / T-ALL. **(D)** AML (M1&M2 / M3 / M4&M5). **(E)** B-ALL (genetic-normal/ abnormal). **(F)** BCR-ABL fusion gene positive / negative.

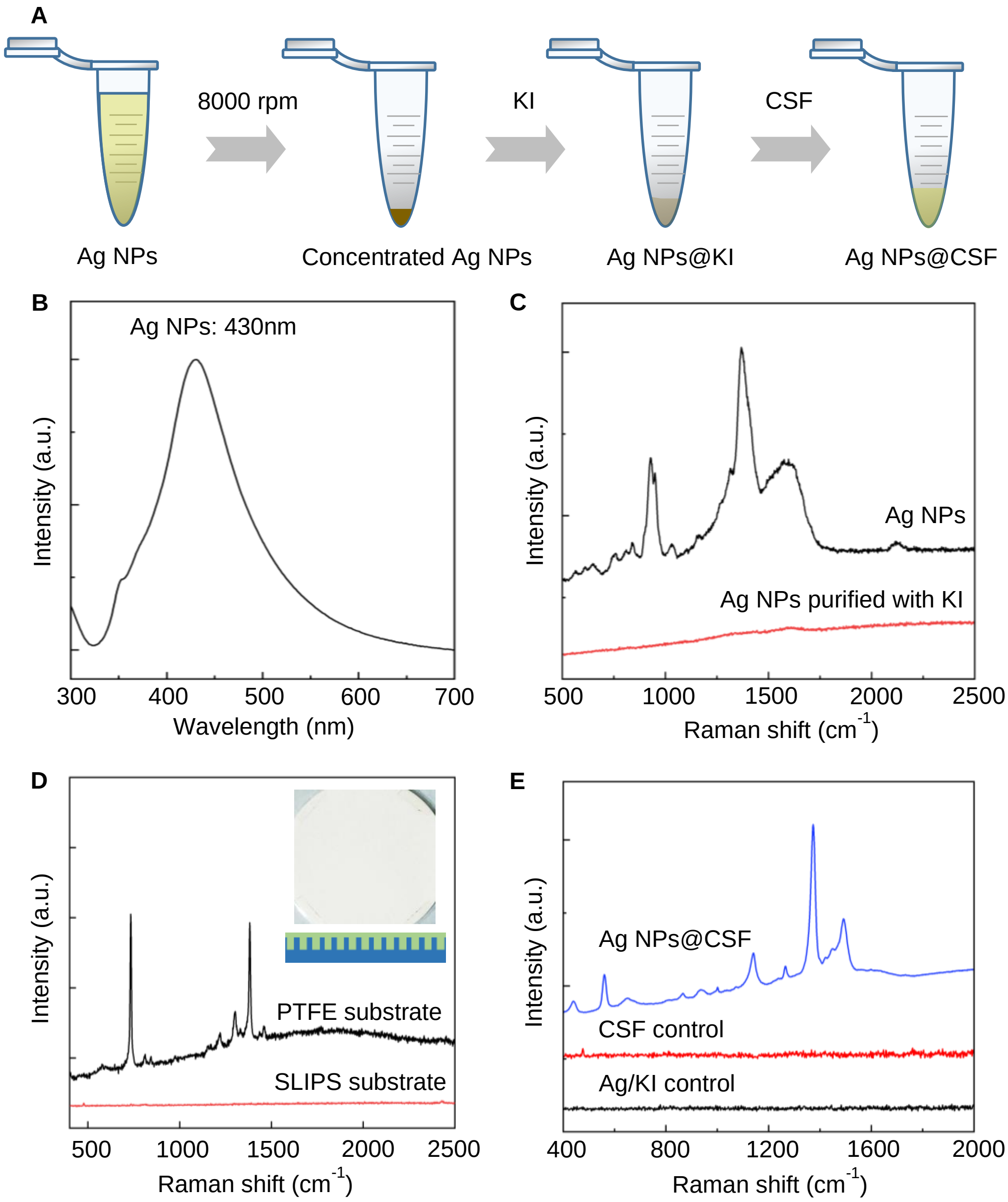

Figure S5. Characterizations of SERS enhanced substrates, related to Figure 2. (A) Schematic of sample preparation process. Firstly, concentrated Ag NPs with 50 nm size were purified with KI. Then, co-incubation process of Ag NPs and clinical CSF was taken for 10 minutes. **(B)** UV-vis absorption spectra of Ag NPs. **(C)** SERS spectra of Ag NPs before and after purified with KI agent. **(D)** SERS spectra of PTFE membrane and slippery liquid-infused porous surface (SLIPS) substrate. The insets show the optical image of SLIPS substrate. **(E)** SERS spectra of Ag/KI NPs control, CSF control (from AML patient), and mixture of Ag NPs and CSF.

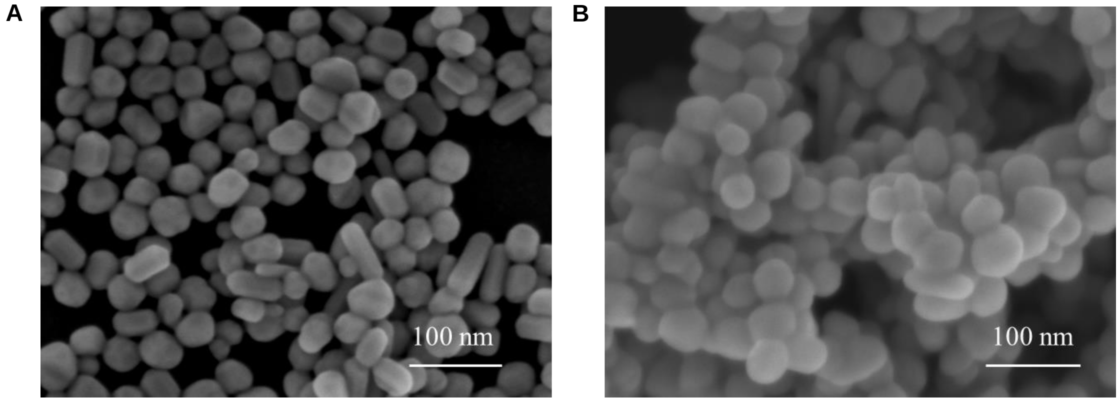

Figure S6. SEM images of colloidal Ag NPs with different aggregation station, related to Figure 2. (A) Ag NPs purified with KI. **(B)** Ag NPs mixed with CSF sample.

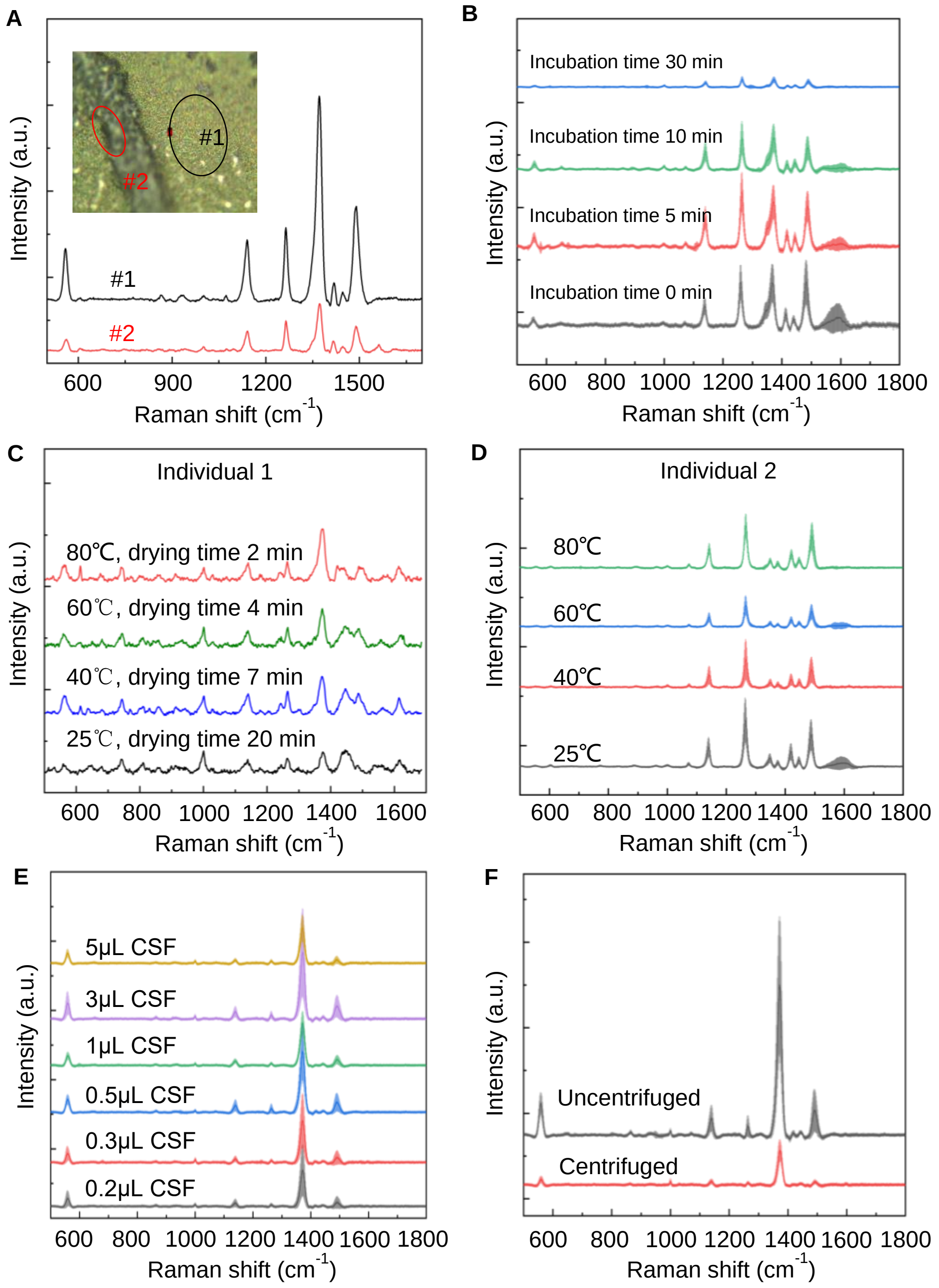

Figure S7. Optimization of SERS detection process for cerebrospinal fluid (CSF) samples, related to Figure 2. (A) Compared SERS spectra of CSF from ALL patient. Inset shows the optical image of dried SERS substrates. **(B)** SERS spectra of CSF sample with different incubation time (0, 5, 10, and 30 mins) between colloidal silver nanoparticles and CSF samples. **(C-D)** SERS spectra of CSF samples from various individuals with different evaporation temperatures. **(E)** SERS spectra of CSF samples with different volumes. **(F)** SERS spectra of CSF samples before and after the pretreatment of centrifugation.

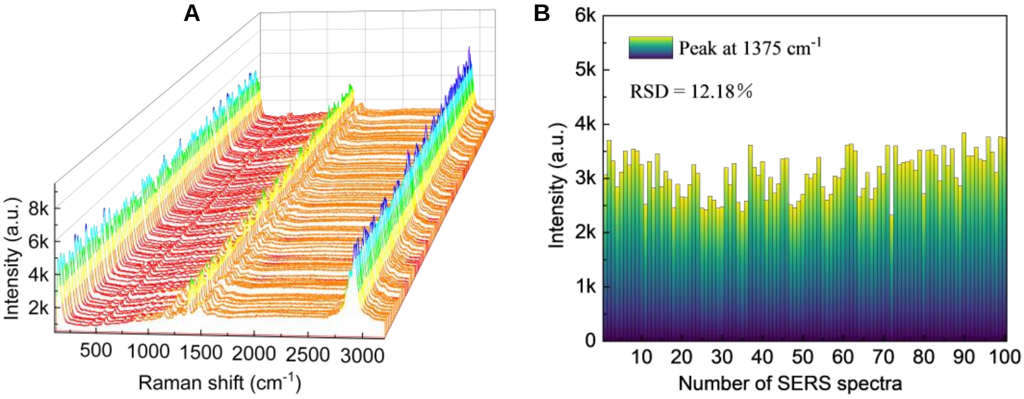

Figure S8. The performance of SERS consistency, related to Figure 2. (A) 50 of SERS spectra. **(B)** the relative standard deviation (RSD) value of characteristic band at 1375 cm^{-1}.

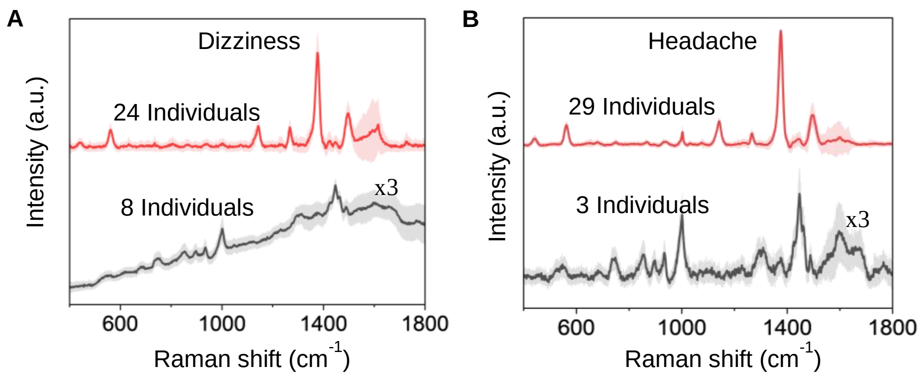

Figure S9. The compared SERS spectra of CSF samples from dizziness and headache individuals, related to Figure 2. (A) 32 individuals with dizziness. **(B)** 32 individuals with headache.

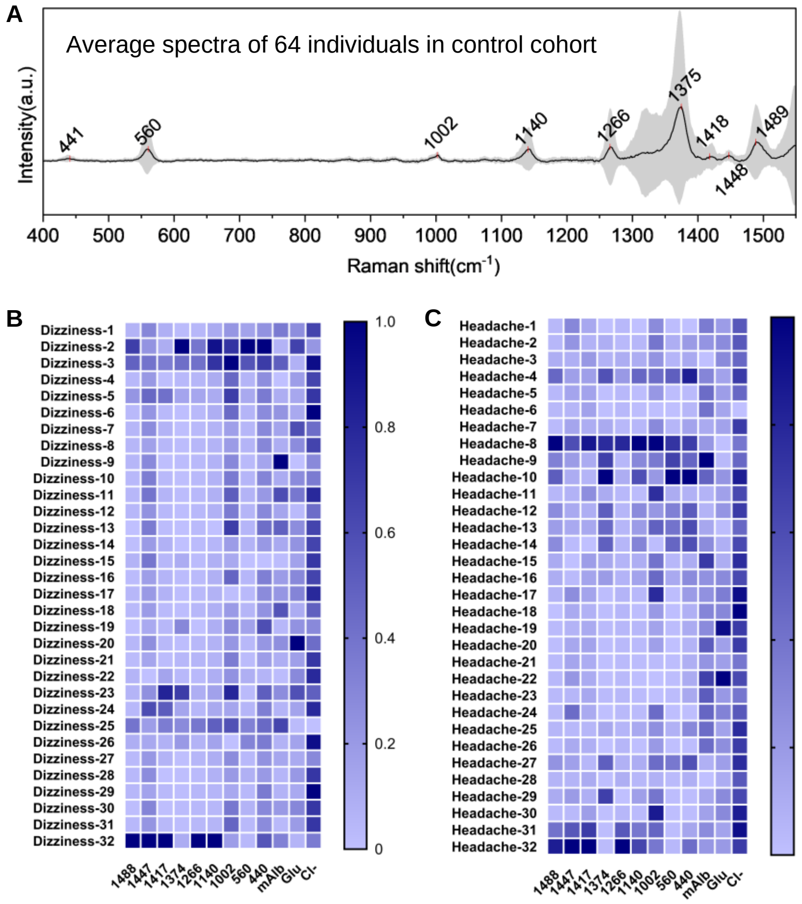

Figure S10. The individual variability within the healthy group, related to Figure 2. (A) The average SERS spectra of CSFs collected from healthy group. **(B)** The heat mappings of SERS characteristic peaks and chemical components to visualize the inter-individual variability and signal uniformity.

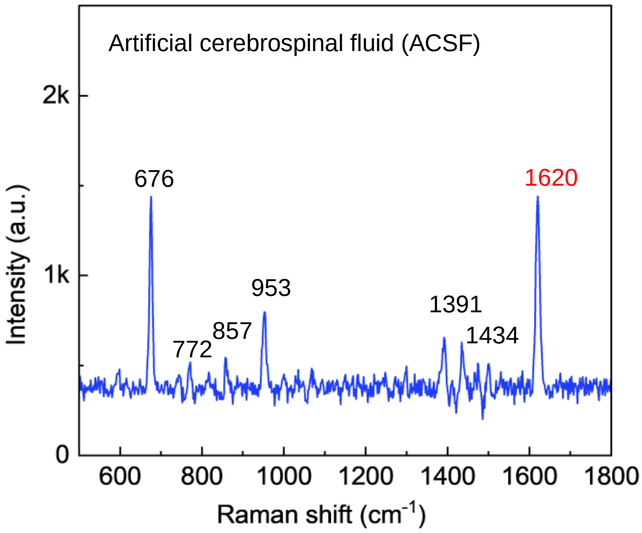

Figure S11. Typical SERS spectra of artificial cerebrospinal fluid (ACSF), related to Figure 2.

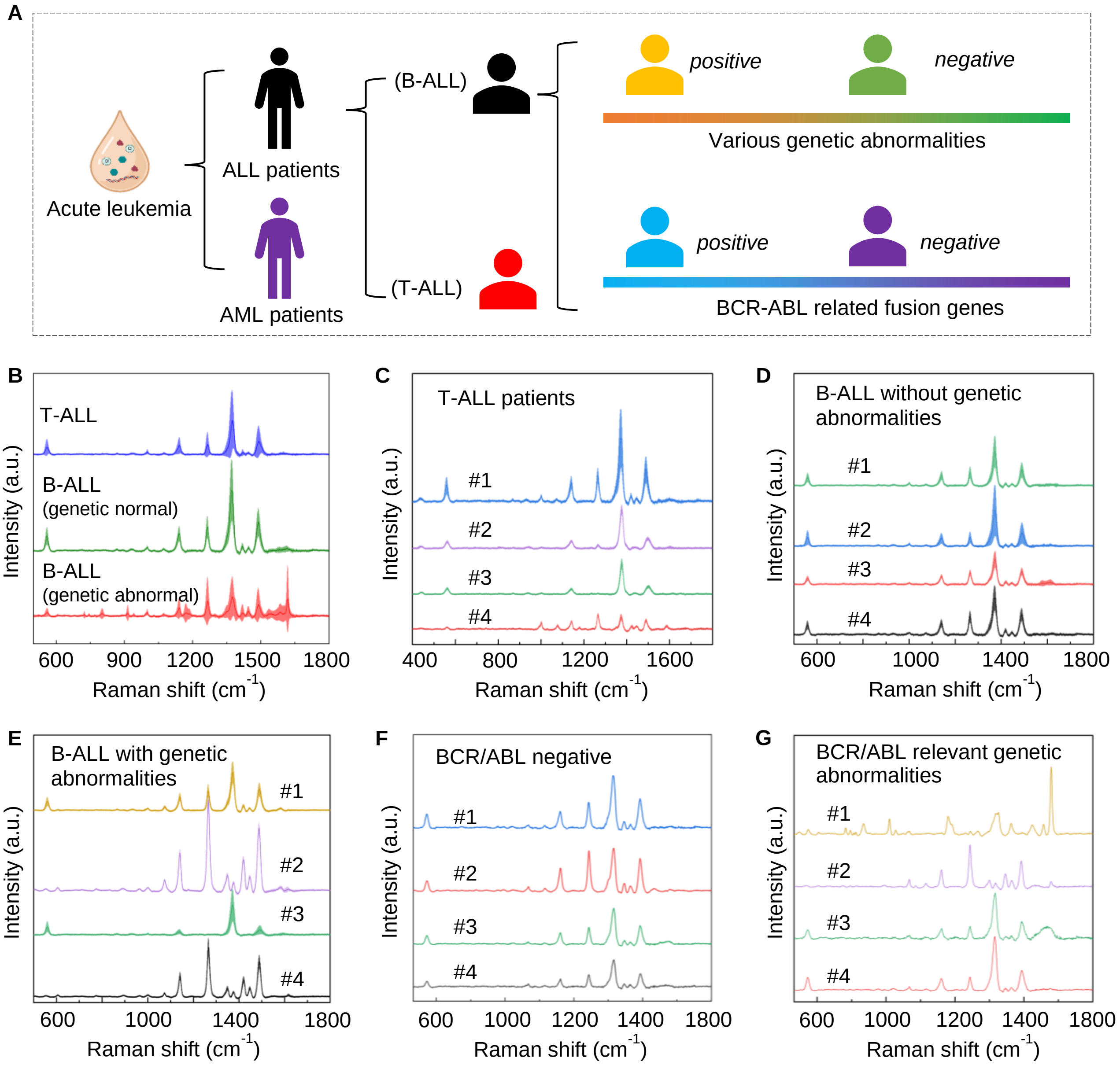

Figure S12. SERS analysis of CSF samples from various acute leukemia (AL) patients, related to Figure 3. (A) Overview of the AL patient information, i.e., AML/ALL, sub-categories of AML and ALL, and B-ALL with genetic or BCR/ABL relevant abnormalities. **(B)** SERS spectra of CSF samples collected from T-ALL and B-ALL patients. **(C-G)** Influence of individual differences on SERS spectra of CSF from ALL patients: **(C)** T-ALL patients; **(D)** B-ALL without genetic abnormalities; **(E)** B-ALL with different genetic abnormalities; **(F)** BCR/ABL negative B-ALL patients, and **(G)** B-ALL with different BCR/ABL relevant genetic abnormalities. For each CSF sample, at least 50 of SERS spectra was collected.

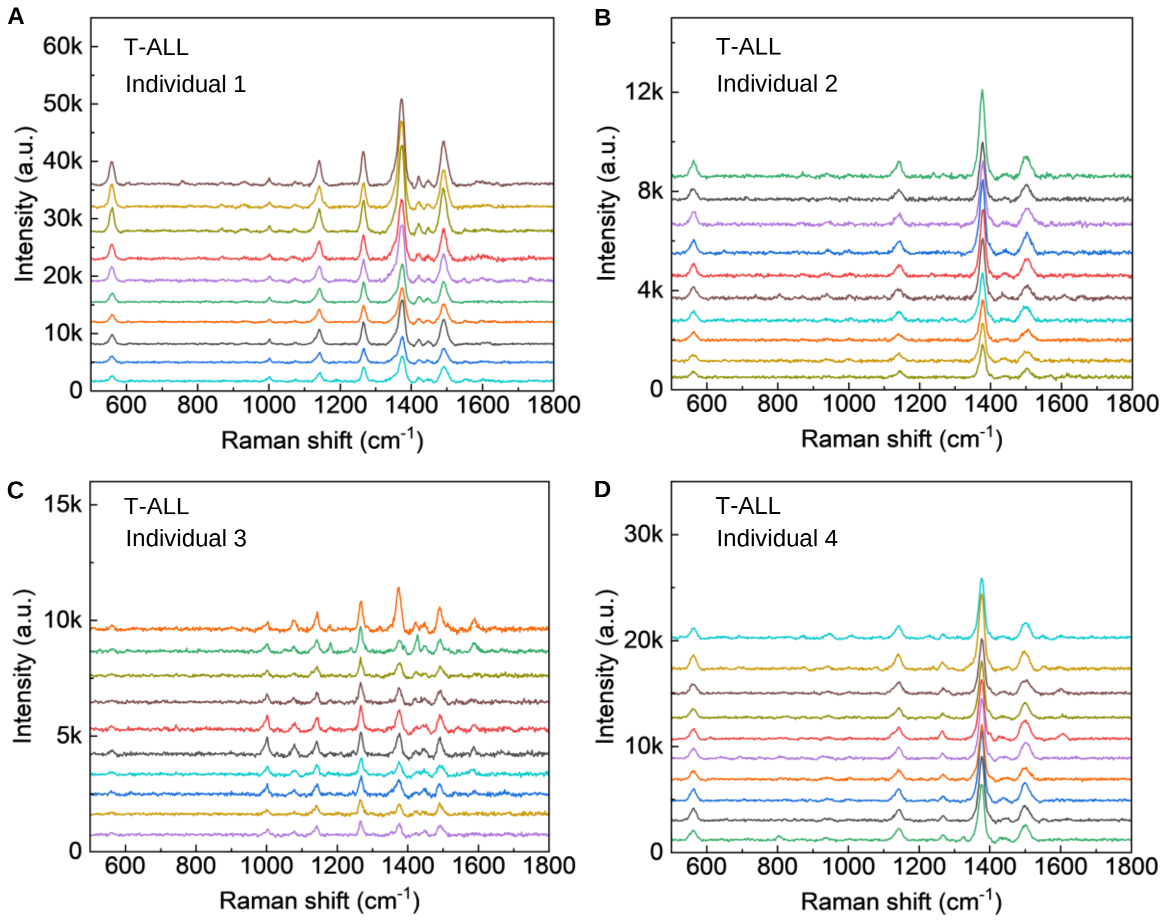

Figure S13. SERS spectra of CSF samples from different T-ALL patients, related to Figure 3.

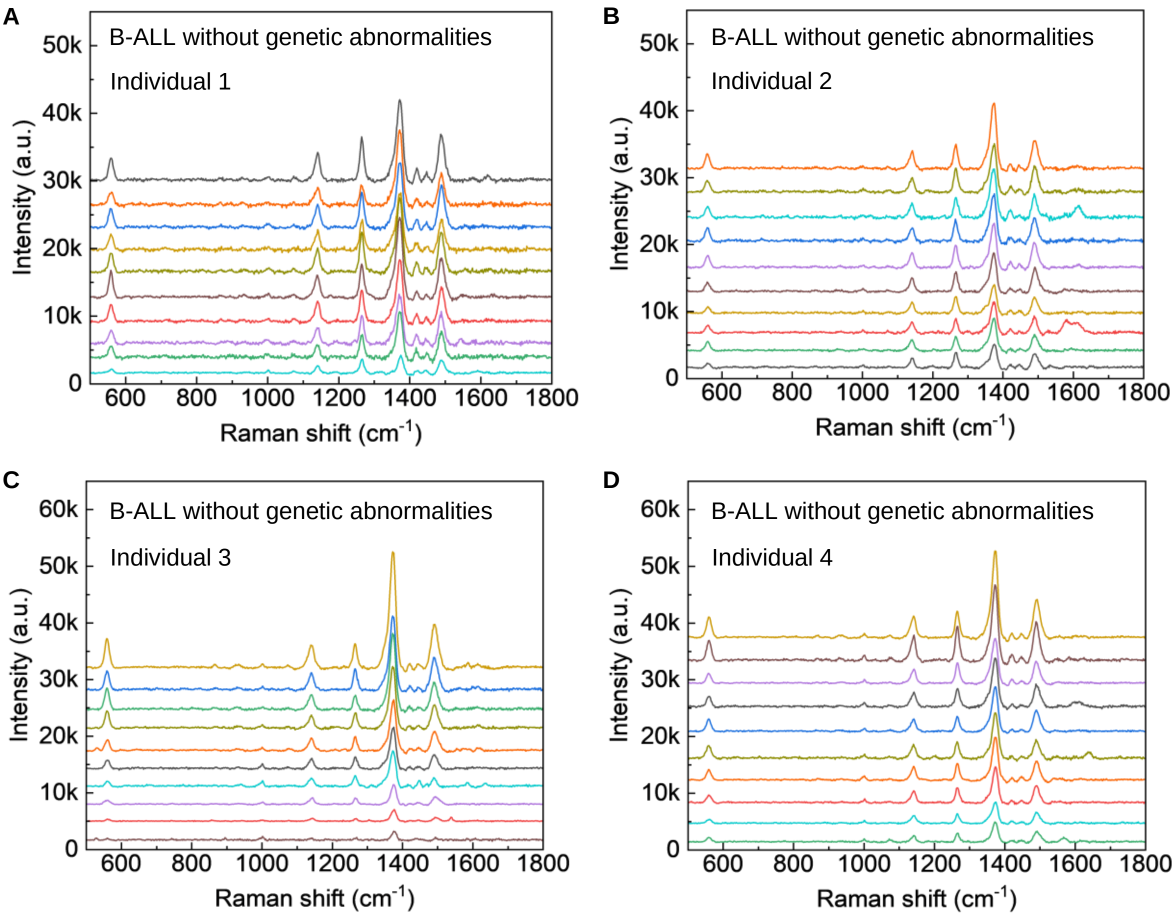

Figure S14. SERS spectra of CSF from B-ALL patients without genetic abnormalities, related to Figure 3.

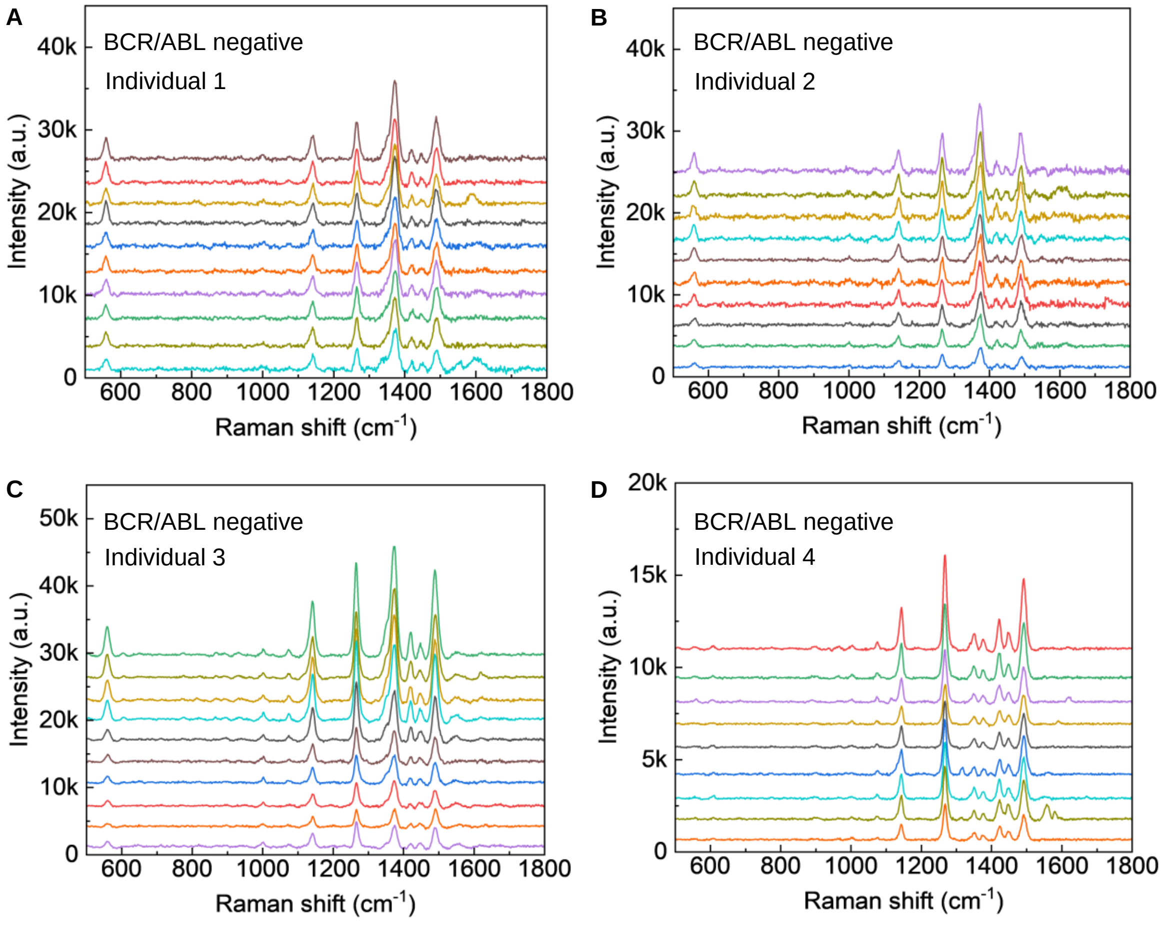

Figure S15. SERS spectra of CSF from different BCR/ABL negative ALL patients, related to Figure 3.

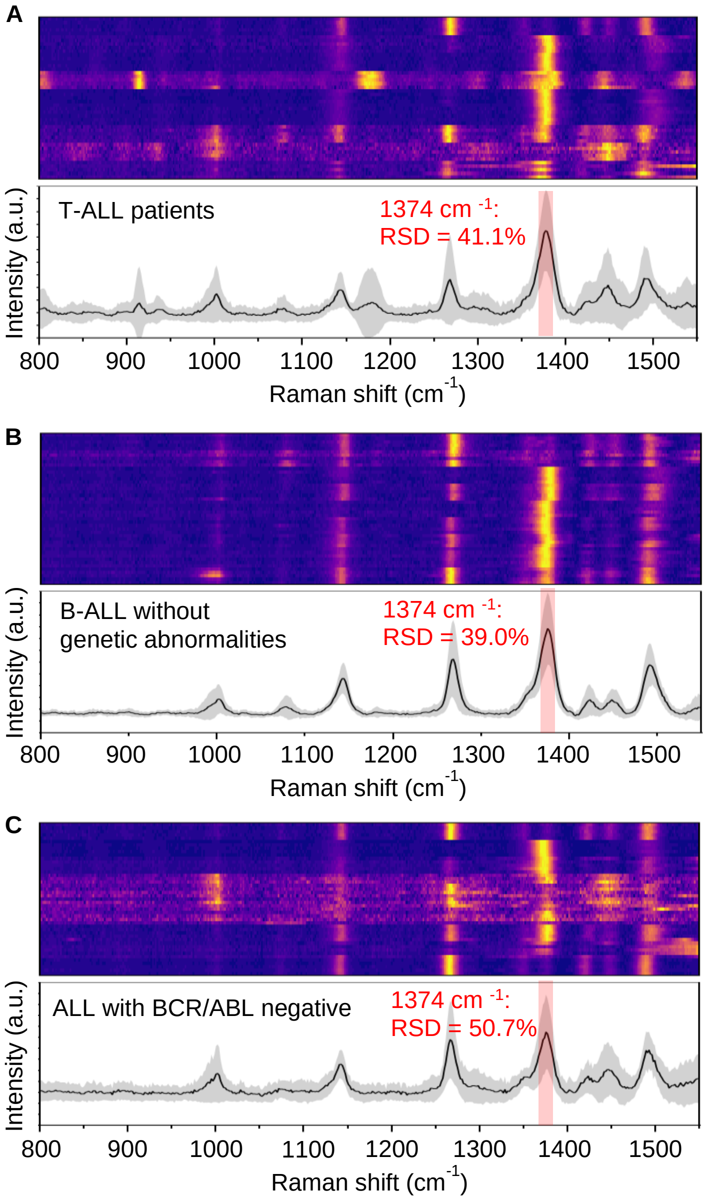

Figure S16. The average SERS spectra and corresponding heat mapping to visualize the inter-individual variability and signal uniformity, related to Figure 3. (A) SERS spectra and heat mapping of CSFs from T-ALL patients; **(B)** SERS spectra and heat mapping of CSFs from B-ALL without genetic abnormalities; **(C)** SERS spectra and heat mapping of CSFs from ALL with BCR/ABL negative.

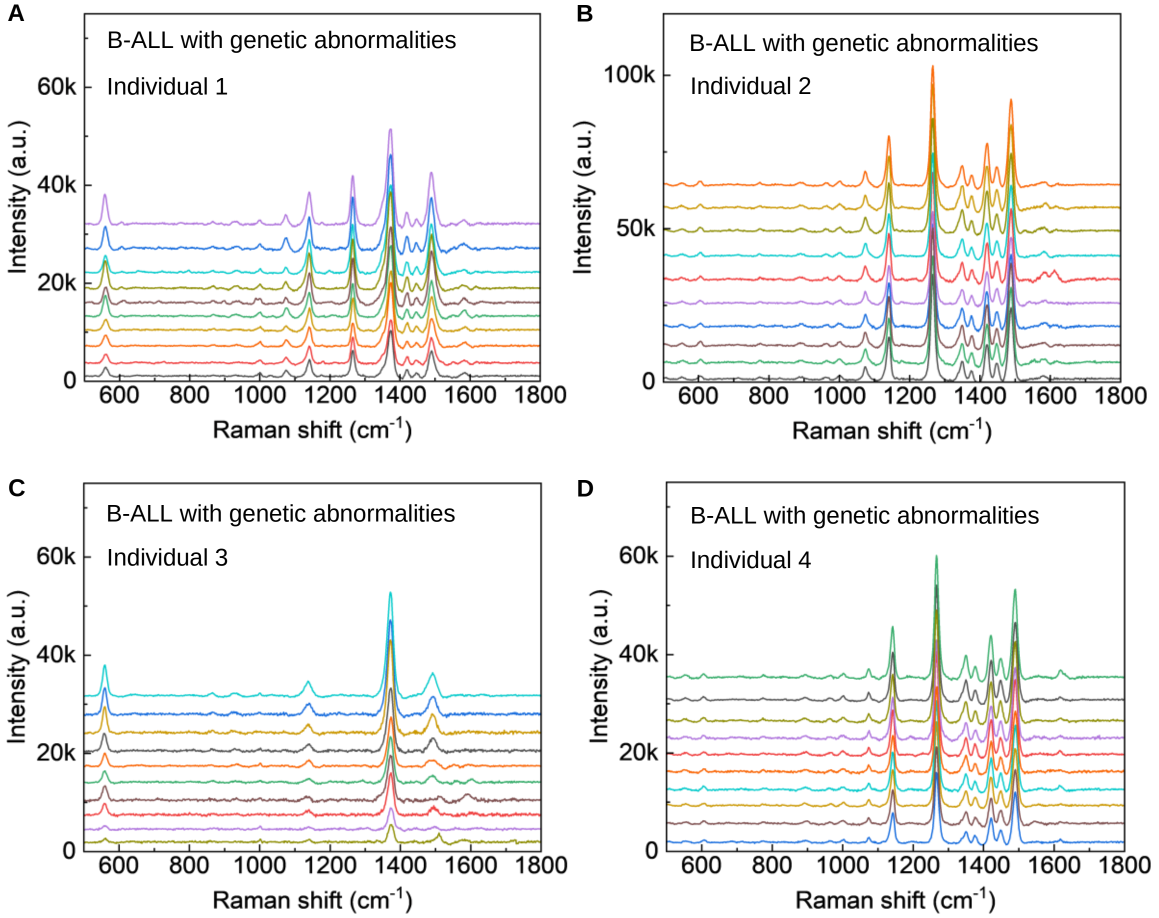

Figure S17. SERS spectra of CSFs from different B-ALL patients with genetic abnormalities, related to Figure 3.

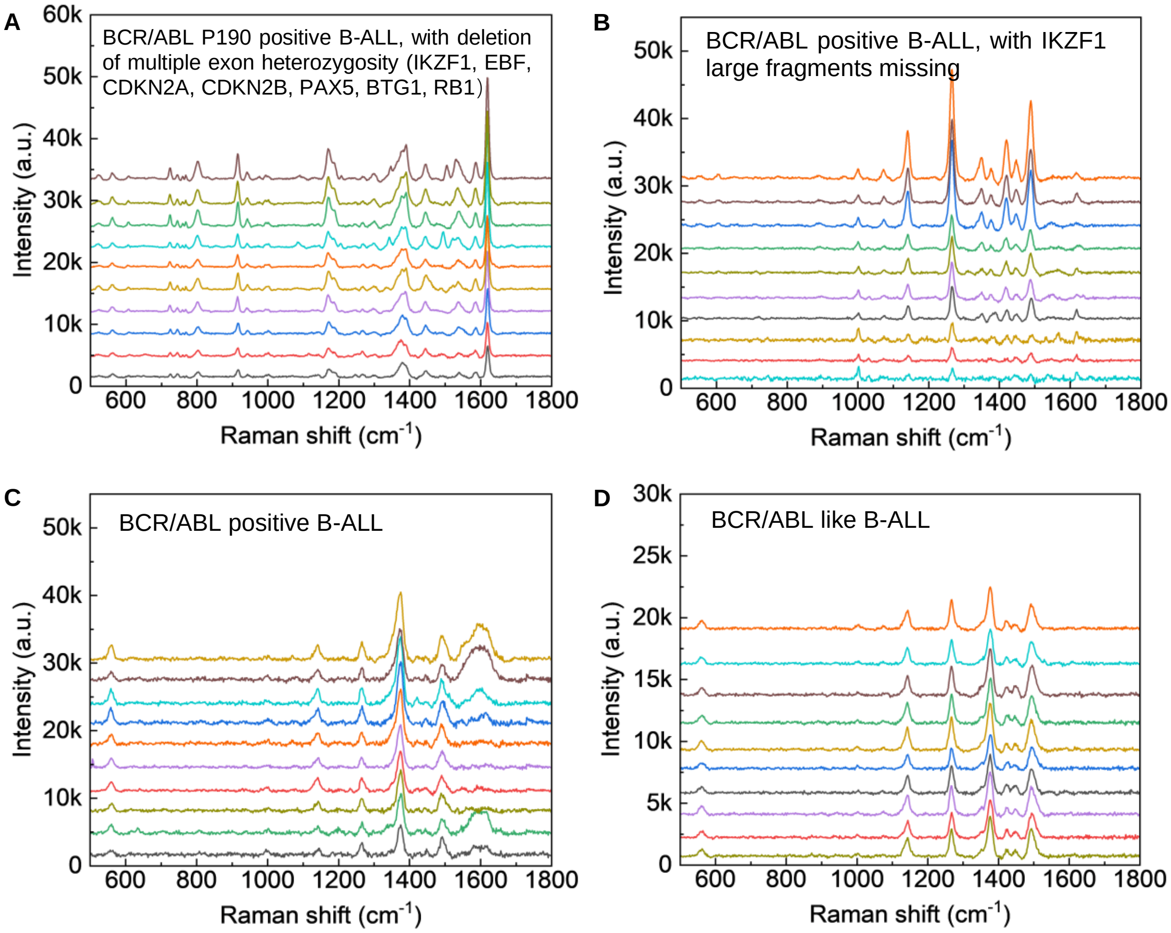

Figure S18. SERS spectra of different CSF samples from B-ALL patients with BCR/ABL relevant genetic abnormalities, related to Figure 3.

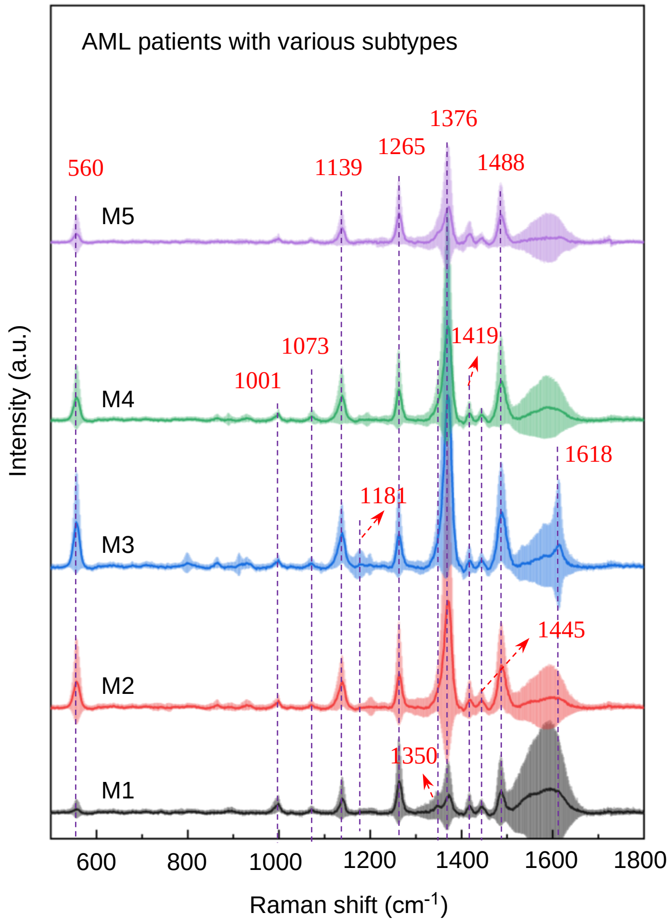

Figure S19. Compared SERS spectra of CSF from AML patients with different subtypes, related to Figure 3.

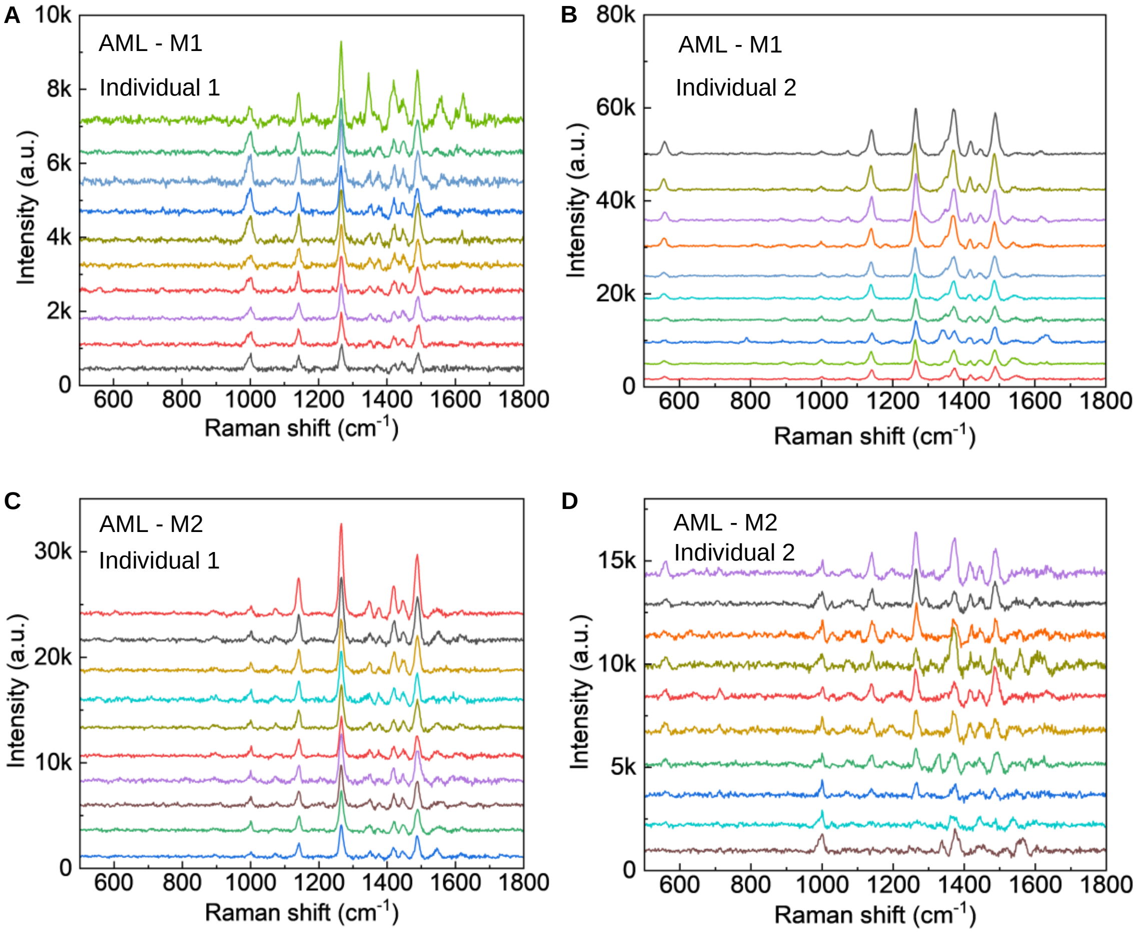

Figure S20. SERS spectra of CSF samples from acute myeloid leukemia (AML), related to Figure 3. (A-B) M1 subtype. **(C-D)** M2 subtype.

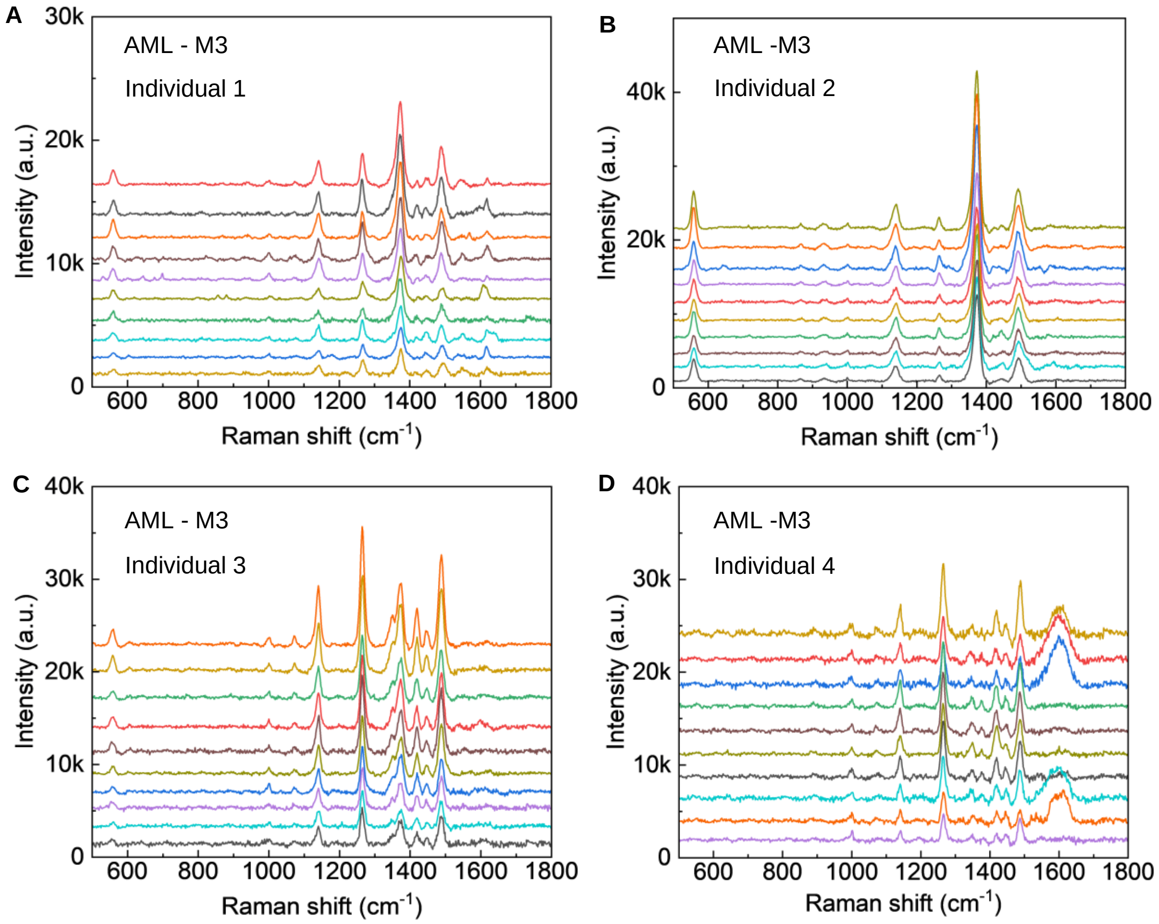

Figure S21. SERS spectra of CSF samples from different AML patients with M3 subtype, related to Figure 3.

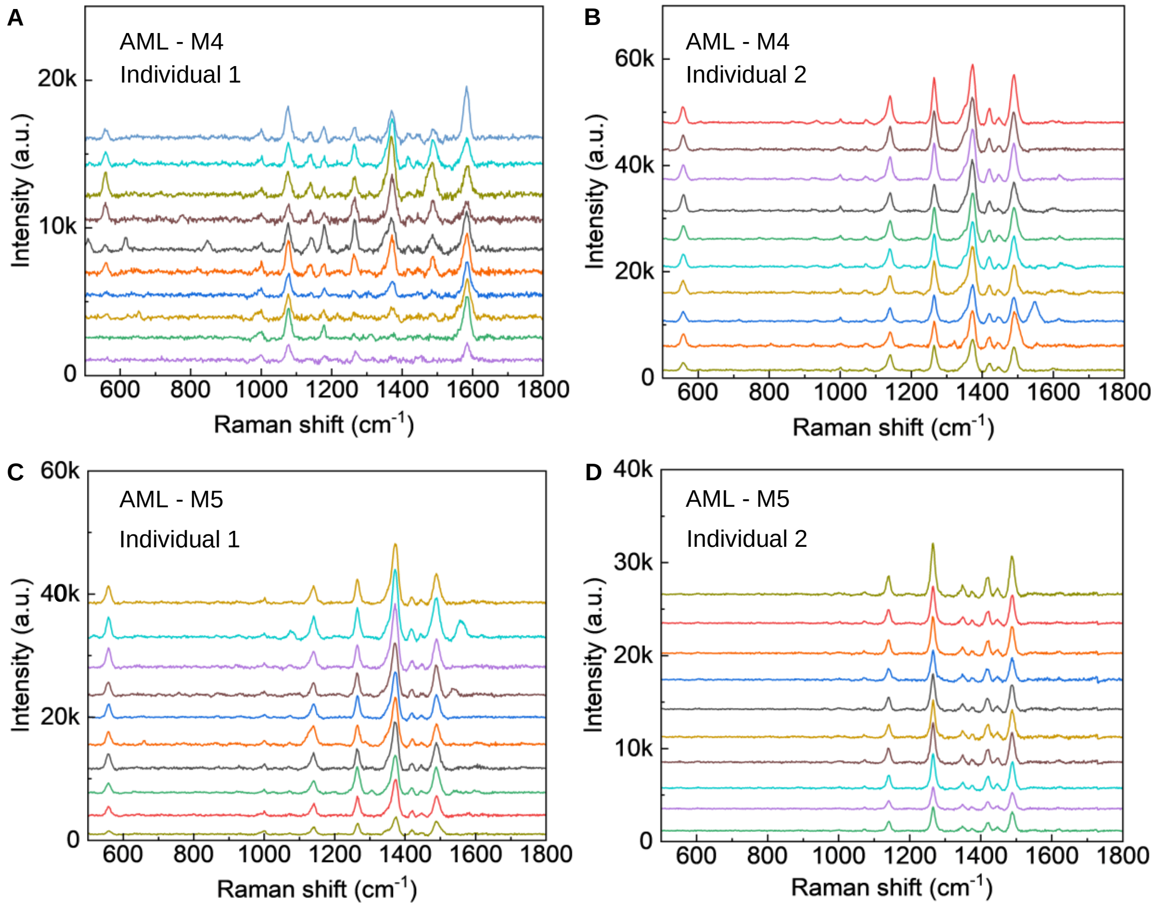

Figure S22. SERS spectra of CSF from different AML patients, related to Figure 3. (A-B) M4 subtype. **(C-D)** M5 subtype.

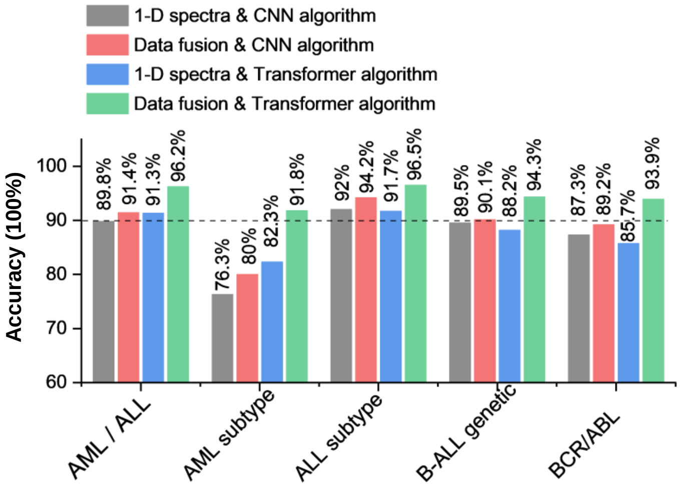

Figure S23. Compared prediction accuracy performances based on various DL models, related to Figure 3. The models include features fusions (*1-D* spectra and *2-D* image) combined with transformer, transformer based on *1-D* spectra, features fusion combined with CNN, and CNN based on *1-D spectra*.

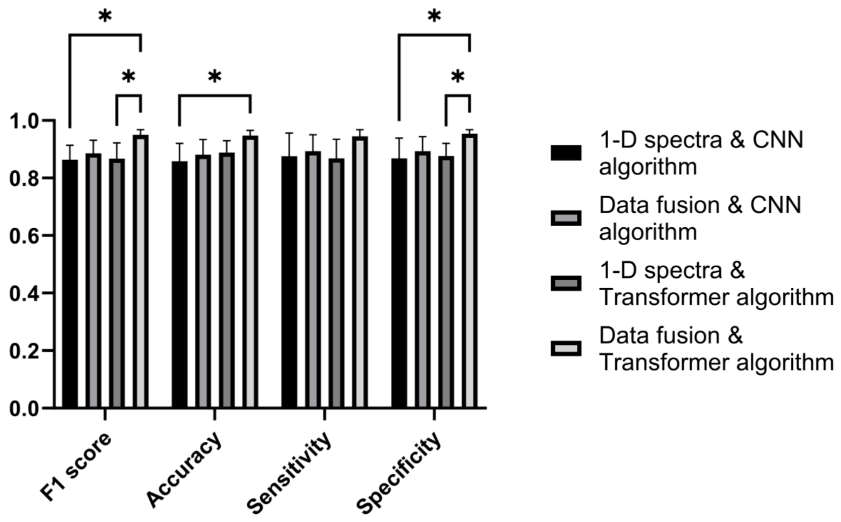

Figure S24. The significance test on the classification performance metrics obtained from different methods, related to Figure 3. The classification indexes include F1 score, accuracy, sensitivity, and specificity.

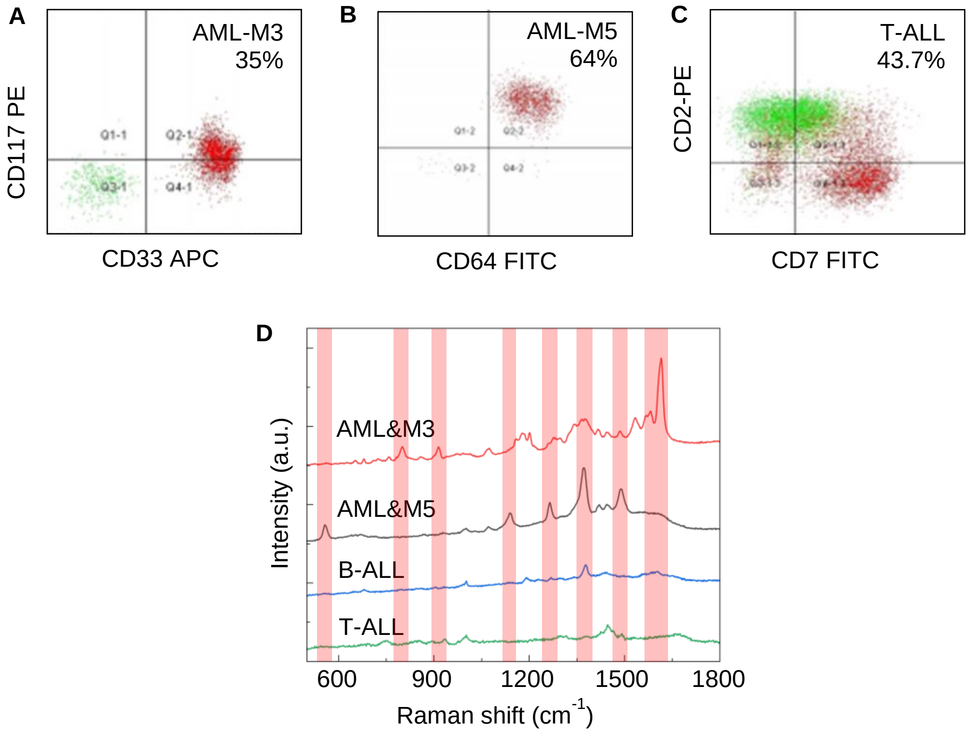

Figure S25. Flow cytometry (FCM) and related SERS analysis of different acute leukemia patients with central nervous system (CNS) involvements, related to Figure 4. (A-C) FCM results of patients with CNS leukemia (CNSL) that developed from **(A)** acute myeloid leukemia with M3 subtype (AML&M3), **(B)** acute myeloid leukemia with M5 subtype (AML&M5), and **(C)** T-cell acute lymphoblastic leukemia (T-ALL). In these figures, the red dots denote leukemia cells, while the green dots signify normal lymphocytes. **(D)** SERS spectra of CSF samples from CNSL patients developed from diverse acute leukemia (AL) subtypes, encompassing AML&M3, AML&M5, B-ALL, and T-ALL.

Table S1. The components of artificial cerebrospinal fluid (ACSF), related to Figure 2.

Components	NaCl	KCI	$NaHCO_3$	NaH_2PO_4	$MgSO_4$	$CaCl_2$	$C_6H_{12}O_6$	H_2O
Volume (μL)	100	100	100	10	10	10	10	660
Concentration (mM)	1200	30	260	124	200	200	100	/

Table S2. Information of CSF samples from healthy and acute leukemia groups, related to Figure 2.

			Healthy control cohort		Acute leukemia cohort				
					AML patients			ALL patients	
			Dizziness (32)	Headaches (32)	M1&M2 (26)	M3 (22)	M4&M5 (33)	B-ALL (52)	T-ALL (15)
Exterior features of sample	Transparency	Yes	31	32	25	22	32	52	15
		No	1	0	1	0	1	0	0
	Clot	Yes	0	0	0	0	0	0	0
		No	32	32	26	22	33	52	15
Main biochemical components	Total protein (g/L)	0-0.3	1	11	11	5	6	18	3
		0.3-0.6	18	20	12	13	22	27	10
		＞0.6	13	1	3	4	5	7	2
		Mean value (90% range)	0.64±0.12 (0.30-1.83)	0.36±0.05 (0.17-0.79)	0.41±0.11 (0.15-1.42)	0.41±0.07 (0.18-0.86)	0.43±0.06 (0.12-1.18)	0.39±0.05 (0.21-1.15)	0.48±0.12 (0.25-1.17)
	Chloride (mmol/L)	＜120	2	1	2	0	0	1	0
		120-130	28	28	24	20	30	46	13
		＞130	2	3	0	2	3	5	2
		Mean value (90% range)	124.40±1.12 (116-130)	126.09±0.89 (118-131)	125.21±1.01 (119-130)	127.41±1.20 (123-135)	126.25±1.07 (121-133)	126.40±0.82 (118-134)	124.93±1.90 (120-133)
	Glucose (mmol/L)	2-3	5	3	7	3	10	14	4
		3-4	22	25	17	19	22	32	6
		＞4	5	4	2	0	1	6	5
		Mean value (90% range)	3.62±0.30 (2.61-6.72)	3.71±0.34 (2.61-7.30)	3.43±0.25 (2.50-5.48)	3.27±0.11 (2.69-3.91)	3.20±0.11 (2.70-4.26)	3.37±0.15 (2.18-4.87)	3.71±0.44 (2.57-5.31)
Number of cells containing in CSF	0-10		27	23	9	12	17	21	4
	10-50		2	1	0	1	0	0	0
	＞50		3	8	7	4	6	3	2

Table S3. Peak assignments of the potential biochemical components in CSF, related to Figure 2, Figure 4, and Figure 5.

Peaks (cm^{-1})	Vibrational mode	Possible assignments	Ref.
560	/	adenine, glycine	1
723	C-H bending	nucleic acid, adenine, coenzyme A;	2, 3, 4
744	Ring breathing vibrations	nucleic acid, thymine	2
803	C-C-O stretching vibration	glutathione, L-serine, thymine	3, 4, 5, 6
914	Ring breathing vibrations	glucose, phenylalanine	7
1001	C-C stretching; C−C ring breathing	amino acid; phenylalanine, lactose	2, 3, 4, 7
1028	C-H in plane deformation	phenylalanine	6, 8
1073	C-N stretching, skeletal C-C stretching	protein; collagen; L-glutamate,	2, 3, 4
1139	C-H stretching; C-C stretching; C-N stretching	nucleic acid; adenine	2, 3, 4
1169	C–N stretching	nucleic acid	2, 7
1181	C-C symmetric stretching; C-O symmetric stretching	cytosine; adenine; guanine	8
1265	C−H deformation; C-N stretching	nucleic acid	8
1350	C-N stretching	proline	8
1373	Ring breathing	thymine, adenine, guanine	5, 8
1376	COO^- symmetric stretching	nucleic acids	7, 9
1419	C=C stretching	DNA, RNA	8
1445	- CH_2 deformation; $\delta(CH_2)$	malic acid; L-phenylalanine	3, 5, 7
1488	C-H bending vibrations	proteins, collagen	7, 9
1537	Amide carbonyl group vibrations, aromatic hydrogens	L-Glutamate, glutathione	8
1583	C=C bending; ring stretch	nucleic acids; tryptophan	5, 7, 9
1618	C=C in-plane bending	tyrosine; amino acid	2, 4, 6

Table S4. Characteristics of the B-ALL patients with BCR/ABL fusion genetic abnormalities, related to Figure 3.

B-ALL	Age in years	Gender	Diagnosis	Other genetic abnormalities
Individual-1	32	Male	BCR/ABL (Ph+)	None
Individual-2	34	Female	BCR/ABL (Ph+), p190	Heterozygous deletions of multiple gene exons in IKZF1, EBF, CDKN2A, CDKN2B, PAX5, BTG1, RB1.
Individual-3	67	Female	BCR/ABL (Ph+)	T315I gene mutation
Individual-4	44	Male	BCR/ABL (Ph+), p210	Large fragment deletions of IKZF1, CDKN2A, CDKN2B, ETV6 and PAX5, accompanied by ABL kinase T315I mutation
Individual-5	31	Female	Ph-like ALL	None
Individual-6	28	Male	Ph-like ALL	Large-fragment deletion mutation in IKZF1 gene.
Individual-7	34	Female	BCR/ABL (Ph+), p190	Heterozygous deletions of multiple gene exons in IKZF1, EBF, CDKN2A, CDKN2B, PAX5, BTG1, RB1.
Individual-8	32	Female	Ph-like ALL	None
Individual-9	65	Male	Ph-like ALL	None
Individual-10	34	Female	BCR/ABL (Ph+)	Heterozygous deletions in exons 1 to 8 of IKZF1 gene.
Individual-11	32	Female	Ph-like ALL	None
Individual-12	32	Male	BCR/ABL (Ph+)	None
Individual-13	32	Male	BCR/ABL (Ph+)	None
Individual-14	36	Female	BCR/ABL (Ph+)	Large fragment deletions of IKZF1 gene.
Individual-15	32	Male	BCR/ABL (Ph+), p210	Heterozygous deletions in exons 1-8 of IKZF1 gene, exons 2 and 4 of CDKN2A gene, exon 2 of CDKN2B gene, and exons 1, 2, 5, 6, 7, 8, and 10 of PAX5 gene.
Individual-16	34	Female	BCR/ABL (Ph+), p190	Heterozygous deletions in exons 1-8 of IKZF1 gene, exons 2 and 4 of CDKN2A gene, exon 2 of CDKN2B gene, and exons 1, 2, 5, 6, 7, 8, and 10 of PAX5 gene.
Individual-17	26	Female	BCR/ABL (Ph+)	46, XX, t (9,22)
Individual-18	21	Male	BCR/ABL (Ph+)	IKZF1、FAT1、FAT3 genetic positive, and heterozygous deletions in exons 4 to 7 of IKZF1 gene.
Individual-19	44	Female	BCR/ABL (Ph+)	None
Individual-20	33	Female	BCR/ABL (Ph+), p190	Heterozygous deletions in exons 4 to 7 of IKZF1 gene.
Individual-21	58	Male	BCR/ABL (Ph+), p190	t (9;22) (q34;q11), t (8;14) (q11;q32)
Individual-22	56	Female	BCR/ABL (Ph+)	45,XX,der(4)t(1;4)(q21;q35),t(9;22)(q34;q11),add(18)(p11.3)； ABL kinase T315I mutation, and heterozygous deletions in exons 4 to 7 of IKZF1 gene.
Individual-23	55	Male	BCR/ABL (Ph+)	t(9;22) (q34;q11), +X, del(2)(q23), add(4)(p16); Heterozygous deletions inexons of IKZF1, CDKN2A, BTG1, RB1 gene.
Individual-24	19	Male	Ph-like ALL	PDGFRB、CSF1R gene rearrangement; heterozygous deletions in exons 4-8 of IKZF1 gene, exons 2, 5, 6, and 7 of PAX5 gene, and exon 16 of EBF1 gene.
Individual-25	22	Female	Ph-like ALL	SSBP2/CSF1R fusion genetic positive; heterozygous deletions of multiple exons of IKZF1 gene
Individual-26	55	Male	BCR/ABL (Ph+)	t(9;22) (q34;q11), +X, del(2)(q23), add(4)(p16); heterozygous deletions in exons 4-7 of IKZF1 and CDKN2A gene; exons 6, 14, 19, and 26 of RB1 gene; and allelic loss in exon 2 of BTG1 gene.

Table S5. Prediction performance for external set validation, related to Figure 4.

AML/ALL (Binary classification)				AML subtypes (Trinary classification)						ALL subtypes (Binary classification)				B-ALL genetic (Binary classification)			
AML (n=15)		ALL (n=15)		M1/M2 (n=10)		M3 (n=10)		M4/M5 (n=10)		B-ALL (n=15)		T-ALL (n=15)		Normal (n=15)		Abnormal (n=15)	
No.	Feedback	No.	Feedback	No.	Feedback	No.	Feedback	No.	Feedback	No.	Feedback	No.	Feedback	No.	Feedback	No.	Feedback
#1	√	#16	√	#31	√	#41	√	#51	√	#61	√	#76	√	#91	√	#106	√
#2	×	#17	√	#32	√	#42	√	#52	√	#62	√	#77	√	#92	√	#107	√
#3	√	#18	√	#33	×	#43	√	#53	√	#63	√	#78	×	#93	√	#108	√
#4	√	#19	√	#34	√	#44	√	#54	√	#64	√	#79	√	#94	√	#109	√
#5	√	#20	×	#35	×	#45	×	#55	√	#65	√	#80	√	#95	√	#110	√
#6	√	#21	√	#36	√	#46	√	#56	√	#66	√	#81	√	#96	√	#111	√
#7	√	#22	√	#37	√	#47	√	#57	√	#67	√	#82	√	#97	√	#112	√
#8	√	#23	√	#38	√	#48	√	#58	√	#68	√	#83	√	#98	√	#113	√
#9	√	#24	√	#39	√	#49	√	#59	√	#69	√	#84	√	#99	√	#114	√
#10	√	#25	√	#40	√	#50	√	#60	√	#70	√	#85	√	#100	×	#115	√
#11	√	#26	√							#71	√	#86	√	#101	√	#116	√
#12	√	#27	√							#72	√	#87	√	#102	√	#117	×
#13	√	#28	√							#73	√	#88	√	#103	√	#118	√
#14	√	#29	√							#74	√	#89	√	#104	√	#119	√
#15	√	#30	√							#75	√	#90	√	#105	√	#120	√

Table S6. Characteristics of the test datasets for various meningitis patients, related to Figure 5.

	No. of subjects	No. of SERS spectra	Gender		Age in years (range)
			Male	Female	
Viral Meningitis	15	750	10(67%)	5(33%)	38±8(17-78)
Tubercular Meningitis	15	750	8(53%)	7(47%)	44±8(24-75)
Cryptococcal Meningitis	15	750	13(86%)	2(14%)	45±7(15-66)
Autoimmune Encephalitis	15	750	8(53%)	7(47%)	36±8(16-72)

Supplementary References

1. Sathyavathi, R. et al. Raman spectroscopy provides a powerful, rapid diagnostic tool for the detection of tuberculous meningitis in ex vivo cerebrospinal fluid samples. *J Biophotonics* **6**, 567-572 (2013).

2. Ye, M. et al. Subtype discrimination of acute myeloid leukemia based on plasma SERS technique. *Spectrochim Acta. Part A: Molecular and biomolecular spectroscopy* **271**, 120865 (2022).

3. Chen, S. et al. Predicting prognosis in acute myeloid leukemia patients by surface-enhanced Raman spectroscopy. *Nanomedicine* **16**, 1873-1885 (2021).

4. Chen, Y. et al. Optical tweezers and Raman spectroscopy for single-cell classification of drug resistance in acute lymphoblastic leukemia. *Journal of Biophotonics* **15**, e202200117 (2022).

5. Parlatan, U. et al. Label-Free Identification of Exosomes using Raman Spectroscopy and Machine Learning. *Small* **19**, e2205519 (2023).

6. Yu, Y. et al. Leukemia cells detection based on electroporation assisted surface-enhanced Raman scattering. *Biomed Opt Express* **8**, 4108-4121 (2017).

7. Maria Nowakowska, A. et al. Reliable cell preparation protocol for Raman imaging to effectively differentiate normal leukocytes and leukemic blasts. *Spectrochim Acta A: Mol Biomol Spectrosc* **292**, 122408 (2023).

8. Movasaghi, Z. et al. Raman Spectroscopy of Biological Tissues. *Applied Spectroscopy Reviews* **42**, 493-541 (2007).

9. Leszczenko, P. et al. Towards Raman-Based Screening of Acute Lymphoblastic Leukemia-Type B (B-ALL) Subtypes. *Cancers (Basel)* **13**, 5483 (2021).
