# Supplementary material for: Rapid and sensitive acute leukemia classification and diagnosis platform using deep learning-assisted SERS detection
Source: Cell Rep Med. 2025 Sep 8;6(9):102320. doi: 10.1016/j.xcrm.2025.102320 (PMC12490261; doi:10.1016/j.xcrm.2025.102320)
Supplement: Document S2. Article plus supplemental information [file mmc2.pdf]

Cell Reports
Medicine

Article

Rapid and sensitive acute leukemia classification and diagnosis platform using deep learning-assisted SERS detection

Graphical abstract

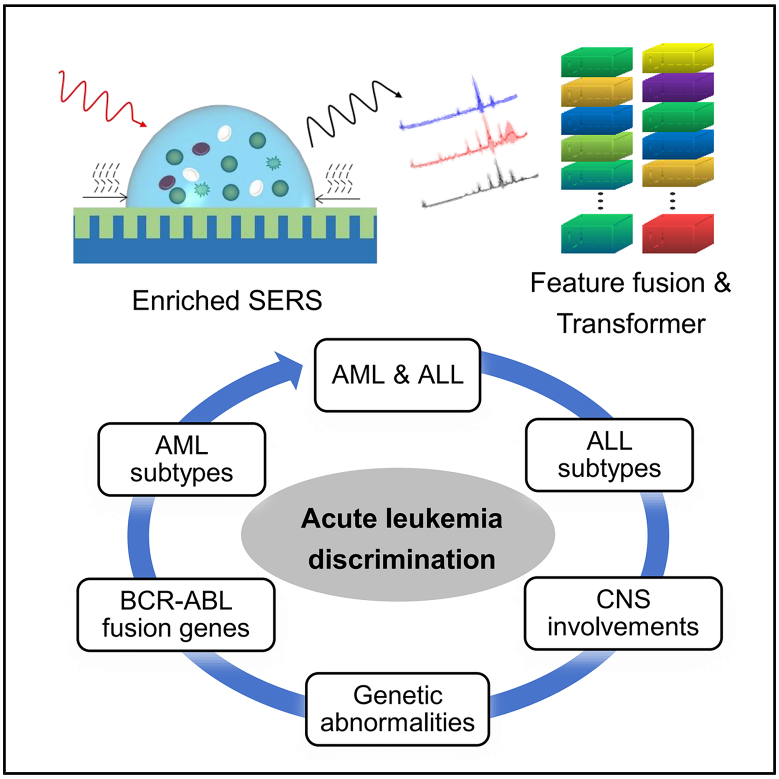

Highlights

- Enriched SERS is employed to detect clinical cerebrospinal fluids (CSFs)
- Sensitive spectral classification is established through transformer
- Discriminations of acute leukemia with various categories or subtypes are realized

Authors

Dongjie Zhang, Zhaoyang Cheng, Yali Song, ..., Qi Zeng, Tingting Zeng, Xueli Chen

Correspondence

chen-renan@163.com (R.C.),
sdqlyysnz@email.sdu.edu.cn (N.S.),
hanmin@sdfmu.edu.cn (M.H.),
qzeng@xidian.edu.cn (Q.Z.),
zengtingting80@scu.edu.cn (T.Z.),
xlchen@xidian.edu.cn (X.C.)

In brief

Zhang et al. propose a combined deep learning and surface-enhanced Raman spectroscopy (DL-SERS) classification strategy to achieve rapid and sensitive identifications of AL with various subtypes or genetic abnormalities. This DL-SERS classification platform has the potential to be a powerful auxiliary *in vitro* diagnostic tool.

Zhang et al., 2025, Cell Reports Medicine 6, 102320
September 16, 2025 © 2025 The Author(s). Published by Elsevier Inc.
https://doi.org/10.1016/j.xcrm.2025.102320

CellPress

Cell Reports Medicine

CellPress
OPEN ACCESS

Article

Rapid and sensitive acute leukemia classification and diagnosis platform using deep learning-assisted SERS detection

Dongjie Zhang,[1,2,3,4,16] Zhaoyang Cheng,[1,2,16] Yali Song,[5,16] Huandi Li,[1,2] Lin Shi,[1,2] Nan Wang,[1,2] Yingwen Peng,[1,2,3] Renan Chen,[6,*] Nianzheng Sun,[7,8,*] Min Han,[9,*] Fengjiao Hu,[10] Chuntao Zong,[6] Rui Zhang,[8] Si Chen,[5] Conghui Zhu,[6] Xiaoli Zhang,[6] Xiaobo Li,[11] Xiaopeng Ma,[12] Changbei Shi,[13] Xiaofei Zhang,[14] Rui Liu,[1,2] Ziqi Ren,[1,2] Lin Wang,[15] Qi Zeng,[1,2,*] Tingting Zeng,[5,10,*] and Xueli Chen[1,2,3,4,17,*]

[1]Center for Biomedical-photonics and Molecular Imaging, Advanced Diagnostic-Therapy Technology and Equipment Key Laboratory of Higher Education Institutions in Shaanxi Province, School of Life Science and Technology, Xidian University, Xi'an, Shaanxi 710126, China
[2]Engineering Research Center of Molecular and Neuro Imaging, Ministry of Education & Xi'an Key Laboratory of Intelligent Sensing and Regulation of Trans-Scale Life Information, School of Life Science and Technology, Xidian University, Xi'an, Shaanxi 710126, China
[3]Bi-optoelectronic-integration and Medical Instrumentation Laboratory, Guangzhou Institute of Technology, Xidian University, Guangzhou, Guangdong 510555, China
[4]State Key Laboratory of Electromechanical Integrated Manufacturing of High-Performance Electronic Equipment, Xidian University, Xi'an, Shaanxi 710071, China
[5]Department of Laboratory Medicine, West China Hospital, Sichuan University, Chengdu, Sichuan 610041, China
[6]Department of Hematology, Xi'an Daxing Hospital affiliated to Yan'an University, Xi 'an, Shaanxi 710082, China
[7]Department of Pediatrics, Qilu Hospital of Shandong University, Jinan, Shandong 250012, China
[8]Department of Hematology, Qilu Hospital of Shandong University Dezhou Hospital, Dezhou, Shandong 253000, China
[9]Department of Neurosurgery, The First Affiliated Hospital of Shandong First Medical University & Shandong Provincial Qianfoshan Hospital, Jinan, Shandong 250014, China
[10]Department of Laboratory Medicine, Chengdu Shangjin Nanfu Hospital/Shangjin Branch of West China Hospital, Sichuan University, Chengdu, Sichuan 611730, China
[11]Shaanxi Key Laboratory of High-Orbits-Electron Materials and Protection Technology for Aerospace, School of Advanced Materials and Nanotechnology, Xidian University, Xi'an, Shaanxi 710126, China
[12]School of Control Science and Engineering, Shandong University, Jinan, Shandong 150061, China
[13]Shaanxi Provincial Cancer Hospital, Xi'an, Shaanxi 710061, China
[14]Key Laboratory of Basic and New Drug Research of Tradinonal Chinese Medicine, Shaanxi Universily of Chinese Medicine, Xianyang, Shaanxi 712046, China
[15]School of Computer Science and Engineering, Xi'an University of Technology, Xi'an, Shaanxi 710048, China
[16]These authors contributed equally
[17]Lead contact
*Correspondence: chen-renan@163.com (R.C.), sdqlyysnz@email.sdu.edu.cn (N.S.), hanmin@sdfmu.edu.cn (M.H.), qzeng@xidian.edu.cn (Q.Z.), zengtingting80@scu.edu.cn (T.Z.), xlchen@xidian.edu.cn (X.C.)
https://doi.org/10.1016/j.xcrm.2025.102320

SUMMARY

Rapid identification and accurate diagnosis are critical for individuals with acute leukemia (AL). Here, we propose a combined deep learning and surface-enhanced Raman scattering (DL-SERS) classification strategy to achieve rapid and sensitive identification of AL with various subtypes and genetic abnormalities. More than 390 of cerebrospinal fluid (CSF) samples are collected as targets, encompassing healthy control, AL patients, and individuals with other diseases. Sensitive SERS detection could be achieved within 5 min, using only 0.5 μL volume of CSF. Through integrated feature fusion (1D spectra and 2D image) with a transformer model, the classification method is developed to screen and diagnose AL patients, demonstrating exceptional classification performances of accuracy, sensitivity, specificity, or reliability. Also, this approach demonstrates remarkable versatility and could be extended to the classifications of meningitis diseases. The sensitive DL-SERS classification platform has the potential to be a powerful auxiliary *in vitro* diagnostic tool.

INTRODUCTION

Central nervous system leukemia (CNSL) is the main cause of leukemia relapse and drug resistance development and potentially results in serious complications and life-threatening situations. Rapid identification and immediate symptomatic treatment are of paramount importance for patients with CNSL.[1,2] Most CNSL patients have no symptoms or atypical symptoms

© 2025 The Author(s). Published by Elsevier Inc.
This is an open access article under the CC BY license (http://creativecommons.org/licenses/by/4.0/).

in the early stage. The effective identification, diagnosis, and prevention of CNSL represent significant challenges in the current landscape of leukemia research. Moreover, a challenge in the diagnosis and treatment of CNSL stems from the ambiguity surrounding its pathogenesis, characterized by a lack of clear theoretical explanations and supporting evidence. Current methods for the clinical diagnosis of CNSL include neurological symptom evaluation, cerebrospinal fluid (CSF) evaluation by lumbar puncture, and radiological imaging.[3] Among these, the conventional cytological (CC) examination of leukemia cells in CSF is considered the gold standard diagnostic method for the identification of CNS involvement. Although the detection specificity of CC examinations is more than 95%, the sensitivity is relatively low (<50%), resulting in frequent false negative results. Recent studies have shown that flow cytometry (FCM) has superior specificity and sensitivity for detecting CNS leukemia compared to CC.[4,5] However, infiltrated leukemia cells adhere easily to the inner wall of meningeal blood vessels, resulting in a decrease in abnormal cell content in CSF. The sensitivity of FCM is inadequate to meet clinical needs. Moreover, FCM diagnosis also requires high levels of expertise in handling, storing, and processing samples.

The measurement and evaluation of CSF biomarkers released or secreted by leukemia cells might represent a more sensitive, accurate, and quantitative diagnostic approach compared to cytological detection methods.[2] For instance, CSF interleukin-6 has been reported to be a promising marker for the diagnosis of adult acute myeloid leukemia (AML) with CNS involvement.[6] Several soluble biomarkers secreted by the CSF, such as leukemic-derived vesicles, secreted proteins, metabolites, and cell-free DNA, have also been discussed.[7–9] CSF biomarkers can also reflect the multiple biological mechanisms of CNS involvement, such as migration and adhesion to the CNS matrix, metabolic plasticity, and cell crosstalk, which are important for improving our understanding of CNSL pathophysiology. CSF biomarker-based CNSL detection and diagnosis is becoming an emerging research focus. In clinical settings, CNSL occurs frequently in acute lymphoblastic leukemia (ALL) patients and AML patients with acute myelomonocytic leukemia (M4) or acute myelomonocytic leukemia (M5) subtypes, and the risk of children with ALL developing CNSL is much higher than that in adults.[10] Therefore, there is significant value in developing a simple, rapid, and sensitive technique for the classification and diagnosis of AL or CNSL.

Surface-enhanced Raman scattering (SERS) is a powerful molecular fingerprint spectroscopy technique characterized by its high sensitivity, excellent specificity, fast detection speed, simplicity of use *in situ*, and multi-component identification.[11,12] The development of artificial intelligence (AI) has enabled the rapid development of the SERS technique. This includes advancements in the design of plasmonic-enhanced nanomaterials, selection and design of reporter molecules, and interpretation and processing of spectral data.[13] In particular, AI-assisted SERS detection strategies have attracted extensive attention in the fields of early detection, disease classification and diagnosis, and treatment evaluation of different cancers.[14–16] Recently, several studies have reported on the detection and diagnosis of leukemia using Raman spectroscopy or SERS technology. These studies include the identification of AML cells and healthy white blood cells, characterization of chemicals and biomarkers, discrimination of AML subtypes, and analysis of mutant gene expression in leukemia cells.[17–22] However, these studies mainly focus on cell identification, component characterization, and subtype classification, and there are almost no studies on CNSL.

Here, CSF samples with relatively simple biochemical components were selected as the target substances, and a reliable SERS detection method based on focused and space-confined hotspots was established. Then, a combined DL-SERS classification strategy was developed to identify healthy controls and various categories or subtypes of AL, encompassing AML or ALL, subtypes of AML and ALL, the subtypes of B-ALL with genetic abnormalities or breakpoint cluster region/abelson murine leukemia viral oncogene homolog1 (BCR/ABL) relevant gene (e.g., Ph+ with p190 or p210, and Ph-like), and AL with CNS involvements. Compared to existing leukemia detection techniques, the combined deep learning and surface-enhanced Raman scattering (DL-SERS) classification method may offer the following potential benefits: (1) Fast and simple: only 0.5 μL of CSF sample was required without any pretreatment, and the spectral data can be obtained within 5 min; (2) excellent classification performance: for various AL subtypes and categories, the prediction accuracy, sensitivity, and specificity can exceed 90%; and (3) generalizability and transferability: the data fusion- and transformer-driven DL-SERS classification method could be extended to the diagnosis of other CNS-related diseases, such as meningitis.

RESULTS

Datasets and study design

We collected 332 CSF samples from five different medical institutions across the country, comprising 268 cases from individuals with acute leukemia and 64 samples from healthy controls. The healthy control cohort primarily consisted of individuals presenting with dizziness or headaches, who were subsequently ruled out for leukemia, tumors, infections, and other organic lesions through a series of clinical examinations. In other words, these participants' symptoms were idiopathic and unrelated to the diseases under investigation. The disease cohort encompassed patients with various subtypes of acute leukemia, including AML, ALL, and their respective subcategories. In accordance with the French-American-British classification and leukemia cell types, the AML patients in this study were categorized into three groups: M1/M2 (acute myeloblastic leukemia with minimal maturation/maturation, 36 samples), M3 (APL, acute promyelocytic leukemia, 32 samples), and M4/M5 (acute myelomonocytic/monocytic leukemia, 43 samples). The ALL patients were divided into two subtypes: B-cell acute lymphoblastic leukemia (B-ALL) and T-cell acute lymphoblastic leukemia (T-ALL). Given the lower cure rate and incidence of T-ALL compared to B-ALL, the sample number of T-ALL (30 samples) in the study cohort was significantly lower than that of B-ALL (67 samples). Based on genetic abnormalities, the B-ALL disease cohort was further subclassified into genetic abnormalities and genetic normal groups or BCR/ABL fusion gene positive and negative groups. Furthermore, patients with CNS involvement,

CellPress
OPEN ACCESS

as determined through repetitive cytological examinations or FCM, were classified into a separate CNS involvement cohort, which included 11 patients. The control group for the CNSL cohort was selected by the following method: after undergoing 2–3 rounds of cytological and FCM tests, patients with different subtypes of acute leukemia were diagnosed as not having CNS infiltration. Within this range, 11 cases of patients were randomly selected as the control group.

Table 1 provides a summary of demographic and clinical characteristics of individuals in both the healthy and disease cohorts, including age, gender, diagnostic outcomes, and sample sizes. The 90% confidence interval for age in the healthy cohort was 45 ± 3 years, whereas, for the disease cohort, it was 36 ± 2 years. In the study, at least 50 of SERS spectral points were randomly collected from every individual in both the healthy and disease cohorts to form the spectral dataset. A dataset comprising 12,139 spectra from 212 patients was utilized for the training, validation, and testing of the acute leukemia disease classification model, while an additional dataset of 6,000 spectra from 120 patients was employed for external independent validation.

Combined DL-SERS classification strategy

The combined DL-SERS classification strategy encompasses several components: SERS spectral data acquisition, deep learning classification method, AL screening, diagnosis, and generalizability validation of classification methods. The SERS detection consists of three parts: collection of CSF specimens from clinical individuals, SERS spectra acquisition, and data preprocessing. An enrichment-based SERS strategy was employed to achieve the acquisition of highly sensitive spectral data (Figure 1A). Transformer algorithm-based deep learning classification model was utilized to perform binary, ternary, or multiclass classification (Figure 1B). Subsequently, a comprehensive disease classification model integrating screening, diagnosis, and generalizability validation was constructed based on the DL-SERS classification method (Figures 1C–1E).

For the deep learning classification approach, the integration of one-dimensional (1D) spectra and two-dimensional (2D) image was used as the input data. Principal-component analysis (PCA) was utilized to extract features from the 1D spectra, while the ResNet50 architecture was engaged for feature extraction from the 2D image. Subsequently, these two categories of features were combined through a matrix concatenation approach. The transformer algorithm was implemented to identify, process, and generate features, yielding the corresponding attention weights. The weight vectors were thereafter normalized and linearly manipulated and subsequently added to the original data to obtain the output. Eventually, multi-layer perceptrons (MLP) and fully connected layers were employed to generate the classification outcomes. The optimized model derived from the validation set was adopted as the final classification model for the subsequent classification and prediction. In the data partitioning process, CSF samples were collected from 332 patients across five medical centers, comprising 268 patients with acute leukemia and 64 healthy individuals. These samples were randomly divided into two cohorts: a primary cohort (n = 212) for model development and an external validation cohort (n = 120) for independent testing. For each CSF sample, 50 of Raman spectra were acquired, resulting in an internal dataset (primary cohort) and an external test dataset (external validation cohort). Next, the internal dataset was further partitioned into training (80%), validation (10%), and internal test (10%) sets in an 8:1:1 ratio to facilitate model optimization and performance assessment (Figure S1A). The external validation cohort, consisting of 120 independent CSF samples, was reserved exclusively for evaluating the model's generalizability.

The validation accuracy served as an index to evaluate the performance and reliability of the transformer-based classification model. As the learning iterations ranged from 0 to 300, the accuracy values of the validation set progressively stabilized and converged for different classification models, signifying the absence of overfitting. The model was trained with an optimized iteration count of 100, attaining outstanding validation accuracies ranging from 0.9 to 0.95, without triggering early stopping (Figure S1B). To better illustrate the key spectral features used for classification, we further visualized the relationship between the extracted features and the original spectrum. Taking the classification of ALL/AML as an example, we randomly selected a representative spectrum and marked the top 150 PCA features on the original spectrum using red dashed lines (Figure S2). The visualization of attention maps for random samples revealed that high-correlation feature regions contained spectral characteristics, which may further enhance the classification model's credibility. At last, we investigated the impact of the selected horizontal coordinate parameters on the spectral classification results. For the binary classification of B-ALL/T-ALL, we set the range of the horizontal coordinate as follows: 99–1,000, 99–2,000, 99–3,000, and 99–3,200 cm^{-1}, respectively. The comparative analysis revealed that a broader range of the horizontal coordinate resulted in higher classification accuracy (Figure S3). Accordingly, when extending the number of extracted features to 1,024, the selected points broadly cover all major spectral peaks. These features were then used to construct the classification model. Also, the visualization data were presented based on attention maps for various classifications (Figure S4).

A variety of evaluation metrics, including the F_1 score, accuracy, sensitivity, and specificity, were utilized to assess diverse classification models, which could be computed using the following formulas:

F_1 score = 2 × (prediction precision × prediction sensitivity)/(prediction precision + prediction sensitivity).

Prediction precision = true positive/(true positive + false positive).

Prediction accuracy = (true positive + true negative)/(true positive + false positive + true negative + false negative).

Prediction sensitivity = true positive/(true positive + false negative).

Prediction specificity = true negative/(false positive + true negative).

Screening for acute leukemia

Figure 2A represents the working principle of the proposed SERS platform for CSF detection, mainly derived from focused and space-confined "hotspots." Two kinds of signal-enhanced approaches are proposed, including the physical enrichment by

Table 1. Characteristics of the primary and external test datasets

		Primary cohort					External validation cohort					Entire dataset
				Gender					Gender			
		No. of subjects	No. of SERS spectra	Male	Female	Age in years (range)	No. of subjects	No. of SERS spectra	Male	Female	Age in years (range)	No. of subjects
Total		212	12,139	87 (41.0%)	125 (59.0%)	39 ± 1 (14–79)	120	6,000	56 (46.7%)	64 (53.3%)	36 ± 2 (15–61)	332
Control cohort		64	3517	25 (39.1%)	39 (60.9%)	45 ± 3 (14–79)	/	/	/	/	/	64
Leukemia cohort		148	8,622	62 (41.9%)	86 (58.1%)	36 ± 2 (14–67)	120	6,000	56 (46.7%)	64 (53.3%)	38 ± 7 (15–61)	268
Acute myeloid leukemia (AML)		81	4,828	34 (41.9%)	47 (58.1%)	37 ± 2 (15–58)	15	750	7 (46.7%)	8 (53.3%)	34 ± 6 (15–52)	/
Acute lymphoblastic leukemia (ALL)		67	3,794	28 (41.8%)	39 (58.2%)	34 ± 3 (14–67)	15	750	11 (73.3%)	4 (26.7%)	37 ± 8 (16–61)	/
AML	M1/M2 subtypes	26	1,577	12 (46.2%)	14 (53.8%)	37 ± 5 (15–58)	10	500	2 (20%)	8 (80%)	29 ± 6 (15–47)	/
	M3 subtypes	22	1,445	7 (31.8%)	15 (68.2%)	40 ± 5 (17–55)	10	500	3 (30%)	7 (70%)	44 ± 6 (19–52)	/
	M4/M5 subtypes	33	1,806	15 (45.5%)	18 (54.5%)	36 ± 3 (15–55)	10	500	5 (50%)	5 (50%)	37 ± 8 (15–54)	/
ALL	T cell ALL (T-ALL)	15	883	10 (66.7%)	5 (33.3%)	35 ± 6 (16–50)	15	750	9 (60%)	6 (40%)	31 ± 6 (16–50)	/
	B cell ALL (B-ALL)	52	2,911	18 (34.6%)	34 (65.4%)	34 ± 3 (14–67)	15	750	4 (26.7%)	11 (73.3%)	39 ± 7 (16–57)	/
B-ALL	B-ALL without genetic abnormalities	17	927	4 (23.5%)	13 (76.5%)	35 ± 6 (14–61)	15	750	6 (40%)	9 (60%)	34 ± 7 (16–57)	/
	B-ALL with genetic abnormalities	35	1,984	14 (40%)	21 (60%)	34 ± 4 (16–67)	15	750	9 (60%)	6 (40%)	39 ± 7 (15–61)	/
	BCR/ABL fusion gene positive	29	1,404	12 (41.4%)	17 (58.6%)	37 ± 4 (17–67)	/	/	/	/	/	/
	BCR/ABL fusion gene negative	23	1,507	6 (26.1%)	17 (73.9%)	31 ± 5 (14–61)	/	/	/	/	/	/
Central nervous system (CNS) involvements		11	554	4 (36%)	7 (64%)	42 ± 7 (20–54)	/	/	/	/	/	/

CellPress
OPEN ACCESS

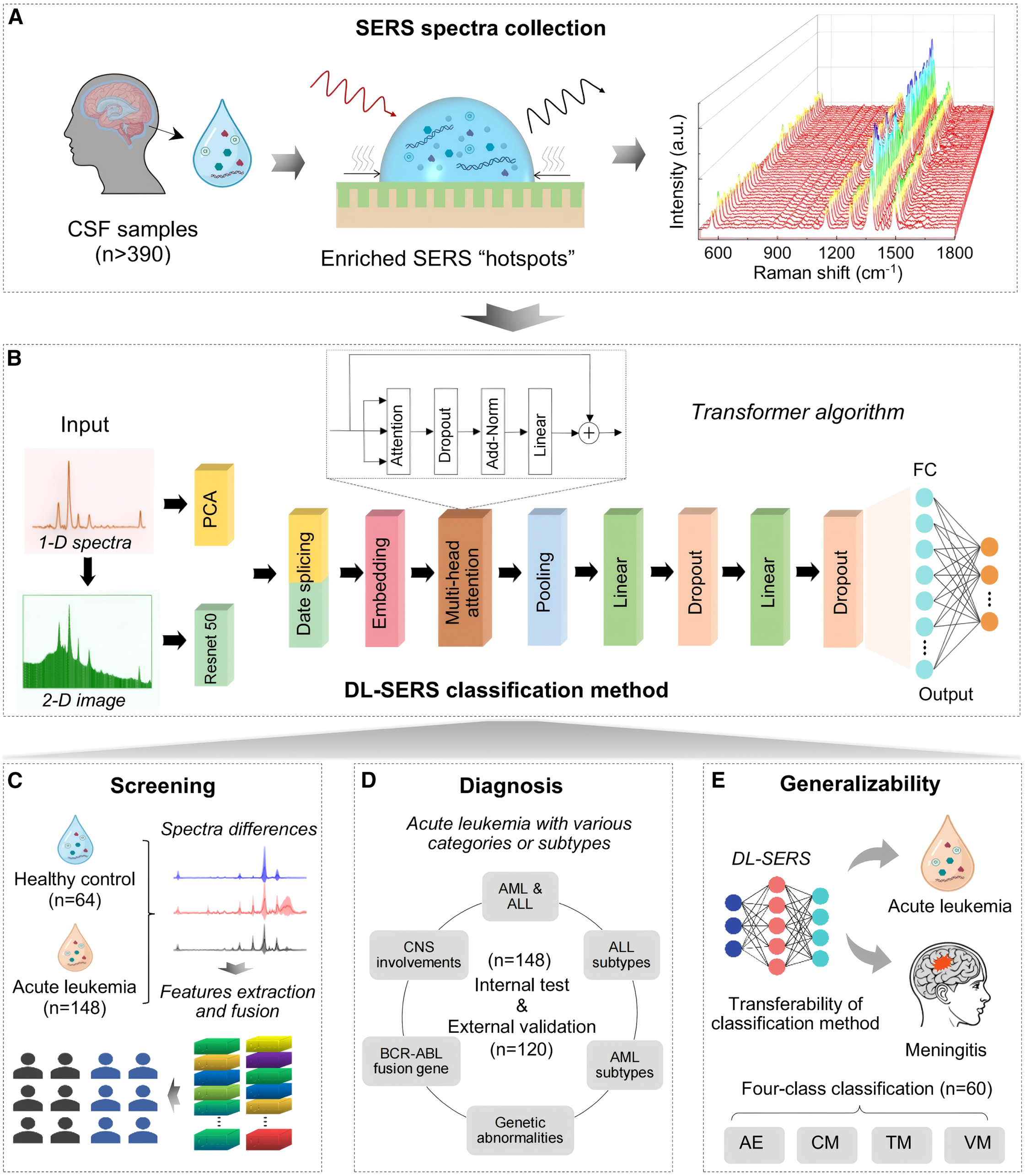

Figure 1. Overview of the combined deep learning-assisted SERS (DL-SERS) classification strategy

(A) Collection of SERS spectra data. CSFs collected from more than 390 individuals are used as detection targets. Spectral data are acquired based on the enriched SERS "hotspots." For each CSF sample, at least 50 of spectral curves were collected randomly to improve the signal reliability.

(B) DL-SERS classification. The classification model comprised several parts: pretreatments of one-dimensional (1D) spectra and two-dimensional (2D) image, date splicing and fusion, transformer classification, and output by multi-layer perception (MLP) mechanism.

(C) The screening model takes syncretic spectral and image data as inputs and outputs the related binary classification to discriminate acute leukemia.

(D) The diagnostic model is established for the classifications of AL with different categories or subtypes, including AML or ALL, subtypes of AML or ALL, the subtypes of B-ALL with genetic abnormalities or BCR/ABL relevant gene, and AL with CNS involvements.

(E) The generalizability of DL-SERS classification. Various subtypes of meningitis are employed, including autoimmune encephalitis, novel cryptococcal meningitis, viral meningitis, or tuberculous meningitis.

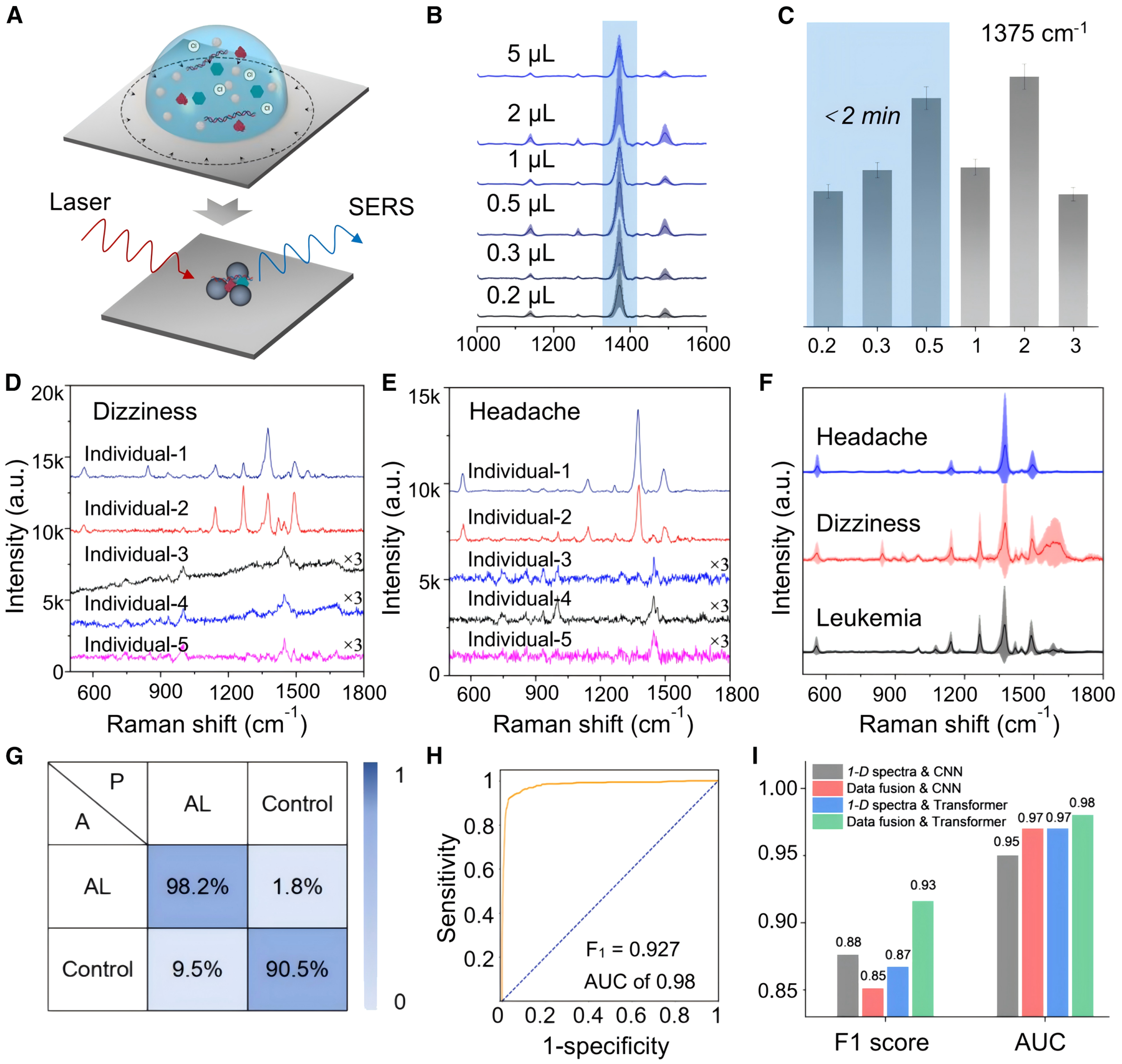

Figure 2. Screening for acute leukemia

(A) Schematic of the working principle of SERS detection. Two signal enhanced approaches are proposed: the physical enrichment by slippery liquid-infused porous surface (SLIPS) and the aggregation effect by NaCl (120–130 mM) contents in CSF.

(B and C) SERS spectra and compared intensities of CSFs with different volumes ranging from 0.2 to 5 μL.

(D and E) SERS spectra of CSFs collected from healthy individuals experiencing feelings of dizziness and headaches. All people have excluded the presence of leukemia, tumors, infections, and other organic lesions.

(F) Compared SERS spectra for acute leukemia and healthy individuals.

(G) The confusion matrix of binary classification for screening of acute leukemia and healthy individuals.

(H) The receiver operating characteristic curves (ROCs) of binary classification for screening of acute leukemia and healthy individuals.

(I) Compared classification performance of various deep learning methods, including feature fusions (1D spectra and 2D image) combined with transformer (data fusion & transformer), 1D spectra combined with transformer (1D spectra & transformer), features fusion combined with convolutional neural network (data fusion & CNN), and 1D spectra combined with CNN (1D spectra & CNN).

slippery liquid-infused porous surface (SLIPS) and the chemical aggregation effect by NaCl (~120–135 mM) contents in CSF samples. Colloidal Ag nanoparticles (NPs) with an extinction peak of 430 nm were employed as SERS-active materials, and several pretreatments, including concentration, purification, and mixing, were conducted before signal collection (Figures S5A and S5B). KI solution with concentration of 100 mM was used for surface purification, resulting in blank and interference-free Raman spectra (Figure S5C). SLIPS was used as the supporting substrate for the evaporation of CSF and Ag NP solutions, and interference-free Raman spectra could be obtained for the blank SLIPS substrate (Figure S5D). Based on the SLIPS substrates, the SERS curve of Ag NPs/CSF sample could be collected with a high signal-to-noise ratio, while almost no obvious signal was obtained for the CSF control or Ag NPs/KI control (Figure S5E). Figure S6 showed the scanning electron microscopy characterization of Ag NPs before and after mixing with CSF, which indicated that obvious Ag NPs aggregation occurred upon mixing with CSF.

The optical image of dried SERS substrates was characterized as shown in the inset image (Figure S7A). The signal intensity could be enhanced by 3–5 times when spectra data were collected in the bright area rather than a dark area, which may be ascribed to the various aggregation states of colloidal Ag NPs. Then, we investigated the effect of incubation time (0, 5, 10, and 30 min) between colloidal Ag NPs and CSF on SERS intensity. The results indicate that prolonged incubation leads to a decrease in the SERS spectral signal, likely due to the chloride components in CSF causing aggregation of the silver colloids, thereby reducing the concentration of plasmonic nanoparticles (Figure S7B). The effects of drying temperature and centrifugation pretreatments on the resulted SERS spectra of CSF samples from various individuals were evaluated, and no obvious variations were observed in characteristic peaks by varying the parameters (Figures S7C, S7D, and S7F). The volume of CSF sample was controlled from 0.2 to 5 μL during droplet evaporation, and the comparative SERS spectra were obtained from different collection points (Figures 2B and S7E). For the 1,375 cm^{-1} peak, the SERS intensities showed no significant changes in the magnitude, and a volume of 0.5 μL was selected for subsequent data collection considering the drying time (less than 2 min) and ease of operation (Figure 2C). Furthermore, the consistency of the obtained SERS signal was evaluated, as shown in Figure S8, and the spectra exhibited excellent consistency with a relative standard deviation (RSD) value of 12.18% at the 1,375 cm^{-1} band.

Spectral data from a healthy cohort comprising 32 individuals with dizziness and 32 with headaches, as well as a disease cohort of 148 cases, were measured using the optimized SERS detection method described earlier. At least 50 spectral curves were acquired for each CSF sample, and average SERS spectra were plotted to mitigate the influence of random events. From the 64 participants in the healthy group, 5 individuals with dizziness and 5 individuals with headache were selected to demonstrate that individual variability may lead to significant changes in spectral characteristics. As depicted in Figures 2D and 2E, after smoothing, denoising, and baseline correction, the SERS spectra of different individuals in the healthy cohort exhibited substantial variations in both characteristic peak positions and intensities. Furthermore, the SERS spectra of 64 participants in the healthy group were statistically analyzed. The results showed that most of the dizziness and headache samples exhibited better peak position consistency and signal-to-noise ratio (Figure S9). Additionally, the overall average spectra of 64 control group samples were plotted, and 9 of major characteristic peaks were extracted for intensity statistics, including 440; 560; 1,002; 1,140; 1,266; 1,374; 1,417; 1,447; and 1,488 cm^{-1} (Figure S10A). To better demonstrate the individual variability within the healthy group, the heatmap was constructed to reflect the intensity information at the aforementioned characteristic peak positions and the corresponding biochemical component contents of different samples (Figure S10B).

An overlay of SERS spectra from all subjects is presented in Figure 2F, illustrating a comparison between the healthy and disease groups, with the disease group showing reduced fluctuation and peak position variability. Artificial CSF (ACSF) was also prepared based on the reported components and concentrations (Table S1), and SERS spectral data were collected (Figure S11). Also, we found that ACSF and CSF samples from AL patients or healthy control exhibited different characteristic peaks. Table S2 presents the clinical test results of CSF samples from different subjects, including appearance characteristics, the content of main biochemical components, and the cell content in CSF. It is considered that the differences in SERS spectra between individuals in the healthy or diseased cohort are mainly caused by the content differences of cell secretions and biological components in CSF specimens.

Due to the impact of inter-individual variability, classification and prediction of unknown individuals based on the characteristic peak positions or intensities of CSF spectra were not feasible. Consequently, a dataset of 12,139 spectral data, including 3,517 from the healthy cohort and 8,622 from the acute leukemia cohort, was input into a deep learning classification model for training, validation, and testing. Based on transformer algorithm combined with feature fusion (1D spectra and 2D image) method, the confusion matrix results indicated a classification accuracy of 96.13% for distinguishing between the healthy and disease groups (Figure 2G). The F_1 score and area under the curve (AUC) values reached 0.927 and 0.98 (Figure 2H), respectively, demonstrating excellent predictive sensitivity and specificity. In addition, various deep learning algorithms and data input modalities were employed to investigate the impact on the classification performances of disease screening model, including the fusion of features (1D spectra and 2D images) integrated with the transformer algorithm (data fusion and transformer), 1D spectra combined with the transformer (1D spectra and transformer), feature fusion combined with CNN (data fusion and CNN), and 1D spectra integrated with CNN (1D spectra and CNN). As depicted in Figure 2I, the approach employing data fusion and transformer yielded a significantly higher F_1 score and AUC value compared to the other three deep learning classification methods.

Diagnosis for acute leukemia

The diagnostic method based on data fusion and transformer was primarily utilized for the identification and classification of various subtypes or disease stages of acute leukemia, including patients with AML or ALL, subtypes of AML and ALL, B-ALL patients with genetic or BCR/ABL-relevant fusion gene abnormalities, and AL patients with CNS involvements (Figure S12A). Firstly, SERS spectra of CSF samples from patients with various types of acute leukemia were measured and analyzed. The comparative SERS data revealed a similar distribution of characteristic peaks for individuals with T-ALL and B-ALL without genetic abnormalities, which were located at 560; 1,139; 1,265; 1,376; and 1,488 cm^{-1} (Figure S12B). The SERS spectral data from patients with T-ALL and B-ALL without genetic or BCR/ABL fusion gene abnormalities exhibited well inter-individual uniformity for characteristic peak locations (Figures S12C, S12D, S12F, and S13–S15). Then, for each group, we randomly selected 10 patients, and, for each patient, we randomly chose 10 of SERS spectra. The average spectrum and corresponding visual heatmap were plotted. The results showed that there were no significant differences in the positions of the major

peaks among individuals (Figure S16). However, for T-ALL, B-ALL without genetic abnormalities, and BCR/ABL-negative ALL, the RSD values of the peak intensity at 1,374 cm^{-1} were 41.1%, 39.0%, and 50.7%, respectively. The observed fluctuations in signal intensity may be attributed to the inter-individual differences among patients, including disease severity or status, age, gender, etc., leading to variations in the contents of different biochemical components in CSF.

However, for patients with genetic abnormalities or BCR/ABL fusion gene alterations, the spectral variability among individuals was significant (Figures S12E, S12G, S17, and S18). For instance, B-ALL patients with various BCR/ABL-related genetic abnormalities, such as BCR/ABL P190 positive with deletion of multiple exon heterozygosity, BCR/ABL positive with IKZF1 large fragments missing, and BCR/ABL-like individuals, were employed to collect SERS data of CSF samples (Figure S18). Differential peaks were identified at 803; 914; 1,169; 1,376; 1,583; and 1,618 cm^{-1}, which could be attributed to various vibrational modes including C-C-O stretching, ring breathing vibrations, C–N stretching, COO^{-} symmetric stretching, C=C bending, and C=C in-plane bending, respectively (Figure S12G; Table S3). The individuals in the BCR/ABL genetic abnormality cohort exhibited different subtypes, such as BCR/ABL (Ph+, p190 or p210), or Ph-like ALL (Table S4). Also, these patients were usually accompanied by multiple genetic abnormalities, e.g., heterozygous or large fragment deletions of multiple gene exons, T315I gene mutation, SSBP2/CSF1R fusion genetic positive, and so on. Figure S19 depicts the SERS spectra of CSF samples from patients with different AML subtypes (M1–M5), exhibiting similar characteristic peaks at 560; 1,001; 1,139; 1,265; 1,376; 1,419; and 1,445 cm^{-1}. Only slight spectral fluctuations were observed ranging from 1,500 to 1,650 cm^{-1}. Additionally, SERS spectra from the same individual with AML demonstrated well inter-individual uniformity for characteristic locations (Figures S20–S22).

Based on the SERS spectral data from AL patients, a multi-functional diagnostic model was constructed using the data fusion and transformer-based deep learning network, encompassing six distinct binary or ternary classifications. The primary dataset comprised 8,622 spectra from 148 individual patients. Figure 3 illustrates the disease prediction outcomes of different classification models, including confusion matrices and receiver operating characteristic (ROC) curves. The prediction accuracies for different types of AL patients were as follows: 96.2% (AML/ALL), 96.5% (B-ALL/T-ALL), 91.8% (AML subtypes, M1&M2, M3, and M4&M5), 94.3% (B-ALL genetic normal or abnormal), 93.9% (BCR/ABL fusion gene positive or negative), and 95.9% (CNS involvement or not) (Figures 3A–3F). Correspondingly, the AUC values for these six diagnostic outcomes ranged between 0.98 and 0.99 (Figures 3G–3L). Table 2 presents a summary of evaluation indicators for different classifications, including F_1 score, accuracy, sensitivity, and specificity. In addition to prediction accuracy, the results also demonstrated high sensitivity (91.7%–97.3%), specificity (91.6%–95.9%), and F_1 scores (0.921–0.969). Furthermore, the advantages of the data fusion and transformer approach in various classification models were validated. As shown in Figure S23, compared to the other three alternative methods, i.e., 1D spectra and transformer, data fusion and CNN, and 1D spectra and CNN, the data fusion combined with transformer algorithm exhibited the best predictive accuracy in different binary or ternary classifications. For instance, in the triple model distinguishing various AML patient subtypes, the accuracy performances for 1D spectra and CNN, 1D spectra and transformer, data fusion and CNN, and data fusion and transformer were 76.3%, 80.0%, 82.3%, and 91.8%, respectively. In addition, we performed significance testing to evaluate the differences in classification performance across methods, including F1 score, accuracy, sensitivity, and specificity. As shown in Figure S24, the proposed feature fusion combined with the transformer model demonstrated significant improvements in F1 score, specificity, and accuracy compared with conventional CNN-based classification models.

To assess the reliability of the AL diagnostic model, an external independent validation was conducted using 120 cases of AL patients collected separately from different hospitals. The external test dataset comprised 6,000 of spectral data points, and the detailed clinical information of the patients was provided in Table 1. Preprocessed spectral and imaging data were input into the previously trained data fusion and transformer model. For each binary or ternary classification, every patient would receive a feedback result of 0/1 or 0/1/2, representing different AL types. After triplicate validations of the SERS data for each patient, the final diagnostic result was determined by extracting the two consistent outputs. The predicted outcomes for each patient are summarized in Table S5. The independent validation accuracies for the four classification models were 93.3% (AML/ALL), 90.1% (AML subtypes), 96.7% (B-ALL/T-ALL), and 93.3% (B-ALL with genetic normal or abnormal), demonstrating good consistency and alignment with the previous internal test results (Figure 4A).

Investigation into CNS involvements resulting from various acute leukemia patients was conducted based on the SERS spectra and FCM results. Firstly, B-ALL patients at various CNS infiltration stages were selected for investigation, with CD33, CD34, CD64, CD7, and human leukocyte antigen-D related (HLA-DR) chosen as biomarkers for FCM analysis. The results revealed that the proportions of abnormal primitive B lymphocytes in CSF of these individuals were 0%, 15.3%, and 24%, (Figures 4C–4E). These three CSF samples were collected from two patients with B-ALL. The B-ALL with 0% abnormal primitive B lymphocytes was a female patient, while the CSF samples with different CNS infiltration stages (15.3% and 24%) were from the same male patient. Notably, the proportion of abnormal primitive B lymphocytes increased from 15.3% to 24% within 38 days. Correspondingly, SERS spectral data were also acquired from these CSF samples, as depicted in Figure 4B, demonstrating that the degree of CNS infiltration significantly impacts the peak locations and intensities. For instance, compared to B-ALL with 0% abnormal primitive B lymphocytes and CSF specimens from the high-infiltration stage exhibited enhanced intensities at the 560 and 1,376 cm^{-1} peaks, while the intensities at 1,265 and 1,488 cm^{-1} were notably reduced. The intensity changes of these characteristic peaks may be attributed to variations in the contents of nucleic acids (560; 1,265 cm; and 1,376 cm^{-1}) and proteins (1,488 cm^{-1}). In addition, the CNS involvement results and the corresponding SERS analysis of AL patients from different subtypes were also investigated.

CellPress
OPEN ACCESS

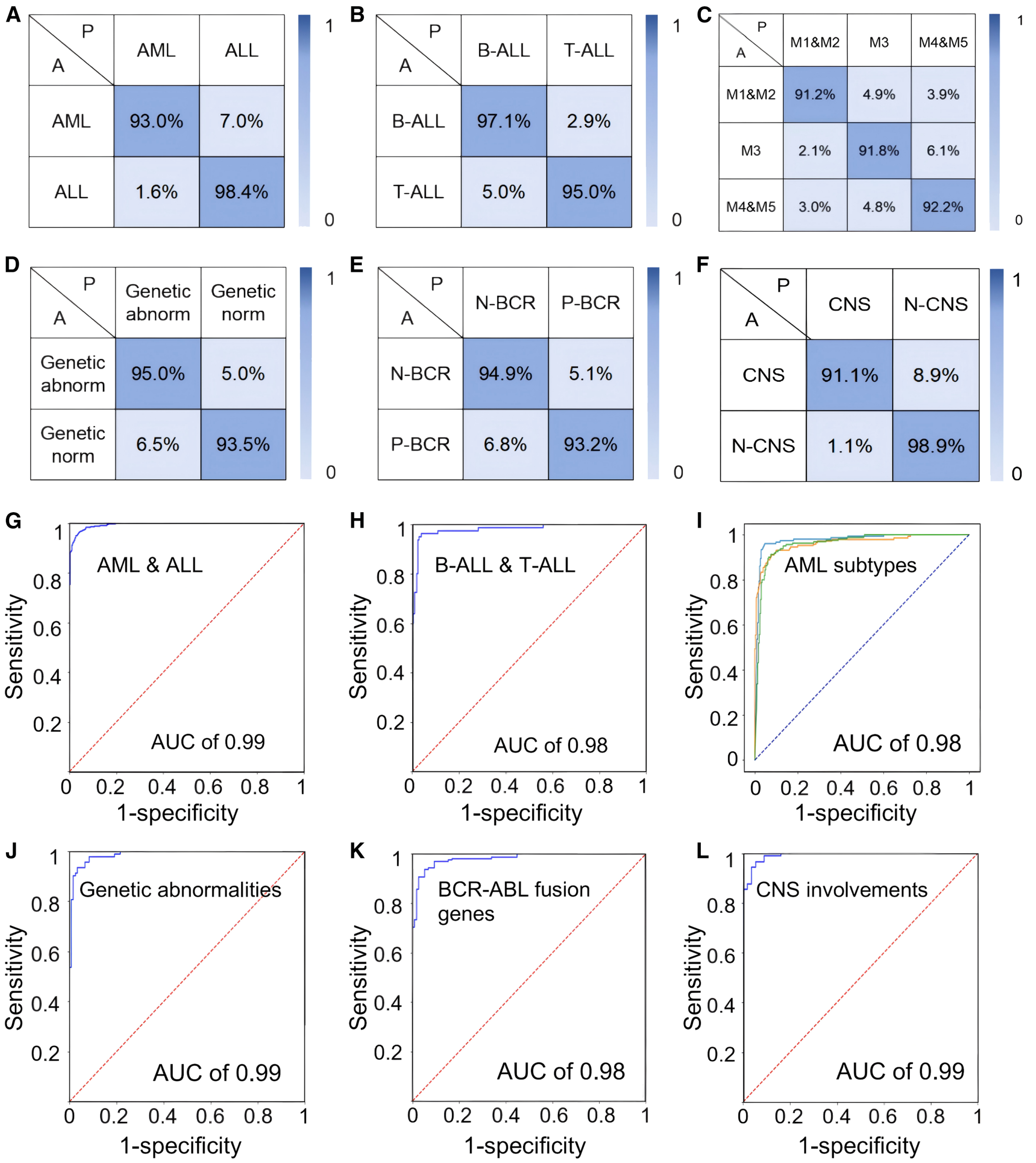

Figure 3. Diagnosis for acute leukemia

(A–F) Confusion matrices of prediction results for identification of AL with different categories or subtypes based on feature fusions (1D spectra and 2D image) combined with transformer. The diagnostic results contain various binary or trinary classifications, including (A) AML/ALL, (B) B-ALL/T-ALL, (C) AML subtypes (M1&M2, M3, and M4&M5), (D) B-ALL chromosome normal or abnormal, (E) BCR/ABL fusion gene positive or negative, and (F) CNS involvements or not.
(G–L) ROCs for the diagnostic of AL with different categories or subtypes, including (G) AML/ALL, (H) B-ALL/T-ALL, (I) AML subtypes, (J) B-ALL chromosome normal or abnormal, (K) BCR/ABL1 fusion gene positive or negative, and (L) CNS involvements or not.

FCM diagnosis revealed the percentage of abnormal cells in CSF to be 35% for AML&M3, 64% for AML&M5, and 43.7% for T-ALL (Figures S25A–25C). The SERS spectra demonstrated significant distinctions among AML&M3, AML&M5, T-ALL, and B-ALL, potentially attributable to biochemical differences in nucleic acids (560; 1,139 cm^{-1}; 1,265 cm^{-1}; 1,376 cm^{-1}; and 1,583 cm^{-1}), glutathione (803 cm^{-1}), glucose (914 cm^{-1}), and amino acids (1,618 cm^{-1}) within the CSF (Figure S25D).

Generalizability and transferability

To validate the generalizability and transferability of the DL-SERS diagnosis platform, meningitis with various subtypes

Table 2. Summarized classification performances of the diagnostic models of acute leukemia with various categories or subtypes

	ALL/AML	B-ALL/T-ALL	AML M1&M2/ M3/M4&M5	B-ALL genetic-norm/abnormal	BCR-ABL positive/ negative	CNS involvement or not
F_1 score	0.969	0.938	0.921	0.967	0.949	0.967
Accuracy	96.2%	96.5%	91.8%	94.3%	93.9%	95.9%
Sensitivity	98.4%	95.0%	91.7%	93.5%	93.1%	98.8%
Specificity	93.0%	97.1%	95.9%	95.0%	94.9%	91.1%
ROC	0.99	0.98	0.97	0.99	0.98	0.99

was selected as the subject of study, with CSF samples from patients collected to obtain SERS spectral data. The dataset comprised a total of 3,000 spectral entries from 60 individuals, distributed across different meningitis patient groups: viral meningitis (15 cases), tubercular meningitis (15 cases), cryptococcal meningitis (15 cases), and autoimmune encephalitis (15 cases). Demographic information such as the number of patients, gender, and age distribution is presented in Table S6. Similar to the diagnosis of acute leukemia, the acquired SERS spectra from meningitis underwent identical processing, encompassing pretreatments of 1D spectra and 2D images, data splicing and fusion, and transformer classification followed by output through an multi-level perception mechanism. Also, 80% of the spectral data were utilized for model training, while the remaining 20% served as the validation and test sets. SERS spectra were plotted for five randomly selected patients from each meningitis subtype (Figures 5A–5D). Notably, spectral data from individuals within the tubercular meningitis group exhibited relatively pronounced intra-group variability, whereas the intra-group variability was lower for the other three subtypes. Subsequently, using the data fusion and transformer model, a four-class classification of meningitis diseases was achieved with a prediction

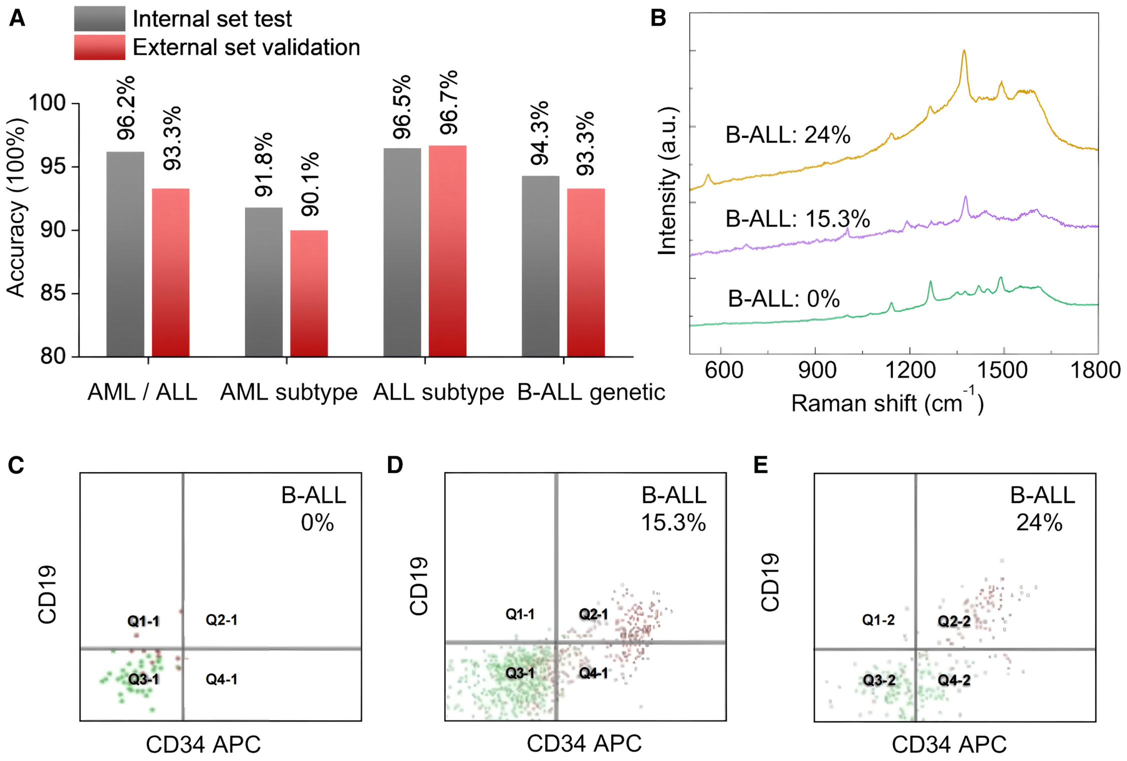

Figure 4. External validation of DL-SERS classification and the flow cytometry analysis

(A) Compared prediction accuracy performances for the internal and external set validation. The diagnostic results contain AML/ALL, ALL subtypes, AML subtypes, and B-ALL with or without genetic abnormalities.

(B) SERS spectra of CSF samples from B-ALL patients with CNS involvements at different stages. The proportions of abnormal primitive B lymphocytes in CSFs were 0%, 15.3%, and 24%.

(C–E) FCM results of CNSL patients originating from B-ALL with varying proportions of abnormal primitive B lymphocytes in CSF: (C) 0%, (D) 15.3%, and (E) 24%. In these figures, the red dots denote leukemia cells, while the green dots signify normal lymphocytes.

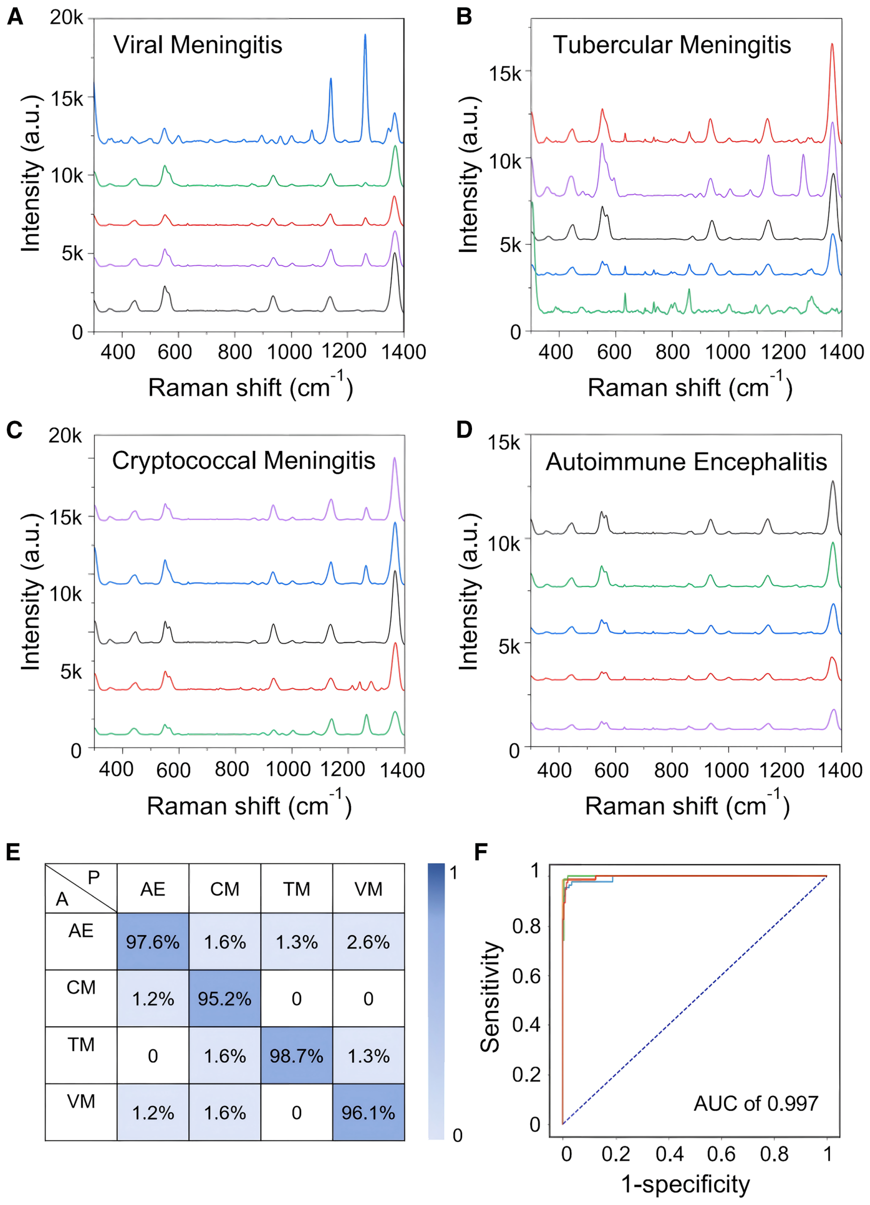

Figure 5. Generalizability and transferability of DL-SERS classification for meningitis

(A–D) SERS spectra of CSF samples collected from meningitis patients with various subtypes, including (A) viral meningitis, (B) tubercular meningitis, (C) cryptococcal meningitis, and (D) autoimmune encephalitis. All patients have been diagnosed through the CSF biochemical testing and cytological methods.

(E) The confusion matrix of four-class classification for various meningitis subtypes.

(F) The ROCs of four-class classification for various meningitis subtypes.

accuracy reaching 97%. The average F1 score, sensitivity, specificity, and AUC values were 0.97, 97.12%, 99%, and 99.7%, respectively (Figures 5E and 5F).

DISCUSSION

In comparison with blood, plasma, or urine samples, CSF generally exhibits a more simplified composition owing to the presence of the blood-brain barrier. It predominantly comprises water; chloride (approximately 120–130 mM); glucose (2.5–4.5 mM); and only trace amounts of proteins, enzymes, or nucleic acids. Virtually no cells can be detected in the CSF of healthy individuals. Hence, as a distinctive human fluid for liquid biopsy, the SERS spectra of CSF samples are more amenable to processing and analysis. The biological metabolites present within the CSF might be intimately correlated with the degree or progression of CNS involvement in different patients. The CSF-based liquid biopsy approach holds promise for the early screening and detection of cerebral or CNS-related disorders.[23] Accordingly, by collecting the SERS spectral data of CSF specimens from various populations, it was discovered that the spectral characteristics of individuals experiencing dizziness and headaches, as well as acute leukemia patients with CNS infiltration or different types of genetic abnormalities, demonstrated pronounced intra-group or inter-group disparities.

As a fingerprint spectroscopic technique with single-molecule sensitivity, SERS detection involves complex interactions among photons, target substances, and the enhanced substrates.[24,25] A crucial challenge during SERS detection lies in how target molecules can be induced and concentrated into SERS “hotspot” regions rapidly and selectively, which is of great significance for enhancing the SERS reliability and sensitivity. Additionally, improving the reliability and accuracy of SERS analysis toward complex clinical samples and disease detection is the key factor limiting its translation to clinical applications. In this study, CSF samples with relatively simple biochemical components were selected as the target substances, and a highly reliable SERS detection method based on focused and space-confined electromagnetic hotspots was established. In addition to the physical enrichment of SLIPS substrate by eliminating the diffusion limits, the chloride components contained in CSF samples can induce the aggregation and enhancement of colloidal nanoparticles and simultaneously promote the plasmonic nanostructure/molecule absorption behavior.[26,27] Consequently, the entire detection process requires only a volume of less than 0.5 μL of CSF sample, yielding sensitive SERS signals for data analysis and diagnostic model construction.

CNS infiltration poses a significant clinical challenge in the field of acute leukemia detection and diagnosis, often leading to severe complications and mortality. This challenge arises because infiltrated leukemia cells readily adhere to the inner wall of meningeal blood vessels, consequently reducing detection sensitivity in the early stages of CNS infiltration. It is reported that CNSL was identified in about 3%–5% of patients at initial diagnosis and in 30%–40% of patients at relapse.[28] Unlike traditional cytological methods, the SERS spectra of CSF samples encompass comprehensive biochemical composition information, including common proteins, glucose, chloride, and CSF biomarkers released or secreted by leukemia cells. Consequently, CSF samples from patients with different AL subtypes or at various stages of CNS infiltration exhibit distinguishable spectral signatures. Furthermore, diagnosing ALL with BCR/ABL1-related genetic abnormalities remains challenging due to genetic heterogeneity and cytogenetic recessive rearrangements, necessitating comprehensive examinations, such as FCM, whole-transcriptome sequencing, fluorescence *in situ* hybridization, or targeted sequencing methods. Here, B-ALL patients with BCR/ABL-related genetic abnormalities, such as Ph+ (p190 or p210), or Ph-like ALL, can also be identified through characteristic fingerprint differences. These differences could be intelligently analyzed and clustered through a DL strategy, and thus a classification and diagnosis method based on SERS spectral data was established. This DL-SERS intelligent analysis and assessment hold promise as a simple, rapid, and accurate auxiliary method for rapidly detecting and assessing AL patients with different subtypes, degrees of CNS involvement, or various genetic abnormalities.

The previously reported AI-assisted SERS strategies mainly utilized networks like convolutional neural networks or artificial neural networks to classify targets based on the 1D characteristics of Raman spectral data.[29–32] Conversely, the proposed DL-assisted classification and diagnostic model described in this study offers advantages in two aspects. Firstly, the fusion and splicing of spectral features from 1D Raman spectra and 2D images were successfully accomplished, and these combined features were used as the input data for subsequent training and testing procedures. The analysis incorporated a variety of SERS spectral features, including intensity information, wavenumber position, and spectral shape, thereby enhancing classification performance.[33] Secondly, the transformer algorithm was applied to train and test the SERS data. Through the multi-head attention mechanism, it became possible to simultaneously focus on features from different locations and dimensions. Compared to the typical CNN algorithm, the data fusion and transformer strategy demonstrated superior performances in terms of prediction accuracy, sensitivity, and specificity. The reliability of this classification method was further verified through independent validation based on multicenter samples. Additionally, this classification method demonstrates versatility and transferability, capable of being extended to the classification and diagnosis of other CNS diseases such as meningitis without altering the algorithm network or framework.

In summary, the DL-SERS-based CSF detection could emerges as a highly sensitive and rapid strategy compared to existing cellular diagnostic techniques. It has the potential to serve as a potent auxiliary *in vitro* diagnostic tool for the identification of acute leukemia or CNS-related diseases.

Limitations of the study

Establishing a precise correlation between experimental SERS spectra and molecular differences in the CSFs remains challenging in this work. In future research, efforts should be made to establish the possible correlation between experimental fingerprint data and molecule information in CSFs or other clinical samples, to clarify the molecular mechanism of the occurrence and development of CNS diseases. In addition, the sample size of CNSL individuals here is small, which may lead to insufficient reliability of the recognition model.

RESOURCE AVAILABILITY

Lead contact

Further information and requests for resources should be directed to and will be fulfilled by the lead contact, Xueli Chen (xlchen@xidian.edu.cn).

Materials availability

This study did not generate new unique reagents.

Data and code availability

- All data and any additional information are available from the lead contact upon request.
- The codes and data including the model, the training engine, the testing script, and the model weight and partial test dataset have been deposited at Zenodo and are publicly available as of the date of publication. DOI is listed in the key resources table.
- Any additional information required to reanalyze the data reported in this work paper is available from the lead contact upon request.

ACKNOWLEDGMENTS

This work has been supported by the National Natural Science Foundation of China, grants 32201133 (D.Z.), 62275210 (X.C.), and 82302632 (Y.S.); the National Leading Talent Program (X.C.); the National Young Talent Program (X.C.); the Shaanxi Young Top-notch Talent Program (X.C.); the Young Talent Fund of Association for Science and Technology in Shaanxi, grant 20230222 (D.Z.); the Science and Technology Projects in Guangzhou, grant 2023A04J1509 (D.Z.); the Guangdong Basic and Applied Basic Research Foundation, grant 2025A1515010789 (D.Z.); the 1·3·5 project for disciplines of excellence, West China Hospital, Sichuan University, grant ZYJC21067 (T.Z.); the Sichuan Science and Technology Program, grant 2022YFS0214 (T.Z.); the Natural Science Foundation of Sichuan Province, grant 2022NSFSC1305 (Y.S.); the Fundamental Research Funds for the Central Universities, grants ZYTS25098 (D.Z.) and QTZX25113 (X.C.); the Xidian University Specially Funded Project for Interdisciplinary Exploration, grant TZJH2024036 (N.W.); and the Opening Foundation of Shanxi Key Laboratory of Ferroelectric Physical Micro-nano Devices and Systems, grant TDWL-202501 (D.Z.). Raman measurement and sample preparation are supported by the Instrument Analysis Center of Xidian University and the Comprehensive Experimental Center for Chemistry and Bioscience of Xidian University. We would like to thank MogoEdit (https://www.mogoedit.com) for its English editing during the preparation of this manuscript.

AUTHOR CONTRIBUTIONS

D.Z., X.C., and T.Z. conceived and designed the study. H.L., Y.P., L.S., X.L., R. L., and Q.Z. conducted the experiments and characterizations. Z.C., Z.R., and N.W. implemented the deep learning algorithm. Y.S., F.H., C. Zong, R.Z., S.C., C. Zhu, Xiaoli Zhang, and M.H. performed the CSF sample collection and clinical testing. C.S., Xiaofei Zhang, X.M., and L.W. provided support for data analysis. X.C., T.Z., R.C., N.S., and Q.Z. supervised the study. D.Z., Z.C., Q.Z., and

CellPress
OPEN ACCESS

X.C. wrote and revised the manuscript. D.Z., Z.C., and Y.S. contributed equally to this work.

DECLARATION OF INTERESTS

The authors declare no competing interests.

STAR★METHODS

Detailed methods are provided in the online version of this paper and include the following:

- KEY RESOURCES TABLE
- EXPERIMENTAL MODEL AND STUDY PARTICIPANT DETAILS
  - Human subjects
  - Sample size estimation and allocation
- METHOD DETAILS
  - Instrumentations
  - Synthesis of Ag NPs
  - Enrichment-based SERS measurement
  - Deep learning-based classification method
- QUANTIFICATION AND STATISTICAL ANALYSIS

SUPPLEMENTAL INFORMATION

Supplemental information can be found online at https://doi.org/10.1016/j.xcrm.2025.102320.

Received: March 10, 2025
Revised: June 17, 2025
Accepted: July 31, 2025
Published: September 8, 2025

REFERENCES

1. Paul, S., and Short, N.J. (2022). Central Nervous System Involvement in Adults with Acute Leukemia: Diagnosis, Prevention, and Management. Curr. Oncol. Rep. *24*, 427–436.
2. Thastrup, M., Duguid, A., Mirian, C., Schmiegelow, K., and Halsey, C. (2022). Central nervous system involvement in childhood acute lymphoblastic leukemia: challenges and solutions. Leukemia *36*, 2751–2768.
3. Del Principe, M.I., Buzzatti, E., Piciocchi, A., Forghieri, F., Bonifacio, M., Lessi, F., Imbergamo, S., Orciuolo, E., Rossi, G., Fracchiolla, N., et al. (2021). Clinical significance of occult central nervous system disease in adult acute lymphoblastic leukemia. A multicenter report from the Campus ALL Network. Haematologica *106*, 39–45.
4. Córdova-Serrano, R.D., Almanza Huante, E., Mártinez-Moreno, E., Hérnandez-Alcántara, A., Fernández-Sánchez, E., Gomez-De Leon, A., and Espinosa, K.A. (2020). Central Nervous System (CNS) Involvement in Adult Acute Lymphoblastic Leukemia (ALL) Assessed Solely By Flow Cytometry Has an Adverse Impact on Survival. Blood *136*, 2–3.
5. Kopmar, N.E., and Cassaday, R.D. (2023). How I prevent and treat central nervous system disease in adults with acute lymphoblastic leukemia. Blood *141*, 1379–1388.
6. Gu, J., Huang, X., Zhang, Y., Bao, C., Zhou, Z., Tong, H., and Jin, J. (2022). Cerebrospinal fluid interleukin-6 is a potential diagnostic biomarker for central nervous system involvement in adult acute myeloid leukemia. Front. Oncol. *12*, 1013781.
7. Egyed, B., Kutszegi, N., Sági, J.C., Gézsi, A., Rzepiel, A., Visnovitz, T., Lőrincz, P., Müller, J., Zombori, M., Szalai, C., et al. (2020). MicroRNA-181a as novel liquid biopsy marker of central nervous system involvement in pediatric acute lymphoblastic leukemia. J. Transl. Med. *18*, 250.
8. Haney, M., Moore, H., Sampathi, S., Chernyavskaya, Y., Badgett, T., and Blackburn, J. (2020). Cell-Free DNA As a Biomarker for Acute Lymphoblastic Leukemia Relapse in the Central Nervous System. Blood *136*, 7–8.
9. Cheung, Y.T., Khan, R.B., Liu, W., Brinkman, T.M., Edelmann, M.N., Reddick, W.E., Pei, D., Panoskaltsis-Mortari, A., Srivastava, D., Cheng, C., et al. (2018). Association of Cerebrospinal Fluid Biomarkers of Central Nervous System Injury With Neurocognitive and Brain Imaging Outcomes in Children Receiving Chemotherapy for Acute Lymphoblastic Leukemia. JAMA Oncol. *4*, e180089.
10. Lenk, L., Alsadeq, A., and Schewe, D.M. (2020). Involvement of the central nervous system in acute lymphoblastic leukemia: opinions on molecular mechanisms and clinical implications based on recent data. Cancer Metastasis Rev. *39*, 173–187.
11. Cutshaw, G., Uthaman, S., Hassan, N., Kothadiya, S., Wen, X., and Bardhan, R. (2023). The Emerging Role of Raman Spectroscopy as an Omics Approach for Metabolic Profiling and Biomarker Detection toward Precision Medicine. Chem. Rev. *123*, 8297–8346.
12. Liu, J., Zheng, T., and Tian, Y. (2019). Functionalized h-BN Nanosheets as a Theranostic Platform for SERS Real-Time Monitoring of MicroRNA and Photodynamic Therapy. Angew Chem. Int. Ed. Engl. *58*, 7757–7761.
13. Guo, S., Popp, J., and Bocklitz, T. (2021). Chemometric analysis in Raman spectroscopy from experimental design to machine learning-based modeling. Nat. Protoc. *16*, 5426–5459.
14. Dong, S., He, D., Zhang, Q., Huang, C., Hu, Z., Zhang, C., Nie, L., Wang, K., Luo, W., Yu, J., et al. (2023). Early cancer detection by serum biomolecular fingerprinting spectroscopy with machine learning. eLight *3*, 17.
15. Shi, L., Li, Y., and Li, Z. (2023). Early cancer detection by SERS spectroscopy and machine learning. Light Sci. Appl. *12*, 234.
16. Yi, J., You, E.M., Liu, G.K., and Tian, Z.Q. (2024). AI–nano-driven surface-enhanced Raman spectroscopy for marketable technologies. Nat. Nanotechnol. *19*, 1758–1762.
17. Ayyappan, V., Chang, A., Zhang, C., Paidi, S.K., Bordett, R., Liang, T., Barman, I., and Pandey, R. (2020). Identification and Staging of B-Cell Acute Lymphoblastic Leukemia Using Quantitative Phase Imaging and Machine Learning. ACS Sens. *5*, 3281–3289.
18. Li, M., Wu, Y., Chi, M., Wang, Y., Zhu, M., and Gao, S. (2023). Recognition of Nucleophosmin Mutant Gene Expression of Leukemia Cells Using Raman Spectroscopy. Appl. Spectrosc. *77*, 689–697.
19. Paidi, S.K., Raj, P., Bordett, R., Zhang, C., Karandikar, S.H., Pandey, R., and Barman, I. (2021). Raman and quantitative phase imaging allow morpho-molecular recognition of malignancy and stages of B-cell acute lymphoblastic leukemia. Biosens. Bioelectron. *190*, 113403.
20. Ye, M., Chen, Y., Wang, Y., Xiao, L., Lin, Q., Lin, H., Duan, Z., Feng, S., Cao, Y., Zhang, J., et al. (2022). Subtype discrimination of acute myeloid leukemia based on plasma SERS technique. Spectrochim. Acta Mol. Biomol. Spectrosc. *271*, 120865.
21. Han, H., Gong, J., and Tian, Y. (2022). Analysis of serum from acute leukemia patients using surface-enhanced Raman spectroscopy (SERS). Spectroscopy *37*, 36–41.
22. Oktem, F., Akdeniz, M., Al-Shaebi, Z., Akyol, G., Keklik, M., and Aydin, O. (2025). SERS and machine learning-enabled liquid biopsy: a promising tool for early detection and recurrence prediction in acute leukemia. ACS Omega *10*, 11887–11899.
23. Miller, A.M., Shah, R.H., Pentsova, E.I., Pourmaleki, M., Briggs, S., Distefano, N., Zheng, Y., Skakodub, A., Mehta, S.A., Campos, C., et al. (2019). Tracking tumour evolution in glioma through liquid biopsies of cerebrospinal fluid. Nature *565*, 654–658.
24. Bi, X., Czajkowsky, D.M., Shao, Z., and Ye, J. (2024). Digital colloid-enhanced Raman spectroscopy by single-molecule counting. Nature *628*, 771–775.
25. Chen, X., Ding, Q., Bi, C., Ruan, J., and Yang, S. (2022). Lossless enrichment of trace analytes in levitating droplets for multiphase and multiplex detection. Nat. Commun. *13*, 7807.
26. Zhang, D., You, H., Yuan, L., Hao, R., Li, T., and Fang, J. (2019). Hydrophobic Slippery Surface-Based Surface-Enhanced Raman Spectroscopy

Platform for Ultrasensitive Detection in Food Safety Applications. Anal. Chem. *91*, 4687–4695.

27. Zhou, Z.M., Zheng, H., Liu, T., Xie, Z.Z., Luo, S.H., Chen, G.Y., Tian, Z.Q., and Liu, G.K. (2021). Improving SERS Sensitivity toward Trace Sulfonamides: The Key Role of Trade-Off Interfacial Interactions among the Target Molecules, Anions, and Cations on the SERS Active Surface. Anal. Chem. *93*, 8603–8612.

28. Pui, C.-H., and Howard, S.C. (2008). Current management and challenges of malignant disease in the CNS in paediatric leukaemia. Lancet Oncol. *9*, 257–268.

29. Shin, H., Choi, B.H., Shim, O., Kim, J., Park, Y., Cho, S.K., Kim, H.K., and Choi, Y. (2023). Single test-based diagnosis of multiple cancer types using Exosome-SERS-AI for early stage cancers. Nat. Commun. *14*, 1644.

30. Huang, L., Sun, H., Sun, L., Shi, K., Chen, Y., Ren, X., Ge, Y., Jiang, D., Liu, X., Knoll, W., et al. (2023). Rapid, label-free histopathological diagnosis of liver cancer based on Raman spectroscopy and deep learning. Nat. Commun. *14*, 48.

31. Xie, Y., Su, X., Wen, Y., Zheng, C., and Li, M. (2022). Artificial Intelligent Label-Free SERS Profiling of Serum Exosomes for Breast Cancer Diagnosis and Postoperative Assessment. Nano Lett. *22*, 7910–7918.

32. Shin, H., Oh, S., Hong, S., Kang, M., Kang, D., Ji, Y.G., Choi, B.H., Kang, K.W., Jeong, H., Park, Y., et al. (2020). Early-Stage Lung Cancer Diagnosis by Deep Learning-Based Spectroscopic Analysis of Circulating Exosomes. ACS Nano *14*, 5435–5444.

33. Qi, Y., Zhang, G., Yang, L., Liu, B., Zeng, H., Xue, Q., Liu, D., Zheng, Q., and Liu, Y. (2022). High-Precision Intelligent Cancer Diagnosis Method: 2D Raman Figures Combined with Deep Learning. Anal. Chem. *94*, 6491–6501.

34. Zhang, D., Peng, L., Shang, X., Zheng, W., You, H., Xu, T., Ma, B., Ren, B., and Fang, J. (2020). Buoyant particulate strategy for few-to-single particle-based plasmonic enhanced nanosensors. Nat. Commun. *11*, 2603.

CellPress
OPEN ACCESS

STAR★METHODS

KEY RESOURCES TABLE

REAGENT or RESOURCE	SOURCE	IDENTIFIER
Biological samples		
cerebrospinal fluids	This paper	N/A
Chemicals, peptides, and recombinant proteins		
silver nitrate (0.1 mol/L)	Aladdin	S116267; CAS No.: 7761-88-8
sodium citrate (≥99%)	Aladdin	S189183; CAS No.:68-04-2
potassium iodide (≥99%)	Sigma	CAS No.: 7681-11-0
Perfluoropolyether lubricating oil (GPL107)	Dupont Krytox	CAS No.: 60164-51-4
Critical commercial assays		
BD FACSCanto™ Clinical Flow Cytometry System	Becton, Dickinson and Company	SFDA Certified No:20192222462
Software and algorithms		
Python (version 3.8.16)	Python Software Foundation	https://www.python.org
PyTorch (version 2.1.1+cu121)	Python Software Foundation	https://www.python.org
Numpy (version 1.23.5)	Python Software Foundation	https://www.python.org
Matplotlib (version 3.7.1)	Python Software Foundation	https://www.python.org
Pandas (version 2.0.3)	Python Software Foundation	https://www.python.org
Seaborn (version 0.12.2)	Python Software Foundation	https://www.python.org
Cuda (version 12.2)	NVIDIA Developer	https://developer.nvidia.com
Cuda (version 8.8.1)	NVIDIA Developer	https://developer.nvidia.com
MATLAB (version 2023a)	MathWorks Developer	https://www.mathworks.com/
Spectra classification algorithm	This paper	https://doi.org/10.5281/zenodo.15968616

EXPERIMENTAL MODEL AND STUDY PARTICIPANT DETAILS

Human subjects

Clinical CSF samples employed in this study were provided by five different medical institutions, including West China Hospital, Xi'an Daxing Hospital, Qilu Hospital of Shandong University Dezhou Hospital, Shandong Provincial Qianfoshan Hospital and Chengdu Shangjin Nanfu Hospital. The collection and usage of clinical samples have been approved by the ethics committees of these five medical institutions. In total, the study contained 392 of CSF samples, including 64 samples received from healthy controls, 268 CSF samples from acute leukemia patients with different types, and 60 cases from meningitis patients. The healthy control cohort were collected from normal person with the feelings of dizziness or headaches, which have not been diagnosed with leukemia or other diseases undoubtedly. The detailed clinical diagnostic information of acute leukemia samples has been measured via different clinical detection methods. Especially, conventional cytological and flow cytometry have been used to identify the CNSL involvement and transfer for various types of acute leukemia. The received CSF samples were stored at −80°C. In order to ensure the biochemical components unchanged for detection and storage, each CSF sample should be used within 3 times of freeze-thaw cycles.

Sample size estimation and allocation

Assuming an expected sensitivity (P) of 0.9, a disease prevalence of 0.05, an allowable error (δ) of 0.1, and a significance level (α) of 0.05, the calculated sample size was determined to be 691, based on the following standard sensitivity estimation formula.

$$n = \frac{Z_{\alpha/2}^{2} P(1 - P)}{0.05\delta^{2}}$$

However, due to practical constraints in CSF sample collection, the final sample size we selected was 392. These samples here could be divided into three distinct groups: healthy controls (n = 64), acute leukemia (n = 268) comprising internal training/validation cohort (n = 148) and independent external validation cohort (n = 120), and meningitis (n = 60). For each CSF sample, 50 of Raman spectra were acquired, thus forming the internal dataset (primary cohort) and external test dataset (external validation cohort). Next, the internal dataset was further partitioned into training (80%), validation (10%), and internal testing (10%) sets. The external validation cohort was employed to evaluate the model's generalizability.

METHOD DETAILS

Instrumentations

The UV spectroscopy measurements were characterized using ultraviolet-visible spectroscopy (Agilent, Carry 60). SERS detection was conducted on a confocal Raman Spectrometer (Renishaw, Invia system). Flow cytometric analysis was conducted using the BD FACSCanto Clinical Flow Cytometry System along with the respective fluorescent antibodies, isotype controls, and CST fluorescent microspheres.

Synthesis of Ag NPs

SERS-active Ag NPs were prepared by the classical sodium citrate reduction method. In brief, 100 mL of $AgNO_3$ solution with the concentration of 1×10^{-3} mol/L was added to a conical flask and heated at 110°C. After boiling, 3 mL of 1% sodium citrate was added to the above solution, and then the mixture was stirred for one hour until the solution color turned to yellow-green. The obtained Ag NPs was stored at 4°C. In order to eliminate the effect of surface adsorbate on the measured SERS spectra, Ag NPs were purified with KI solution, which reported in the previous literature. 1 mM of KI solution mixed with Ag NPs by a volume ratio of 1:1 under room temperature for 20 min, and then the mixture was centrifuged and concentrated to 5-fold increase of Ag NPs concentration.

Enrichment-based SERS measurement

For the enrichment-typed SERS detection, a slippery liquid-infused porous surface (SLIPS) substrate was prepared according to the previous report.[34] In brief, the Teflon membrane was fixed onto a flat glass slide (5 cm × 5 cm) by double-sided adhesive. Then, 0.5 mL Perfluorinated lubricant (Dupont, GPL 105) was dispersed on the membrane by spin coating. Before using as the enriched SERS substrate, the SLIPS need to be dried at 80°C for 10 min. Then, the mixing of clinical CSF sample and Ag NPs on SLIPS substrate was conducted. The CSF samples obtained from hospitals were used directly with any preparatory steps, such as centrifugation, separation, or purification. For the SERS substrate preparation, CSF samples were mixed with Ag NPs in a volume ratio of 1:1. Then, 1–2 μL of the mixed solutions was added to the SLIPS substrate at an evaporation temperature of 80°C, and thus the SERS detection substrate could be achieved within 2 min. In the experiment, a confocal Raman spectrometer was used to collect Raman signal with an excitation wavelength of 532 nm, and at least 50 of spectral curves were collected randomly for each CSF sample. During data acquisition, 1 mW of laser power and 1s of integration time were used for spectra collection.

Deep learning-based classification method

Spectral data preprocessing

Smoothing, denoising and baseline subtraction were taken for achieving a better quality of Raman spectra. Savitzky-Golay was employed for smoothing and denoising, followed by an iterative baseline correction algorithm based on morphology to subtract the baseline background from the spectral data. To enable downstream image-based feature extraction and model fusion, the 2-D image data was generated through 1-D Raman spectral data' transformation, rather than obtained using the Raman spectrometer's mapping function. Here, Raman shift and corresponding intensity values were treated as independent and dependent variables, respectively, with a strict one-to-one mapping maintained across all data points. A linear interpolation algorithm was employed to resample the spectral points, minimizing the sum of squared residuals between adjacent data pairs. This interpolation strategy was selected to preserve key spectral characteristics while mitigating the risk of artificial oscillations-such as Gibbs artifacts-that can result from higher-order interpolation methods. Following interpolation, the data points were sequentially connected using solid lines to generate a continuous spectral curve. The resulting 2-D Raman image visually represents the spatial distribution of signal intensities and the variation of spectral peak shapes across the defined Raman shift range. This dimensional transformation from discrete spectral arrays to continuous image representations enhances both the interpretability and fidelity of the spectral features. The converted 2-D images were subsequently used for convolutional feature extraction and multimodal fusion within the classification framework.

Feature extraction and fusion

Spectral feature extraction and fusion comprised two components: *1-D* data and *2-D* images. The above-mentioned preprocessed SERS spectra were extracted for 1-D features using Principal Component Analysis (PCA), The number of principal components was set to 1,024, each contributing over 95% of the variance. We aimed to extract as many informative components as possible to serve as input features for the model, thereby reducing the risk of overfitting and enhancing the model's generalization capability. Furthermore, ResNet50 network was utilized to extract *2-D* features from the spectral image data. As a deep convolutional neural network model, ResNet50 employed a 50-layer network structure. This approach was characterized by the inclusion of residual connections and skip connections between convolutional layers, which aided in preventing overfitting and enhancing model performance. After obtaining the *2-D* image data of the SERS spectra, we cropped it to the central region of 224 × 224 to match the input requirements of the ResNet model. Subsequently, the cropped data was fed into a pre-trained ResNet50 model to obtain features of the spectral images. Finally, feature fusion by concatenating 1-D and 2-D features using matrix concatenation, which was used for subsequent spectral classification process was performed.

Transformer classification method

The core of the AL disease classification and diagnosis platform was based on the spectral recognition and classification component of the Transformer model. In this regard, six different types of classification were achieved, including non-leukemia or AL, AML or

CellPress
OPEN ACCESS

ALL, B-ALL or T-ALL, various subtypes of AML, B-ALL with or without genetic abnormalities, and BCR/ABL fusion gene positive or negative. The network model consisted of multiple components, including an Embedding layer, a Multi-Head Attention layer, a Multi-Layer Perceptron (MLP) which included two Linear layers and two Dropout layers, as well as a Fully Connected layer (FC). First, the fused features were embedded through an Embedding layer, transforming the data into fixed-dimensional vectors. They were then inputted into the Multi-Head Attention, which was the core of the classification model, allowing the model to simultaneously attend to information from different positions. Within the Multi-Head Attention, the batch size of the input data was first obtained, followed by the conversion of the input data into query (q), key (k), and value (v) vectors through linear layers. These vectors were respectively used to calculate attention scores, completed by performing matrix multiplication between the query vectors and key vectors, followed by normalization to obtain attention weights. These weights were used to weight the sum of the value vectors, with a dropout layer added to prevent overfitting, and the dropout rate was set to 0.1. Finally, the weighted value vectors were processed through normalization and linear layers, followed by addition to the original input data, yielding the output of the Multi-Head Attention.

Next, the data processed through the Multi-Head Attention was subjected to adaptive average pooling to reduce dimensions and extract important features. The pooled data then entered the MLP module, which comprised two linear layers, each followed by a dropout layer and activated using the ReLU function. Subsequently, the data passed through an FC layer to transform it into the output dimension. For the binary classifications, a sigmoid activation function was used to convert the output into probability values ranging from 0 to 1, resulting in the final classification output. For the multi-class classifications (M1&M2, M3 and M4&M5), softmax activation was used, allowing the model to output probability values for each class, enabling the prediction of multiple class probabilities simultaneously. For the two classifications, the BCELoss function was employed as the model's loss function to optimize its parameters, while the Adam optimizer updated the model's parameters with a learning rate of 0.0001 during training. Similarly, in the multi-class classification, the CrossEntropyLoss function was used as the model's loss function, and the Adam optimizer's learning rate was also set to 0.0001.

Other DL classification methods

In addition to the aforementioned Transformer classification methods based on *1-D* and *2-D* fused data (called *1-D&2-D* transformer model). Several other types of deep learning classification methods were considered in this work, including Transformer-based classification utilizing *1-D* spectral data features (called *1-D* transformer model), convolutional neural networks based on *1-D* spectral data features (called 1-D CNN model), and convolutional neural network classification using fused *1-D* and *2-D* data (called *1-D&2-D* CNN model). The *1-D* transformer method directly inputted preprocessed SERS spectral data that had undergone Savitzky-Golay smoothing, denoising, and baseline correction, and employed a Transformer model with identical parameters as the classifier.

The 1-D CNN method also used preprocessed *1-D* SERS spectral data as input to establish a convolutional neural network classification model. The network consisted of convolutional layers, pooling layers, and fully connected layers. Convolutional layers extracted different features from the information of the previous layer and then served as input data for subsequent layers of the network. LeakyReLU was used as the activation function, which is an improved version of the ReLU activation function that effectively addresses the saturation problem in the positive range of ReLU. After each convolutional layer was a maximum pooling layer to reduce the dimension of the convolutional operation results, reduce the amount of data, improve computational efficiency, and reduce the risk of overfitting. Features obtained from SERS spectral data were input into the fully connected layer for classification, and a Dropout layer was added to randomly ignore some neurons during training to reduce the risk of overfitting. Softmax was used as the activation function to output a probability distribution. After model construction, Categorical Crossentropy was used as the loss function and Adam was used as the optimizer to predict the difference between the probability distribution and the true label distribution. Finally, the 1&2-D CNN method incorporated *2-D* SERS spectral images and used resnet50 for feature extraction, concatenating and fusing different features to serve as input for the CNN classification model. The parameters of the model remained unchanged. During the modeling process, all methods utilized 80% of spectral data as the training set, 10% as the validation set, and the remaining 10% as the testing set. Classification model was developed using Python language (version 3.8.5) combined with the Pytorch and Keras deep-learning framework.

QUANTIFICATION AND STATISTICAL ANALYSIS

We used Prism (Version 7.00) to analyze differences in model performance and to conduct correlation analysis between differential Raman spectral intensities and biochemical parameters. All model data are expressed as mean ± SD and were subjected to two-way ANOVA. Values of $p < 0.05$ were considered statistically significant. Spearman's rank correlation coefficient was used to analyze correlations between differential Raman spectral intensities and biochemical parameters.

Cell Reports Medicine, Volume *6*

Supplemental information

Rapid and sensitive acute leukemia classification and diagnosis platform using deep learning-assisted SERS detection

Dongjie Zhang, Zhaoyang Cheng, Yali Song, Huandi Li, Lin Shi, Nan Wang, Yingwen Peng, Renan Chen, Nianzheng Sun, Min Han, Fengjiao Hu, Chuntao Zong, Rui Zhang, Si Chen, Conghui Zhu, Xiaoli Zhang, Xiaobo Li, Xiaopeng Ma, Changbei Shi, Xiaofei Zhang, Rui Liu, Ziqi Ren, Lin Wang, Qi Zeng, Tingting Zeng, and Xueli Chen

Supplemental Information

Rapid and sensitive acute leukemia classification and diagnosis platform using deep learning-assisted SERS detection

Dongjie Zhang, Zhaoyang Cheng, Yali Song, Huandi Li, Lin Shi, Nan Wang, Yingwen Peng, Renan Chen, Nianzheng Sun, Min Han, Fengjiao Hu, Chuntao Zong, Rui Zhang, Si Chen, Conghui Zhu, Xiaoli Zhang, Xiaobo Li, Xiaopeng Ma, Changbei Shi, Xiaofei Zhang, Rui Liu, Ziqi Ren, Lin Wang, Qi Zeng, Tingting Zeng, and Xueli Chen

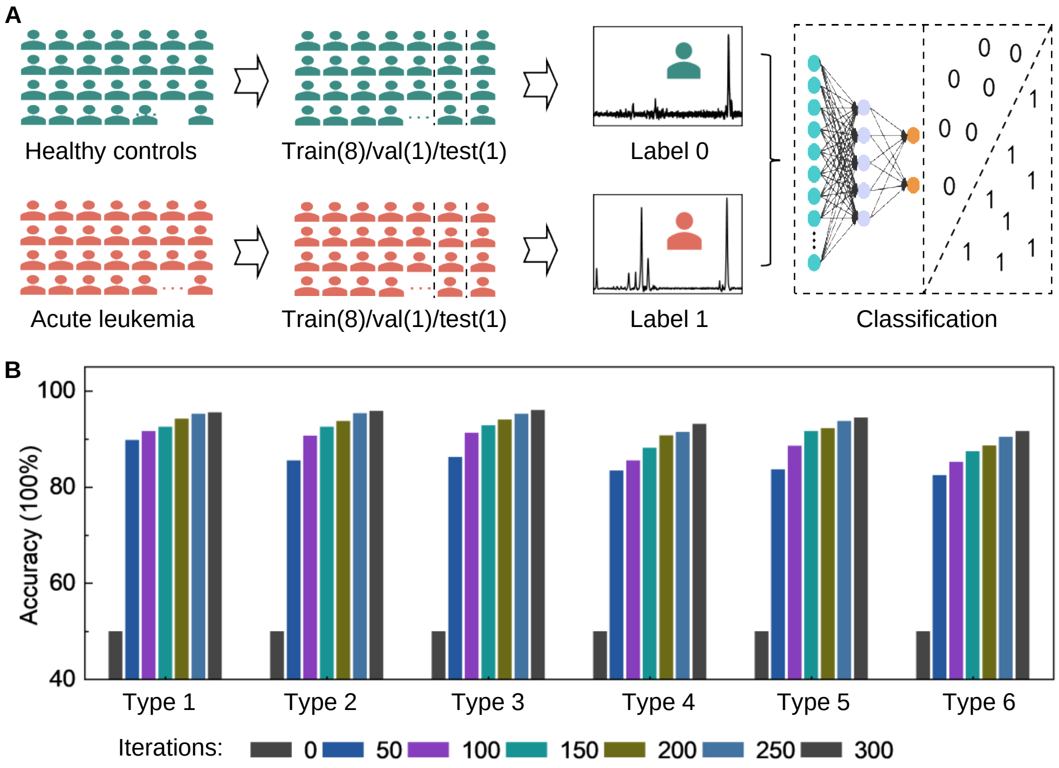

Figure S1. The strategy of dataset partitioning and classification model optimization, related to Figure 1. **(A)** The partitioning of training set and test set for binary classification. **(B)** Validation accuracy based on different iterative training of transformer algorithm with various classifications: healthy control or AL (type1), AML or ALL (type2), AML subtypes (type3), B-ALL or T-ALL (type4), B-ALL chromosome normal or abnormal (type5), and BCR/ABL1 fusion gene positive or negative (type6).

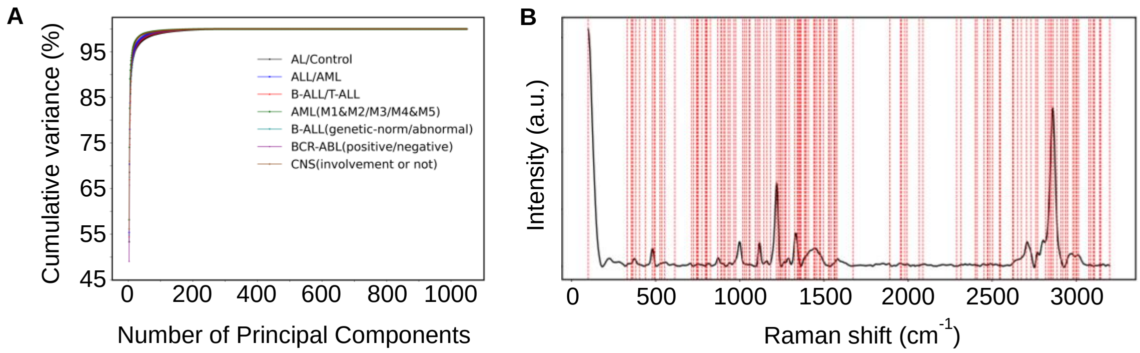

Figure S2. The extraction of spectral features used for classification, related to Figure 1. (A) the relationship between cumulative variance and the number of principal components. **(B)** visualization of the extracted key features on the original spectrum.

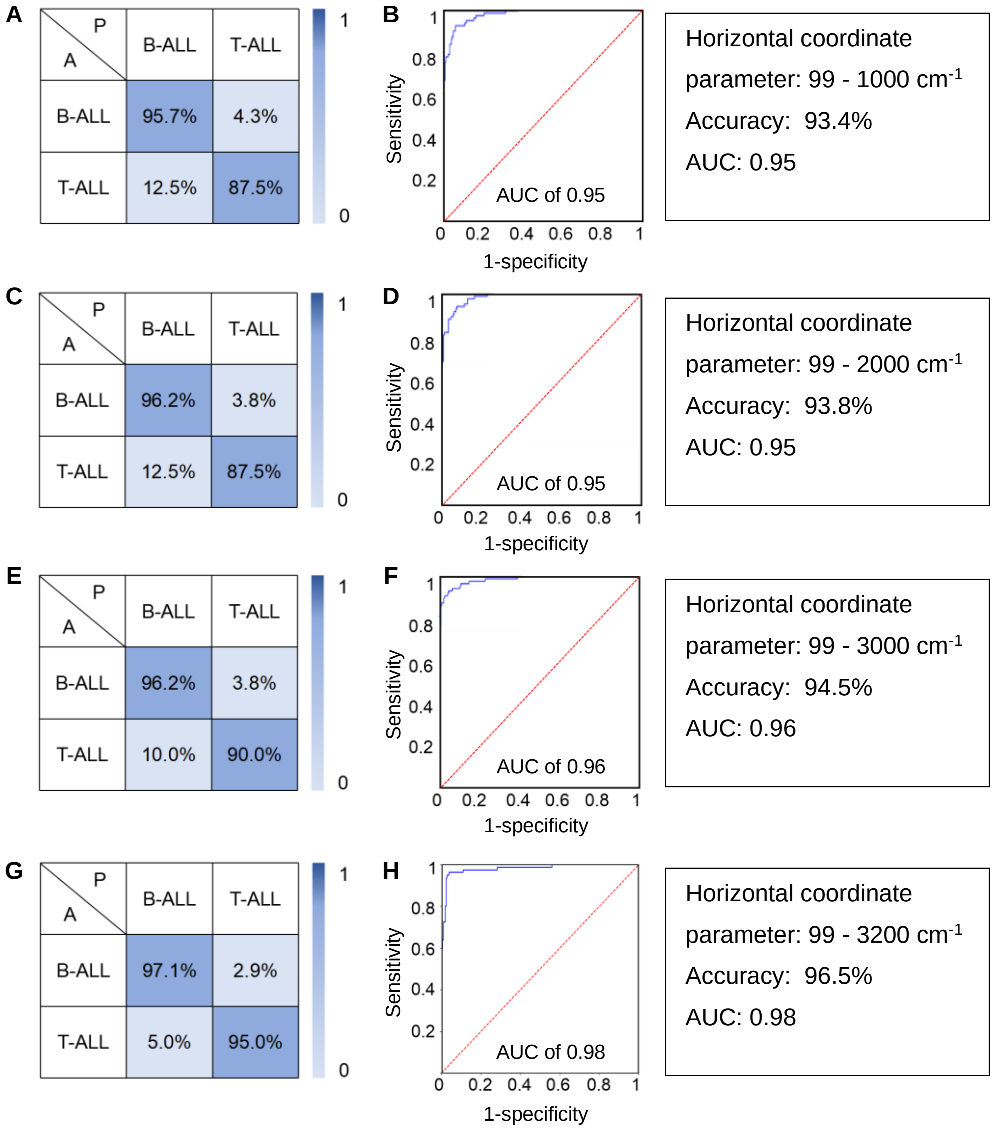

Figure S3. The effects of the selected horizontal coordinate parameters on classification results, related to Figure 1. For the binary classification of B-ALL and T-ALL, the range of the horizontal coordinate for 2-D images were: **(A-B)** 99-1000, **(C-D)** 99-2000, **(E-F)** 99-3000, and **(G-H)** 99-3200 cm^{-1}, respectively. The confusion matrix and ROCs were used to evaluate the classification performance.

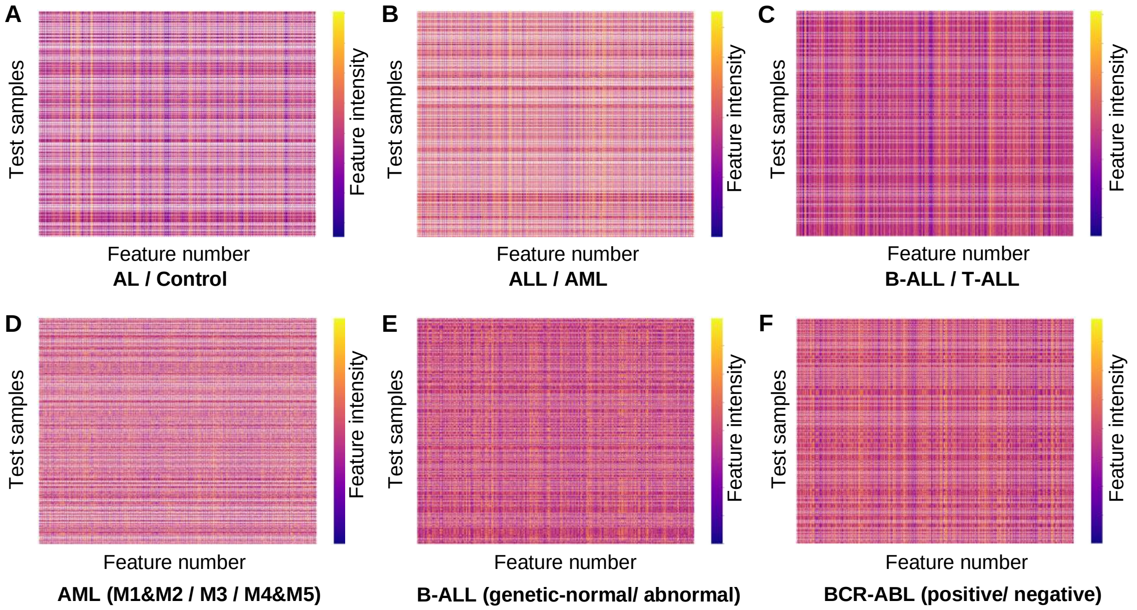

Figure S4. The visualization attention maps for various classifications, related to Figure 1. (A) AL / Control. **(B)** ALL / AML. **(C)** B-ALL / T-ALL. **(D)** AML (M1&M2 / M3 / M4&M5). **(E)** B-ALL (genetic-normal/ abnormal). **(F)** BCR-ABL fusion gene positive / negative.

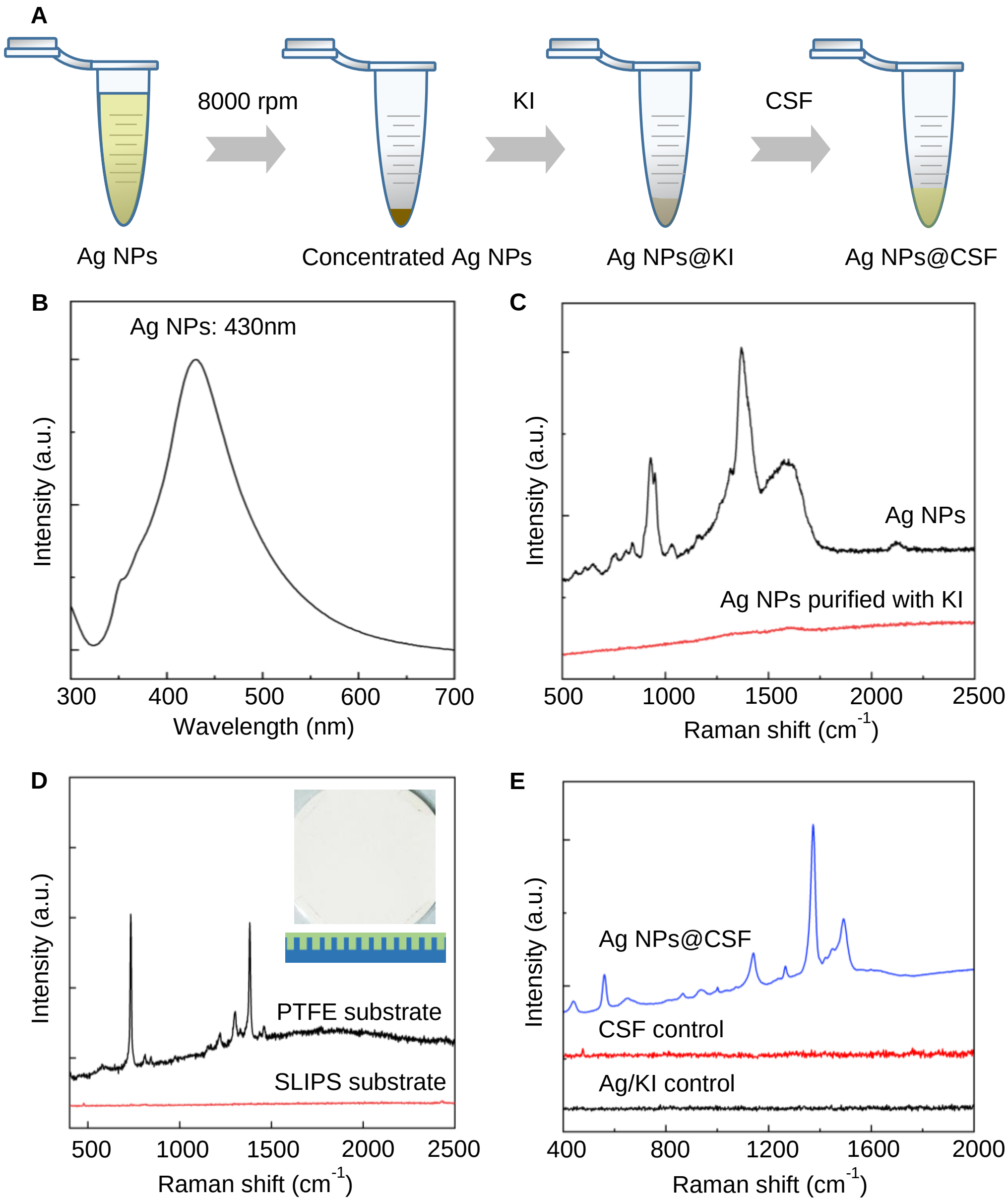

Figure S5. Characterizations of SERS enhanced substrates, related to Figure 2. (A) Schematic of sample preparation process. Firstly, concentrated Ag NPs with 50 nm size were purified with KI. Then, co-incubation process of Ag NPs and clinical CSF was taken for 10 minutes. **(B)** UV-vis absorption spectra of Ag NPs. **(C)** SERS spectra of Ag NPs before and after purified with KI agent. **(D)** SERS spectra of PTFE membrane and slippery liquid-infused porous surface (SLIPS) substrate. The insets show the optical image of SLIPS substrate. **(E)** SERS spectra of Ag/KI NPs control, CSF control (from AML patient), and mixture of Ag NPs and CSF.

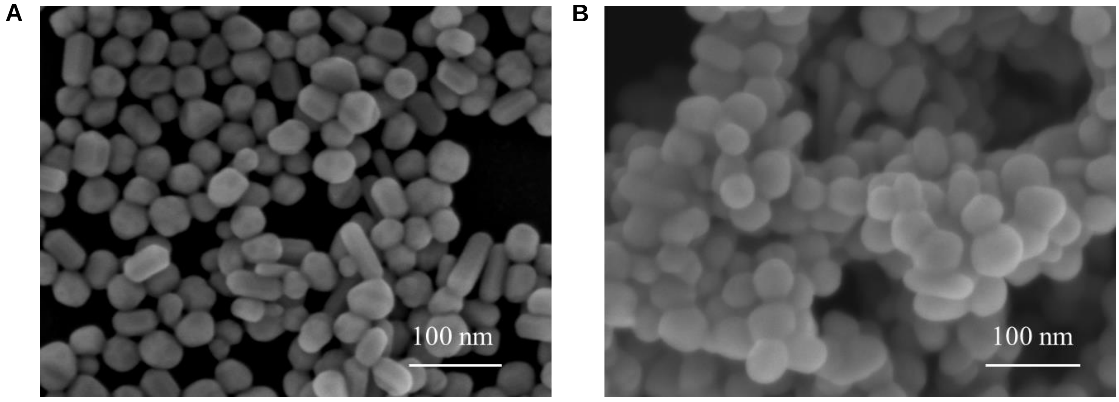

Figure S6. SEM images of colloidal Ag NPs with different aggregation station, related to Figure 2. (A) Ag NPs purified with KI. **(B)** Ag NPs mixed with CSF sample.

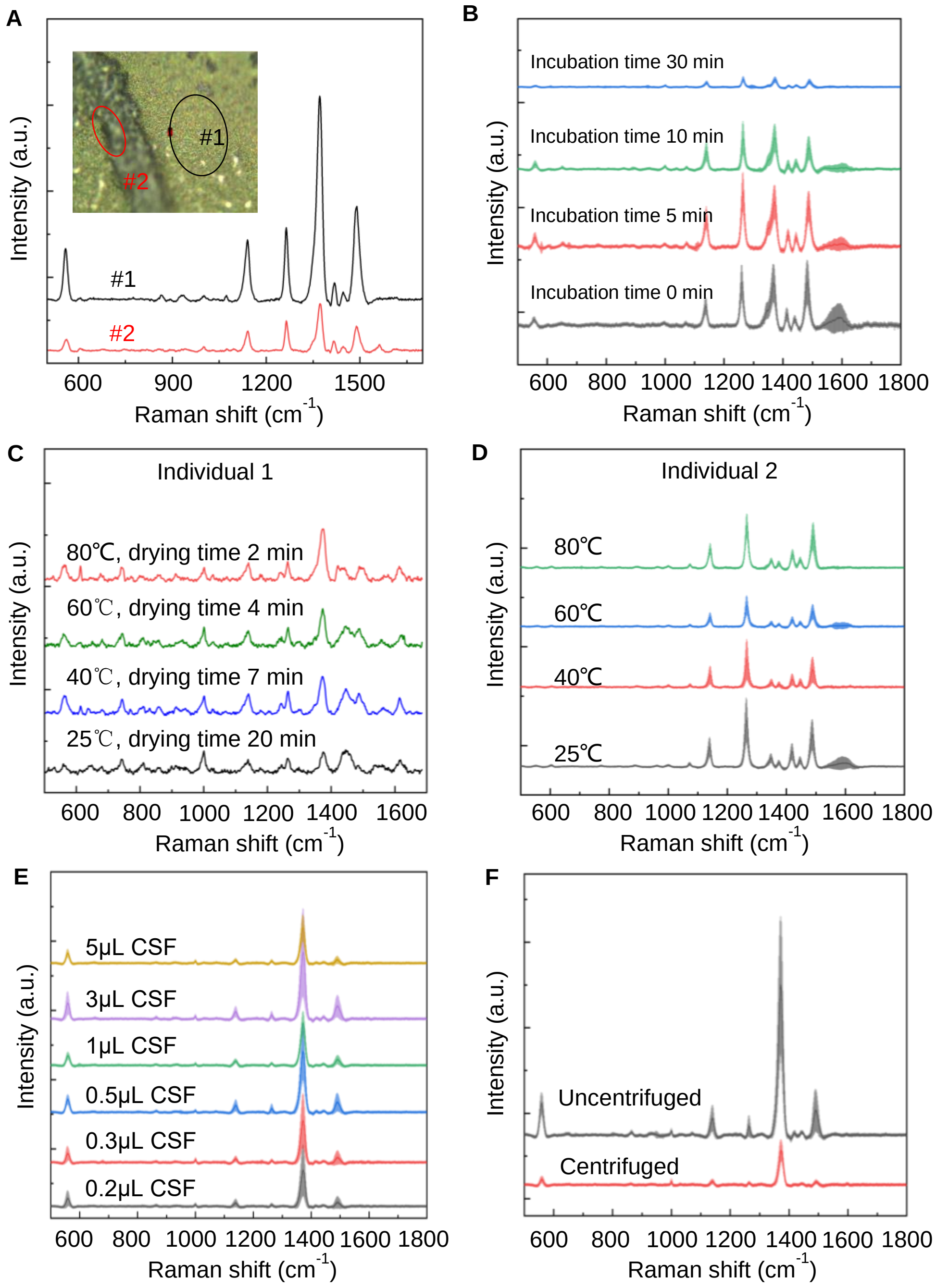

Figure S7. Optimization of SERS detection process for cerebrospinal fluid (CSF) samples, related to Figure 2. (A) Compared SERS spectra of CSF from ALL patient. Inset shows the optical image of dried SERS substrates. **(B)** SERS spectra of CSF sample with different incubation time (0, 5, 10, and 30 mins) between colloidal silver nanoparticles and CSF samples. **(C-D)** SERS spectra of CSF samples from various individuals with different evaporation temperatures. **(E)** SERS spectra of CSF samples with different volumes. **(F)** SERS spectra of CSF samples before and after the pretreatment of centrifugation.

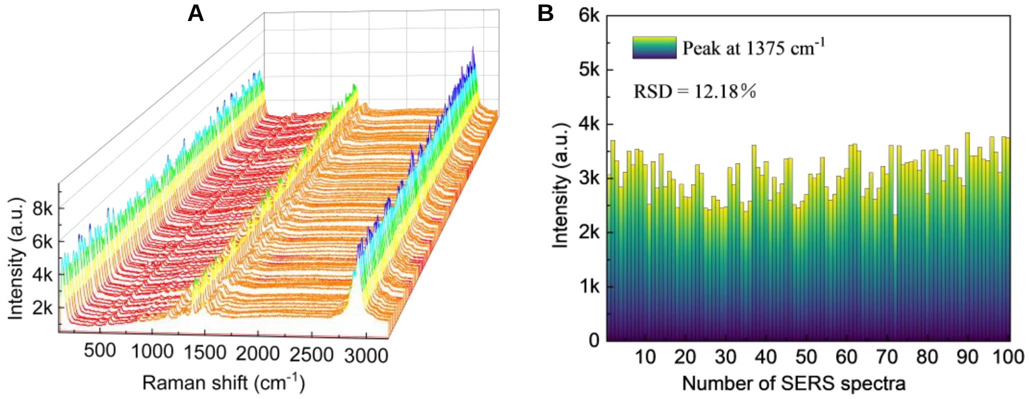

Figure S8. The performance of SERS consistency, related to Figure 2. (A) 50 of SERS spectra. **(B)** the relative standard deviation (RSD) value of characteristic band at 1375 cm^{-1}.

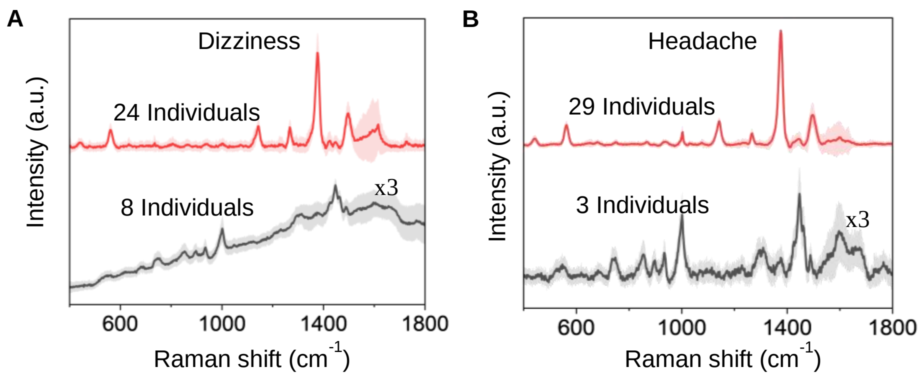

Figure S9. The compared SERS spectra of CSF samples from dizziness and headache individuals, related to Figure 2. (A) 32 individuals with dizziness. **(B)** 32 individuals with headache.

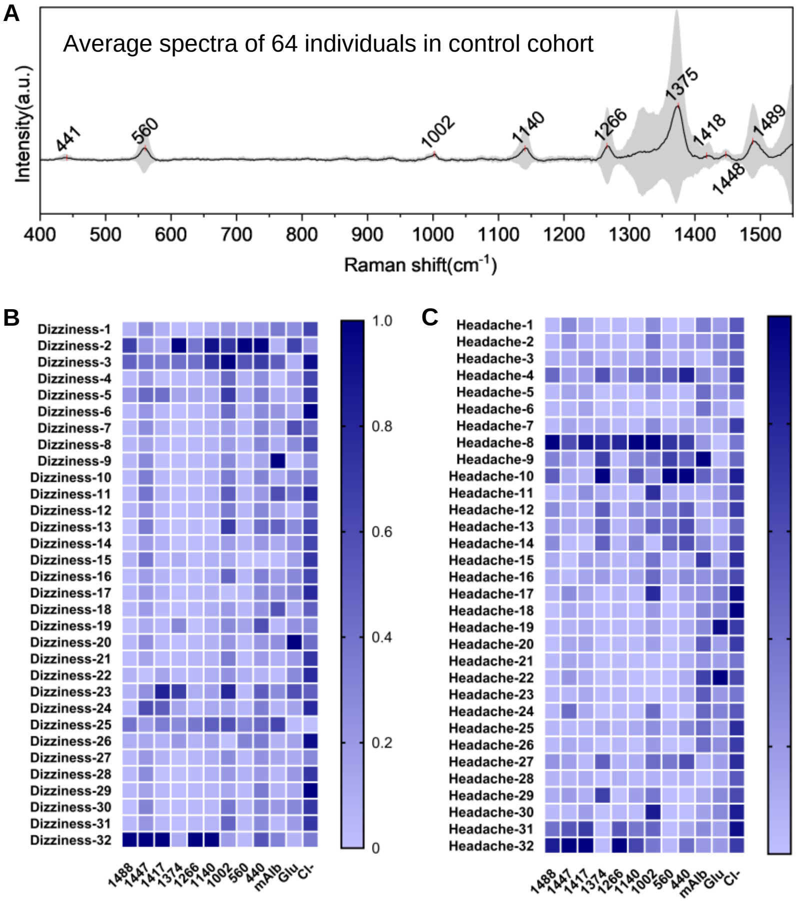

Figure S10. The individual variability within the healthy group, related to Figure 2. (A) The average SERS spectra of CSFs collected from healthy group. **(B)** The heat mappings of SERS characteristic peaks and chemical components to visualize the inter-individual variability and signal uniformity.

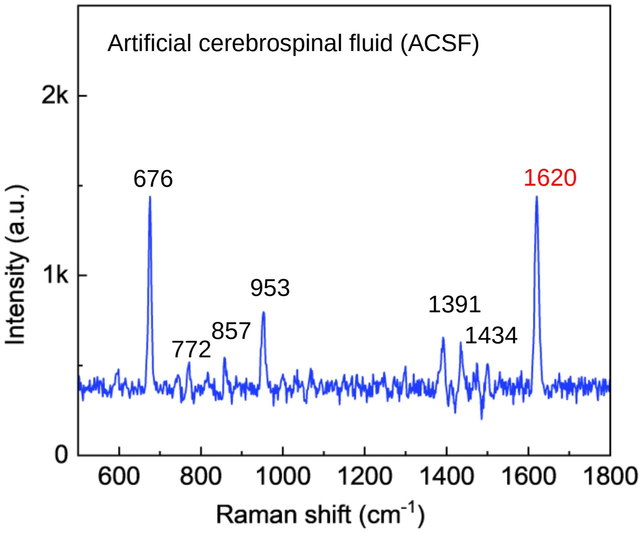

Figure S11. Typical SERS spectra of artificial cerebrospinal fluid (ACSF), related to Figure 2.

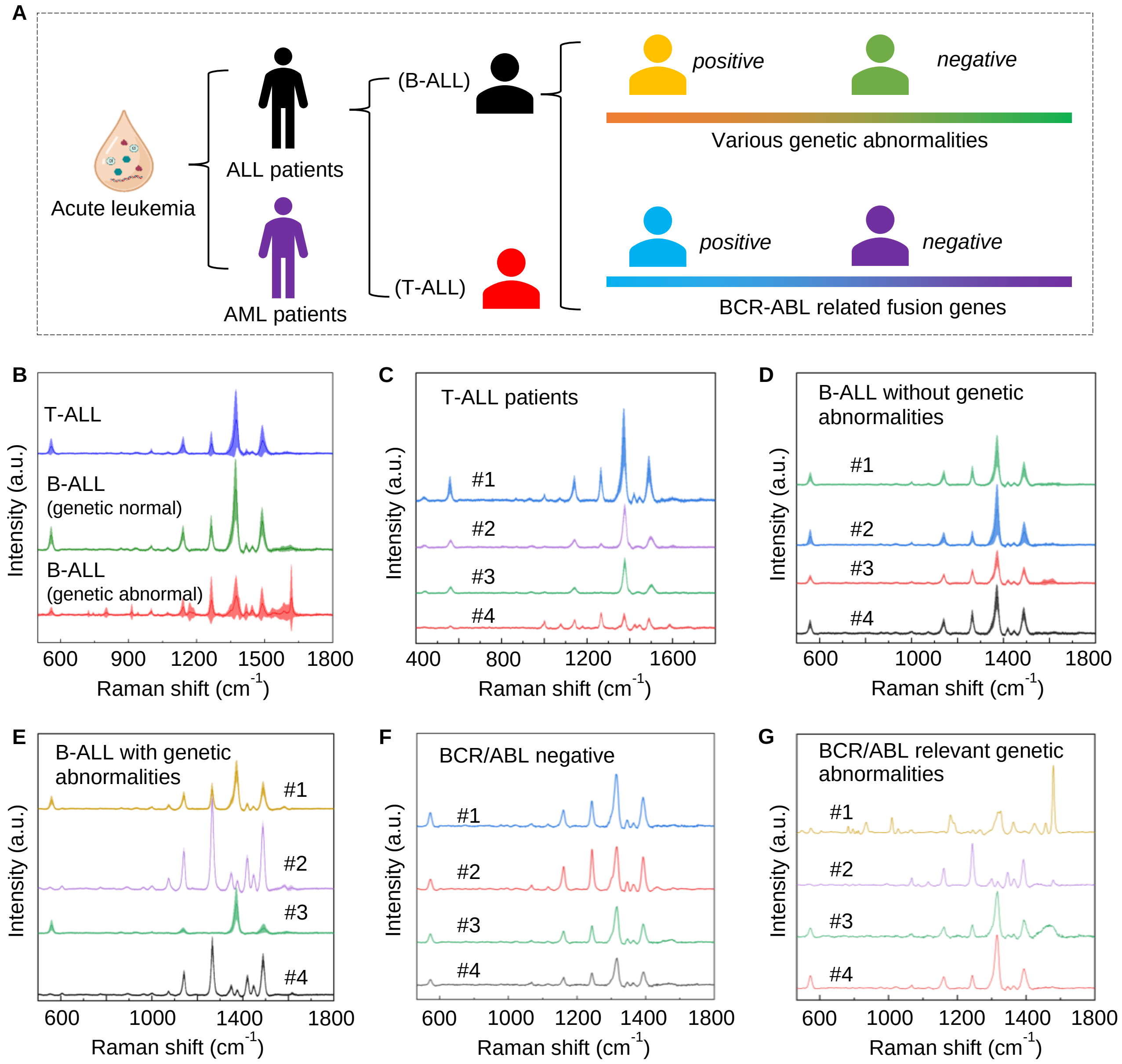

Figure S12. SERS analysis of CSF samples from various acute leukemia (AL) patients, related to Figure 3. (A) Overview of the AL patient information, i.e., AML/ALL, sub-categories of AML and ALL, and B-ALL with genetic or BCR/ABL relevant abnormalities. **(B)** SERS spectra of CSF samples collected from T-ALL and B-ALL patients. **(C-G)** Influence of individual differences on SERS spectra of CSF from ALL patients: **(C)** T-ALL patients; **(D)** B-ALL without genetic abnormalities; **(E)** B-ALL with different genetic abnormalities; **(F)** BCR/ABL negative B-ALL patients, and **(G)** B-ALL with different BCR/ABL relevant genetic abnormalities. For each CSF sample, at least 50 of SERS spectra was collected.

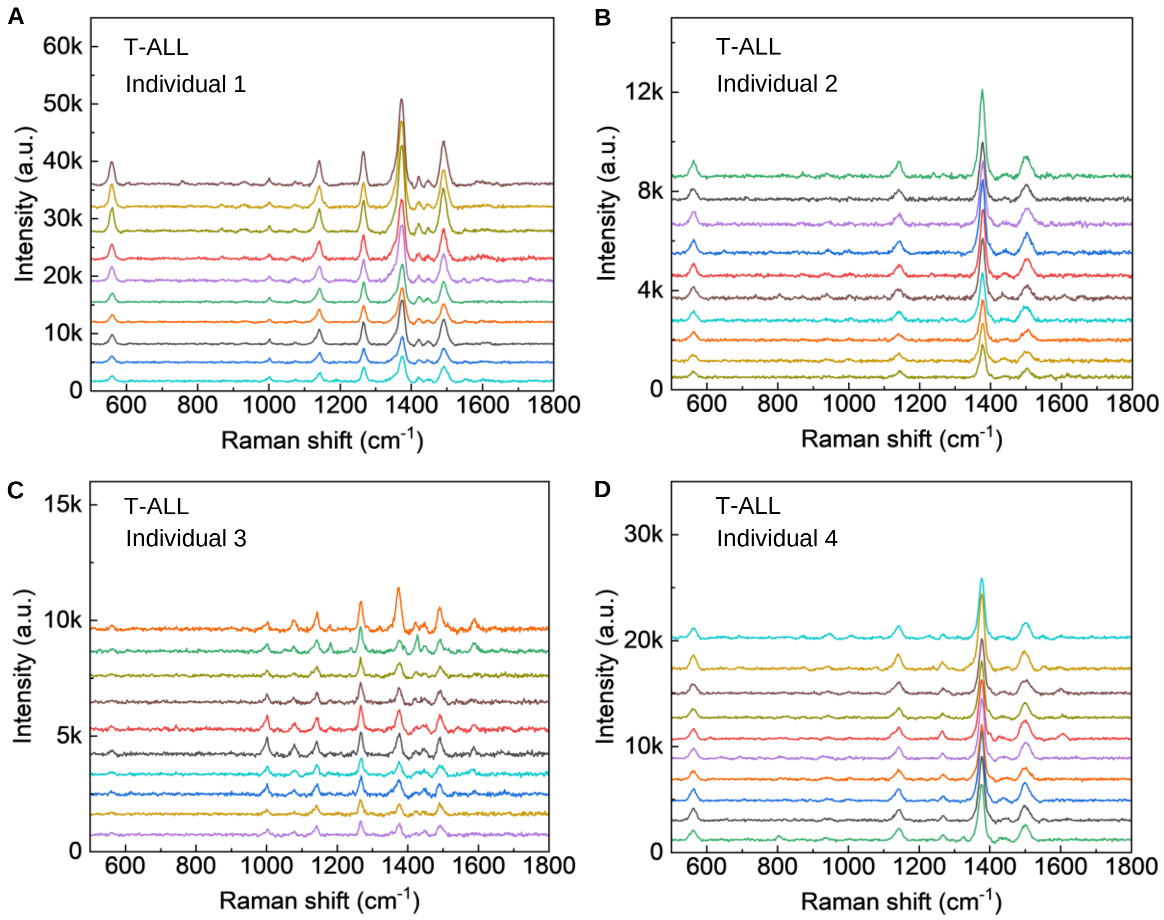

Figure S13. SERS spectra of CSF samples from different T-ALL patients, related to Figure 3.

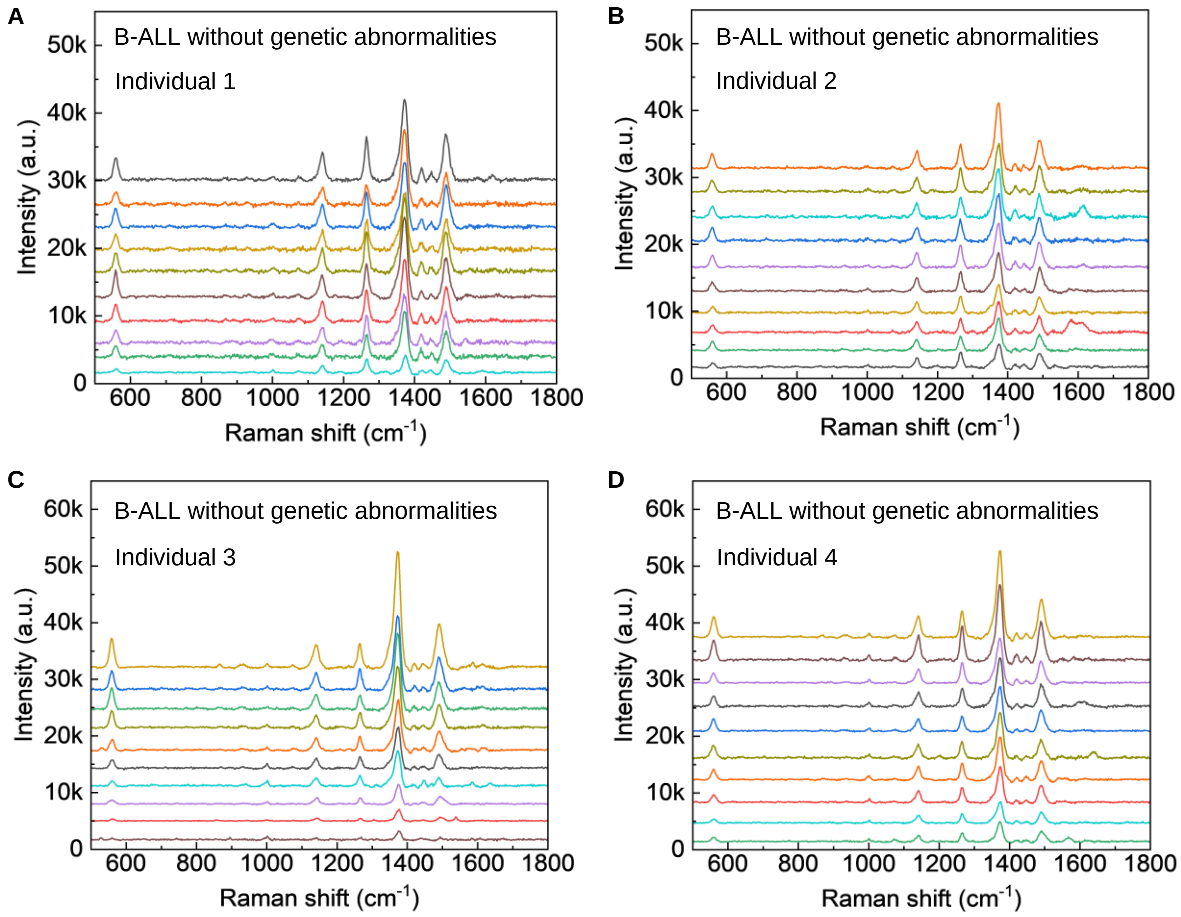

Figure S14. SERS spectra of CSF from B-ALL patients without genetic abnormalities, related to Figure 3.

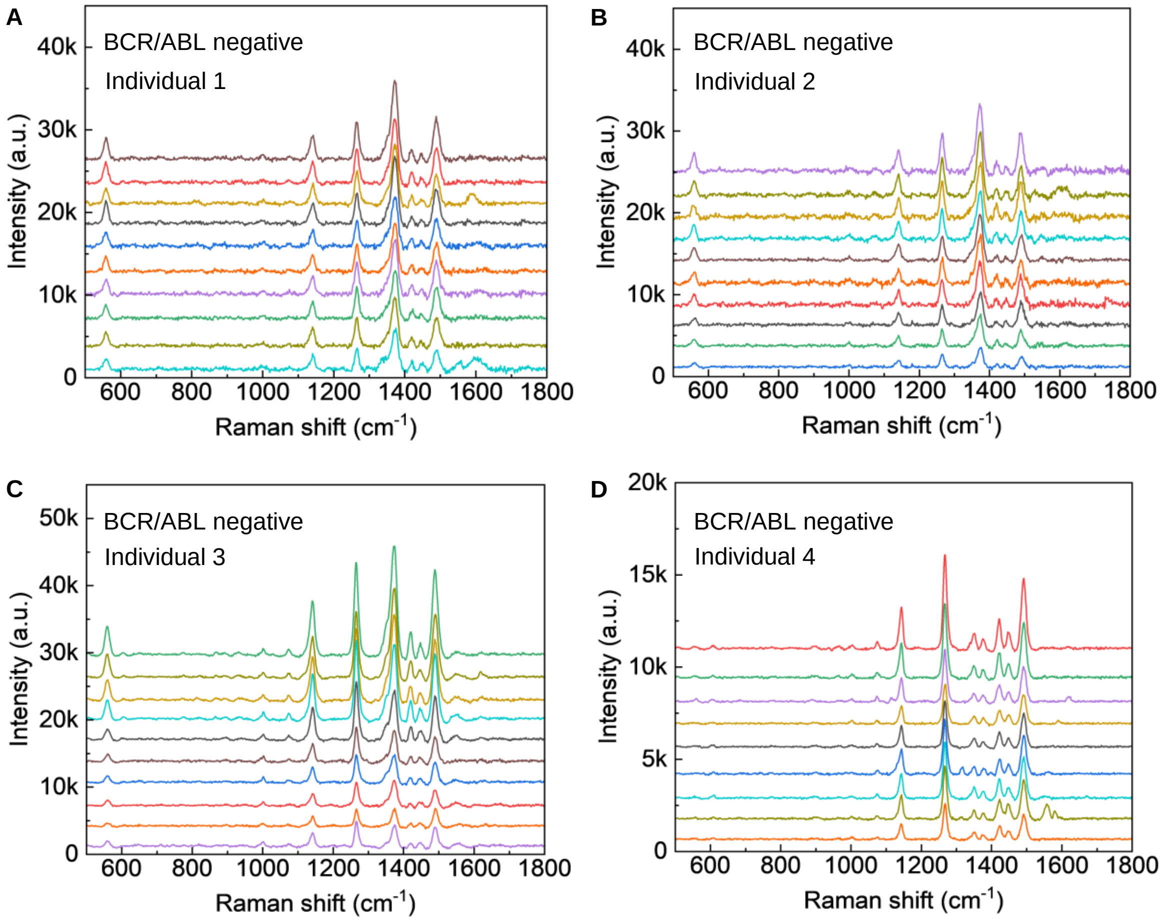

Figure S15. SERS spectra of CSF from different BCR/ABL negative ALL patients, related to Figure 3.

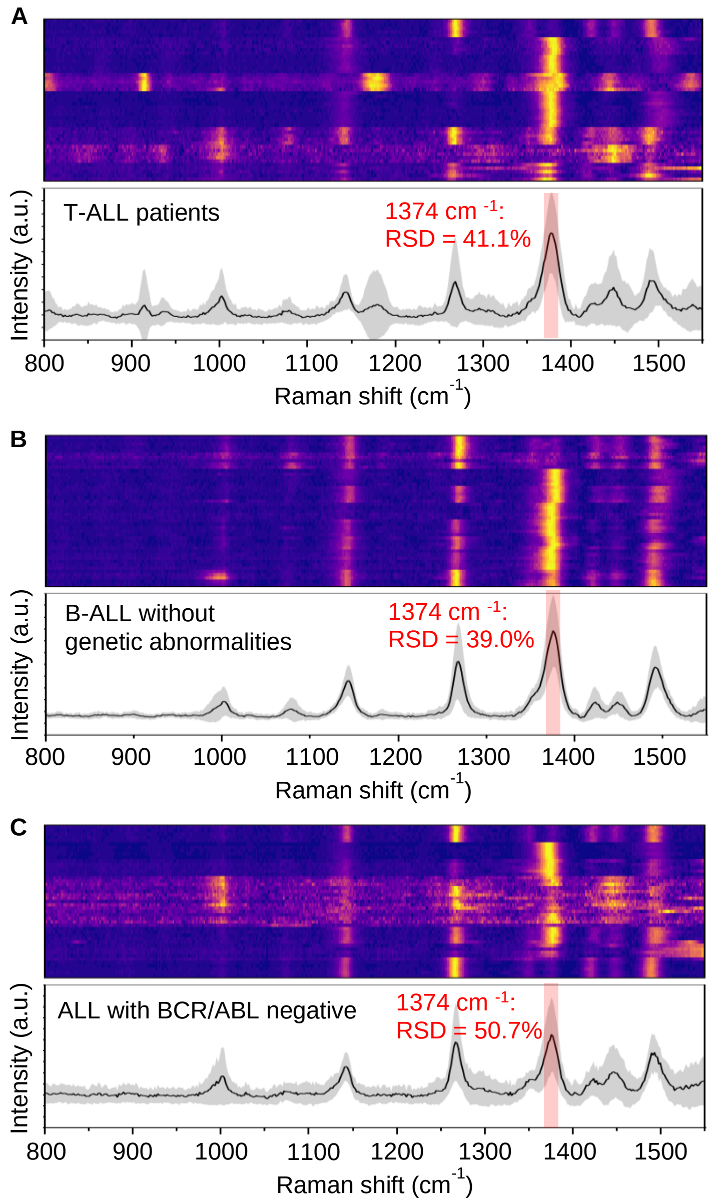

Figure S16. The average SERS spectra and corresponding heat mapping to visualize the inter-individual variability and signal uniformity, related to Figure 3. (A) SERS spectra and heat mapping of CSFs from T-ALL patients; **(B)** SERS spectra and heat mapping of CSFs from B-ALL without genetic abnormalities; **(C)** SERS spectra and heat mapping of CSFs from ALL with BCR/ABL negative.

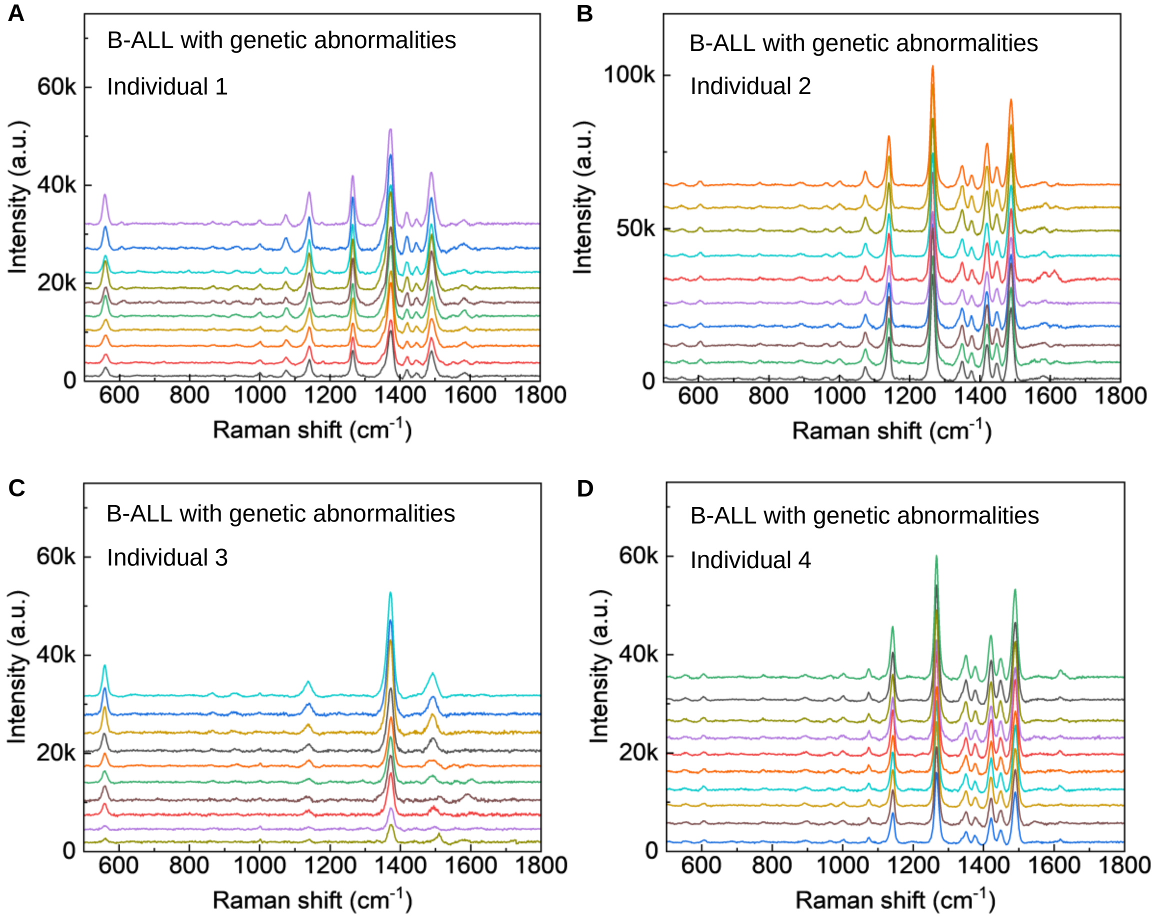

Figure S17. SERS spectra of CSFs from different B-ALL patients with genetic abnormalities, related to Figure 3.

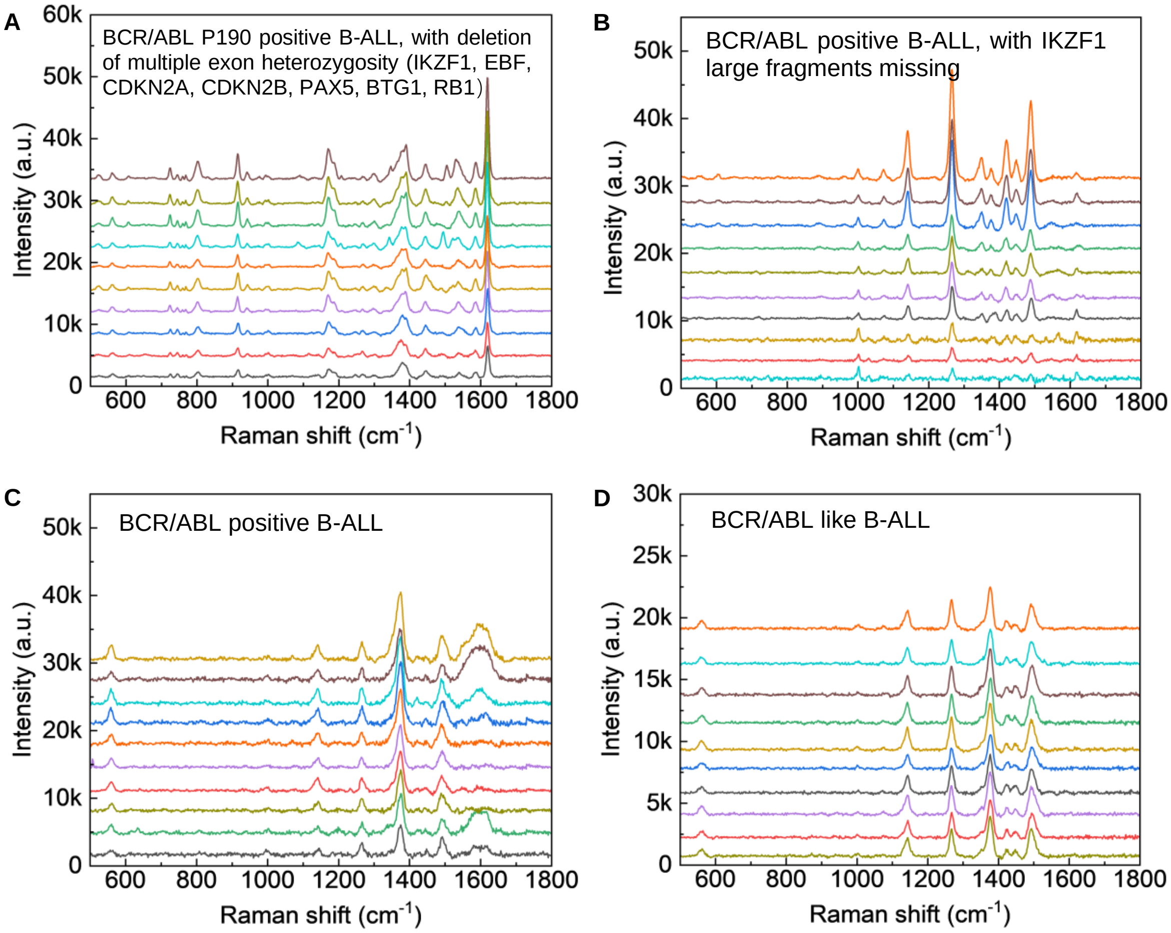

Figure S18. SERS spectra of different CSF samples from B-ALL patients with BCR/ABL relevant genetic abnormalities, related to Figure 3.

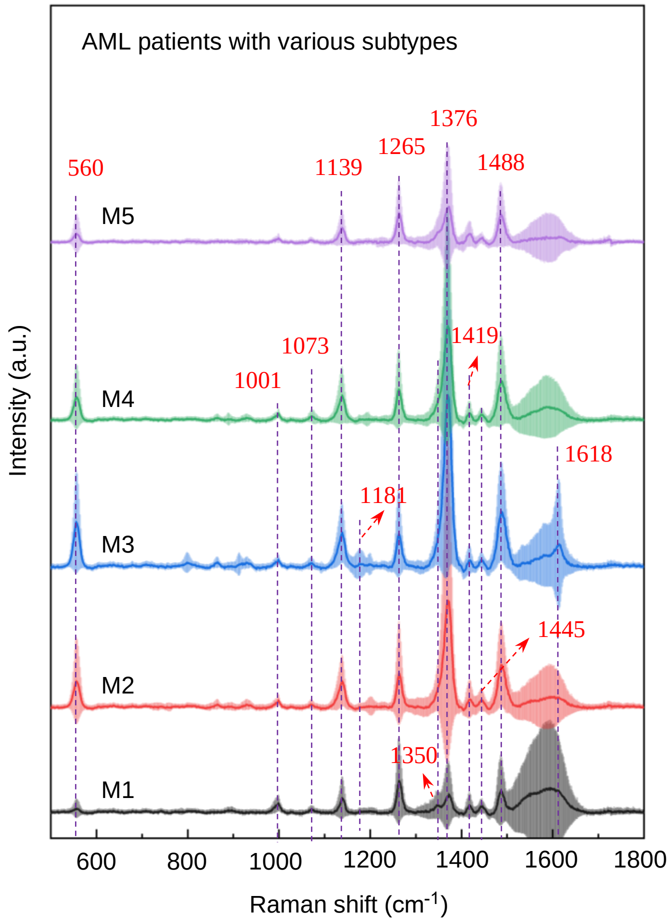

Figure S19. Compared SERS spectra of CSF from AML patients with different subtypes, related to Figure 3.

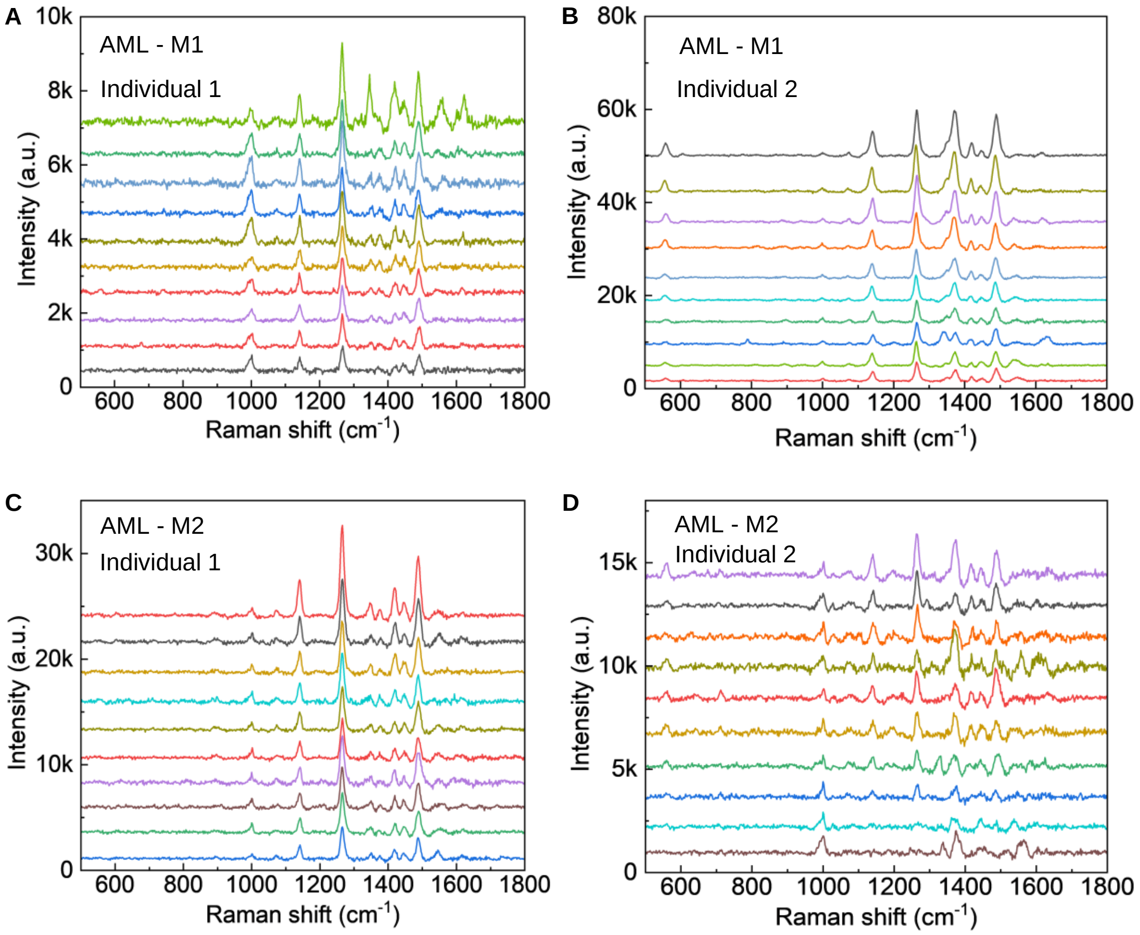

Figure S20. SERS spectra of CSF samples from acute myeloid leukemia (AML), related to Figure 3. (A-B) M1 subtype. **(C-D)** M2 subtype.

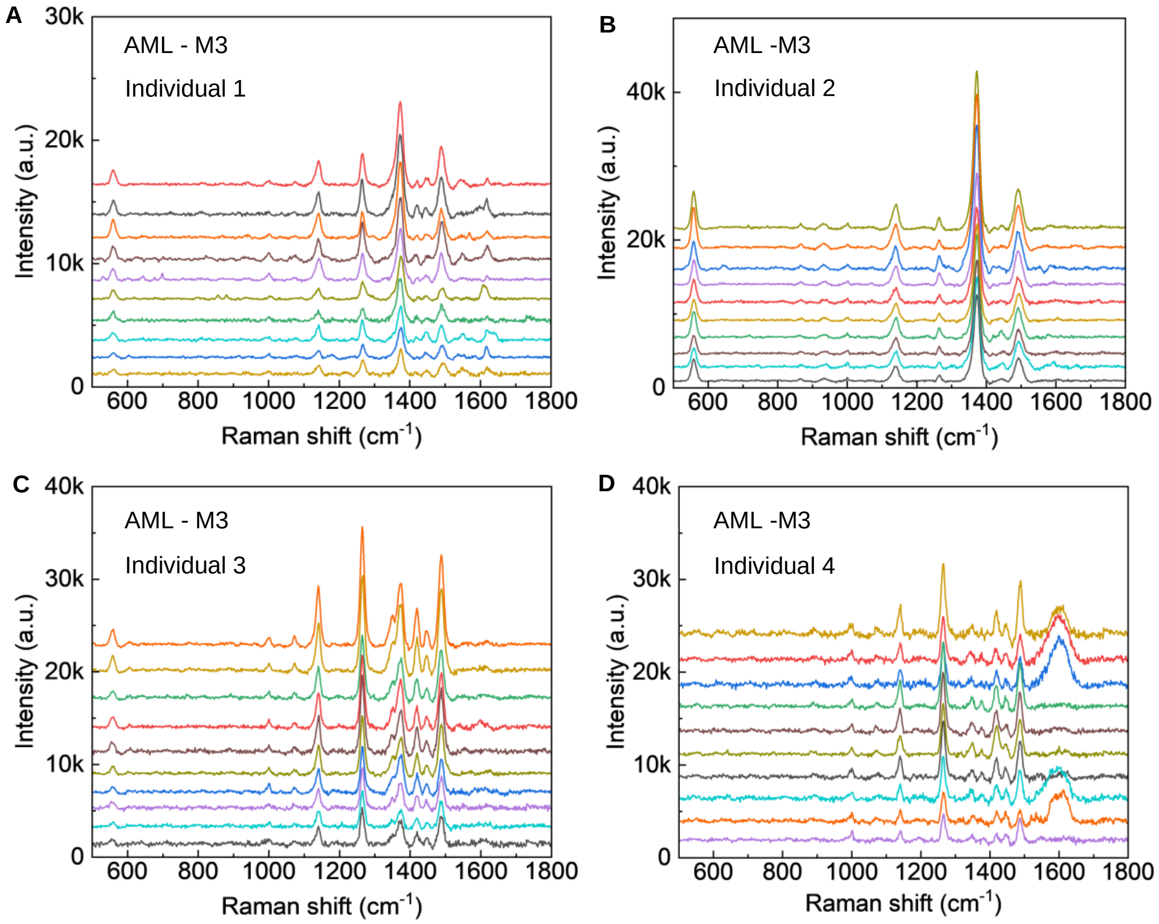

Figure S21. SERS spectra of CSF samples from different AML patients with M3 subtype, related to Figure 3.

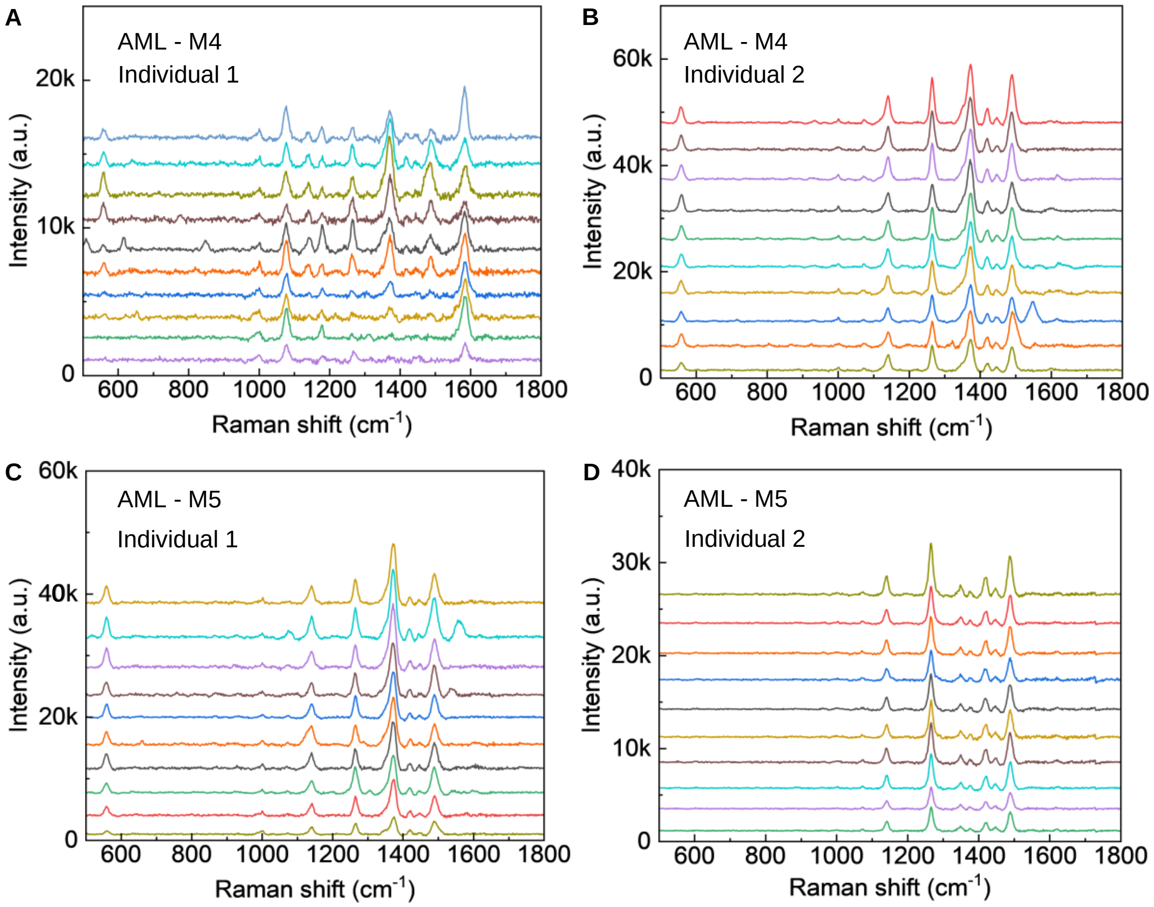

Figure S22. SERS spectra of CSF from different AML patients, related to Figure 3. (A-B) M4 subtype. **(C-D)** M5 subtype.

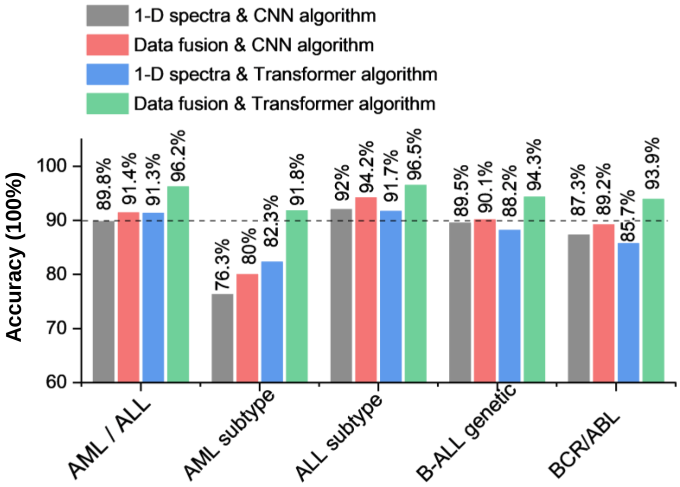

Figure S23. Compared prediction accuracy performances based on various DL models, related to Figure 3. The models include features fusions (*1-D* spectra and *2-D* image) combined with transformer, transformer based on *1-D* spectra, features fusion combined with CNN, and CNN based on *1-D spectra*.

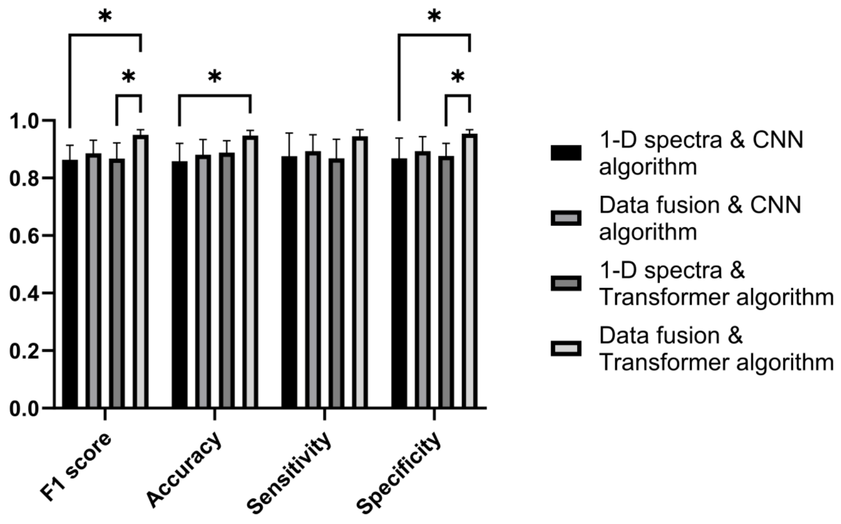

Figure S24. The significance test on the classification performance metrics obtained from different methods, related to Figure 3. The classification indexes include F1 score, accuracy, sensitivity, and specificity.

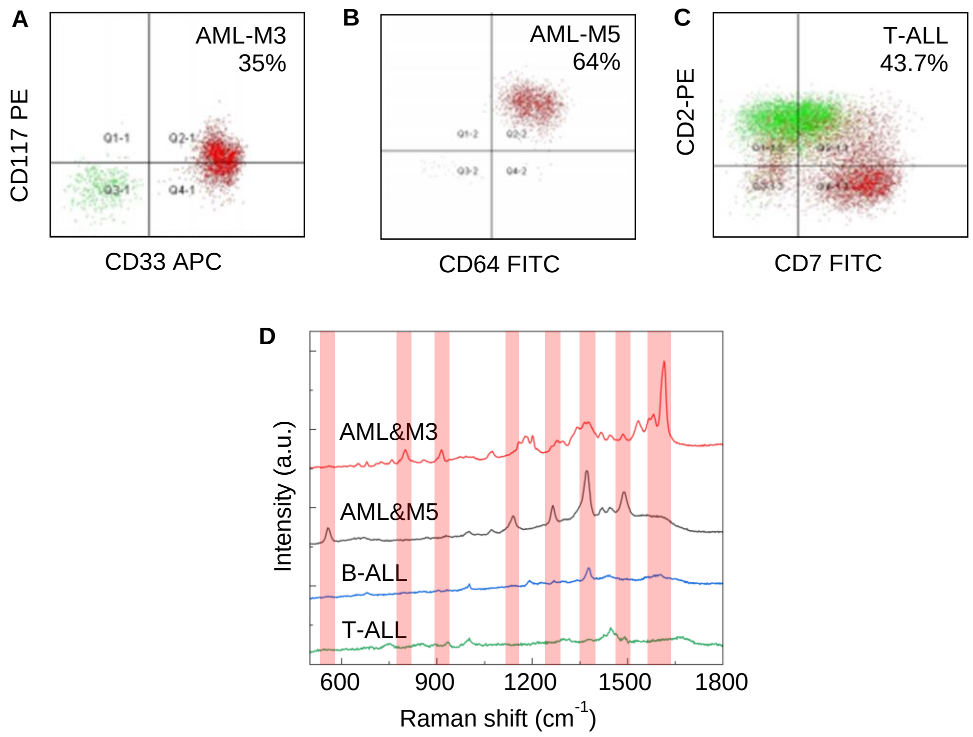

Figure S25. Flow cytometry (FCM) and related SERS analysis of different acute leukemia patients with central nervous system (CNS) involvements, related to Figure 4. (A-C) FCM results of patients with CNS leukemia (CNSL) that developed from **(A)** acute myeloid leukemia with M3 subtype (AML&M3), **(B)** acute myeloid leukemia with M5 subtype (AML&M5), and **(C)** T-cell acute lymphoblastic leukemia (T-ALL). In these figures, the red dots denote leukemia cells, while the green dots signify normal lymphocytes. **(D)** SERS spectra of CSF samples from CNSL patients developed from diverse acute leukemia (AL) subtypes, encompassing AML&M3, AML&M5, B-ALL, and T-ALL.

Table S1. The components of artificial cerebrospinal fluid (ACSF), related to Figure 2.

Components	NaCl	KCI	$NaHCO_3$	NaH_2PO_4	$MgSO_4$	$CaCl_2$	$C_6H_{12}O_6$	H_2O
Volume (μL)	100	100	100	10	10	10	10	660
Concentration (mM)	1200	30	260	124	200	200	100	/

Table S2. Information of CSF samples from healthy and acute leukemia groups, related to Figure 2.

			Healthy control cohort		Acute leukemia cohort				
					AML patients			ALL patients	
			Dizziness (32)	Headaches (32)	M1&M2 (26)	M3 (22)	M4&M5 (33)	B-ALL (52)	T-ALL (15)
Exterior features of sample	Transparency	Yes	31	32	25	22	32	52	15
		No	1	0	1	0	1	0	0
	Clot	Yes	0	0	0	0	0	0	0
		No	32	32	26	22	33	52	15
Main biochemical components	Total protein (g/L)	0-0.3	1	11	11	5	6	18	3
		0.3-0.6	18	20	12	13	22	27	10
		＞0.6	13	1	3	4	5	7	2
		Mean value (90% range)	0.64±0.12 (0.30-1.83)	0.36±0.05 (0.17-0.79)	0.41±0.11 (0.15-1.42)	0.41±0.07 (0.18-0.86)	0.43±0.06 (0.12-1.18)	0.39±0.05 (0.21-1.15)	0.48±0.12 (0.25-1.17)
	Chloride (mmol/L)	＜120	2	1	2	0	0	1	0
		120-130	28	28	24	20	30	46	13
		＞130	2	3	0	2	3	5	2
		Mean value (90% range)	124.40±1.12 (116-130)	126.09±0.89 (118-131)	125.21±1.01 (119-130)	127.41±1.20 (123-135)	126.25±1.07 (121-133)	126.40±0.82 (118-134)	124.93±1.90 (120-133)
	Glucose (mmol/L)	2-3	5	3	7	3	10	14	4
		3-4	22	25	17	19	22	32	6
		＞4	5	4	2	0	1	6	5
		Mean value (90% range)	3.62±0.30 (2.61-6.72)	3.71±0.34 (2.61-7.30)	3.43±0.25 (2.50-5.48)	3.27±0.11 (2.69-3.91)	3.20±0.11 (2.70-4.26)	3.37±0.15 (2.18-4.87)	3.71±0.44 (2.57-5.31)
Number of cells containing in CSF	0-10		27	23	9	12	17	21	4
	10-50		2	1	0	1	0	0	0
	＞50		3	8	7	4	6	3	2

Table S3. Peak assignments of the potential biochemical components in CSF, related to Figure 2, Figure 4, and Figure 5.

Peaks (cm^{-1})	Vibrational mode	Possible assignments	Ref.
560	/	adenine, glycine	1
723	C-H bending	nucleic acid, adenine, coenzyme A;	2, 3, 4
744	Ring breathing vibrations	nucleic acid, thymine	2
803	C-C-O stretching vibration	glutathione, L-serine, thymine	3, 4, 5, 6
914	Ring breathing vibrations	glucose, phenylalanine	7
1001	C-C stretching; C−C ring breathing	amino acid; phenylalanine, lactose	2, 3, 4, 7
1028	C-H in plane deformation	phenylalanine	6, 8
1073	C-N stretching, skeletal C-C stretching	protein; collagen; L-glutamate,	2, 3, 4
1139	C-H stretching; C-C stretching; C-N stretching	nucleic acid; adenine	2, 3, 4
1169	C–N stretching	nucleic acid	2, 7
1181	C-C symmetric stretching; C-O symmetric stretching	cytosine; adenine; guanine	8
1265	C−H deformation; C-N stretching	nucleic acid	8
1350	C-N stretching	proline	8
1373	Ring breathing	thymine, adenine, guanine	5, 8
1376	COO^- symmetric stretching	nucleic acids	7, 9
1419	C=C stretching	DNA, RNA	8
1445	- CH_2 deformation; $\delta(CH_2)$	malic acid; L-phenylalanine	3, 5, 7
1488	C-H bending vibrations	proteins, collagen	7, 9
1537	Amide carbonyl group vibrations, aromatic hydrogens	L-Glutamate, glutathione	8
1583	C=C bending; ring stretch	nucleic acids; tryptophan	5, 7, 9
1618	C=C in-plane bending	tyrosine; amino acid	2, 4, 6

Table S4. Characteristics of the B-ALL patients with BCR/ABL fusion genetic abnormalities, related to Figure 3.

B-ALL	Age in years	Gender	Diagnosis	Other genetic abnormalities
Individual-1	32	Male	BCR/ABL (Ph+)	None
Individual-2	34	Female	BCR/ABL (Ph+), p190	Heterozygous deletions of multiple gene exons in IKZF1, EBF, CDKN2A, CDKN2B, PAX5, BTG1, RB1.
Individual-3	67	Female	BCR/ABL (Ph+)	T315I gene mutation
Individual-4	44	Male	BCR/ABL (Ph+), p210	Large fragment deletions of IKZF1, CDKN2A, CDKN2B, ETV6 and PAX5, accompanied by ABL kinase T315I mutation
Individual-5	31	Female	Ph-like ALL	None
Individual-6	28	Male	Ph-like ALL	Large-fragment deletion mutation in IKZF1 gene.
Individual-7	34	Female	BCR/ABL (Ph+), p190	Heterozygous deletions of multiple gene exons in IKZF1, EBF, CDKN2A, CDKN2B, PAX5, BTG1, RB1.
Individual-8	32	Female	Ph-like ALL	None
Individual-9	65	Male	Ph-like ALL	None
Individual-10	34	Female	BCR/ABL (Ph+)	Heterozygous deletions in exons 1 to 8 of IKZF1 gene.
Individual-11	32	Female	Ph-like ALL	None
Individual-12	32	Male	BCR/ABL (Ph+)	None
Individual-13	32	Male	BCR/ABL (Ph+)	None
Individual-14	36	Female	BCR/ABL (Ph+)	Large fragment deletions of IKZF1 gene.
Individual-15	32	Male	BCR/ABL (Ph+), p210	Heterozygous deletions in exons 1-8 of IKZF1 gene, exons 2 and 4 of CDKN2A gene, exon 2 of CDKN2B gene, and exons 1, 2, 5, 6, 7, 8, and 10 of PAX5 gene.
Individual-16	34	Female	BCR/ABL (Ph+), p190	Heterozygous deletions in exons 1-8 of IKZF1 gene, exons 2 and 4 of CDKN2A gene, exon 2 of CDKN2B gene, and exons 1, 2, 5, 6, 7, 8, and 10 of PAX5 gene.
Individual-17	26	Female	BCR/ABL (Ph+)	46, XX, t (9,22)
Individual-18	21	Male	BCR/ABL (Ph+)	IKZF1、FAT1、FAT3 genetic positive, and heterozygous deletions in exons 4 to 7 of IKZF1 gene.
Individual-19	44	Female	BCR/ABL (Ph+)	None
Individual-20	33	Female	BCR/ABL (Ph+), p190	Heterozygous deletions in exons 4 to 7 of IKZF1 gene.
Individual-21	58	Male	BCR/ABL (Ph+), p190	t (9;22) (q34;q11), t (8;14) (q11;q32)
Individual-22	56	Female	BCR/ABL (Ph+)	45,XX,der(4)t(1;4)(q21;q35),t(9;22)(q34;q11),add(18)(p11.3)； ABL kinase T315I mutation, and heterozygous deletions in exons 4 to 7 of IKZF1 gene.
Individual-23	55	Male	BCR/ABL (Ph+)	t(9;22) (q34;q11), +X, del(2)(q23), add(4)(p16); Heterozygous deletions inexons of IKZF1, CDKN2A, BTG1, RB1 gene.
Individual-24	19	Male	Ph-like ALL	PDGFRB、CSF1R gene rearrangement; heterozygous deletions in exons 4-8 of IKZF1 gene, exons 2, 5, 6, and 7 of PAX5 gene, and exon 16 of EBF1 gene.
Individual-25	22	Female	Ph-like ALL	SSBP2/CSF1R fusion genetic positive; heterozygous deletions of multiple exons of IKZF1 gene
Individual-26	55	Male	BCR/ABL (Ph+)	t(9;22) (q34;q11), +X, del(2)(q23), add(4)(p16); heterozygous deletions in exons 4-7 of IKZF1 and CDKN2A gene; exons 6, 14, 19, and 26 of RB1 gene; and allelic loss in exon 2 of BTG1 gene.

Table S5. Prediction performance for external set validation, related to Figure 4.

AML/ALL (Binary classification)				AML subtypes (Trinary classification)						ALL subtypes (Binary classification)				B-ALL genetic (Binary classification)			
AML (n=15)		ALL (n=15)		M1/M2 (n=10)		M3 (n=10)		M4/M5 (n=10)		B-ALL (n=15)		T-ALL (n=15)		Normal (n=15)		Abnormal (n=15)	
No.	Feedback	No.	Feedback	No.	Feedback	No.	Feedback	No.	Feedback	No.	Feedback	No.	Feedback	No.	Feedback	No.	Feedback
#1	√	#16	√	#31	√	#41	√	#51	√	#61	√	#76	√	#91	√	#106	√
#2	×	#17	√	#32	√	#42	√	#52	√	#62	√	#77	√	#92	√	#107	√
#3	√	#18	√	#33	×	#43	√	#53	√	#63	√	#78	×	#93	√	#108	√
#4	√	#19	√	#34	√	#44	√	#54	√	#64	√	#79	√	#94	√	#109	√
#5	√	#20	×	#35	×	#45	×	#55	√	#65	√	#80	√	#95	√	#110	√
#6	√	#21	√	#36	√	#46	√	#56	√	#66	√	#81	√	#96	√	#111	√
#7	√	#22	√	#37	√	#47	√	#57	√	#67	√	#82	√	#97	√	#112	√
#8	√	#23	√	#38	√	#48	√	#58	√	#68	√	#83	√	#98	√	#113	√
#9	√	#24	√	#39	√	#49	√	#59	√	#69	√	#84	√	#99	√	#114	√
#10	√	#25	√	#40	√	#50	√	#60	√	#70	√	#85	√	#100	×	#115	√
#11	√	#26	√							#71	√	#86	√	#101	√	#116	√
#12	√	#27	√							#72	√	#87	√	#102	√	#117	×
#13	√	#28	√							#73	√	#88	√	#103	√	#118	√
#14	√	#29	√							#74	√	#89	√	#104	√	#119	√
#15	√	#30	√							#75	√	#90	√	#105	√	#120	√

Table S6. Characteristics of the test datasets for various meningitis patients, related to Figure 5.

	No. of subjects	No. of SERS spectra	Gender		Age in years (range)
			Male	Female	
Viral Meningitis	15	750	10(67%)	5(33%)	38±8(17-78)
Tubercular Meningitis	15	750	8(53%)	7(47%)	44±8(24-75)
Cryptococcal Meningitis	15	750	13(86%)	2(14%)	45±7(15-66)
Autoimmune Encephalitis	15	750	8(53%)	7(47%)	36±8(16-72)

Supplementary References

1. Sathyavathi, R. et al. Raman spectroscopy provides a powerful, rapid diagnostic tool for the detection of tuberculous meningitis in ex vivo cerebrospinal fluid samples. *J Biophotonics* **6**, 567-572 (2013).

2. Ye, M. et al. Subtype discrimination of acute myeloid leukemia based on plasma SERS technique. *Spectrochim Acta. Part A: Molecular and biomolecular spectroscopy* **271**, 120865 (2022).

3. Chen, S. et al. Predicting prognosis in acute myeloid leukemia patients by surface-enhanced Raman spectroscopy. *Nanomedicine* **16**, 1873-1885 (2021).

4. Chen, Y. et al. Optical tweezers and Raman spectroscopy for single-cell classification of drug resistance in acute lymphoblastic leukemia. *Journal of Biophotonics* **15**, e202200117 (2022).

5. Parlatan, U. et al. Label-Free Identification of Exosomes using Raman Spectroscopy and Machine Learning. *Small* **19**, e2205519 (2023).

6. Yu, Y. et al. Leukemia cells detection based on electroporation assisted surface-enhanced Raman scattering. *Biomed Opt Express* **8**, 4108-4121 (2017).

7. Maria Nowakowska, A. et al. Reliable cell preparation protocol for Raman imaging to effectively differentiate normal leukocytes and leukemic blasts. *Spectrochim Acta A: Mol Biomol Spectrosc* **292**, 122408 (2023).

8. Movasaghi, Z. et al. Raman Spectroscopy of Biological Tissues. *Applied Spectroscopy Reviews* **42**, 493-541 (2007).

9. Leszczenko, P. et al. Towards Raman-Based Screening of Acute Lymphoblastic Leukemia-Type B (B-ALL) Subtypes. *Cancers (Basel)* **13**, 5483 (2021).
